# Global transcriptional landscape and promoter mapping of the gut commensal *Bifidobacterium breve* UCC2003

**DOI:** 10.1186/s12864-017-4387-x

**Published:** 2017-12-28

**Authors:** Francesca Bottacini, Aldert Zomer, Christian Milani, Chiara Ferrario, Gabriele Andrea Lugli, Muireann Egan, Marco Ventura, Douwe van Sinderen

**Affiliations:** 1 APC Microbiome Institute and School of Microbiology, University College Cork, Cork, Ireland; 2 Department of Infectious Diseases and Immunology, Faculty of Veterinary Medicine, Utrecht University, Utrecht, the Netherlands; 3 Laboratory of Probiogenomics, Department of Chemical Sciences, Life Sciences and Environmental Sustainability, University of Parma, Parma, Italy

**Keywords:** Gene expression, Bifidobacteria, Probiotics, Transcription

## Abstract

**Background:**

*Bifidobacterium breve* represents a common member of the infant gut microbiota and its presence in the gut has been associated with host well being. For this reason it is relevant to investigate and understand the molecular mechanisms underlying the establishment, persistence and activities of this gut commensal in the host environment.

**Results:**

The assessment of vegetative promoters in the bifidobacterial prototype *Bifidobacterium breve* UCC2003 was performed employing a combination of RNA tiling array analysis and cDNA sequencing. Canonical −10 (TATAAT) and −35 (TTGACA) sequences were identified upstream of transcribed genes or operons, where deviations from this consensus correspond to transcription level variations. A Random Forest analysis assigned the −10 region of *B. breve* promoters as the element most impacting on the level of transcription, followed by the spacer length and the 5’-UTR length of transcripts. Furthermore, our transcriptome study also identified rho-independent termination as the most common and effective termination signal of highly and moderately transcribed operons in *B. breve*.

**Conclusion:**

The present study allowed us to identify genes and operons that are actively transcribed in this organism during logarithmic growth, and link promoter elements with levels of transcription of essential genes in this organism. As homologs of many of our identified genes are present across the whole genus *Bifidobacterium*, our dataset constitutes a transcriptomic reference to be used for future investigations of gene expression in members of this genus.

**Electronic supplementary material:**

The online version of this article (10.1186/s12864-017-4387-x) contains supplementary material, which is available to authorized users.

## Background

The development of Next Generation Sequencing (NGS) technologies has facilitated a genome-wide view of the transcriptional activities of an organism. The microarray-based technology for transcriptome analysis, which until recently was the tool of choice when assessing global transcription patterns of a given organism, has in recent years gradually been replaced by the alternative, NGS-based RNA-Seq approach [1].

Transcription levels in bacteria may vary considerably from gene to gene, and may also vary in response to (changes in) environmental conditions. In this context a key role is played by the RNA polymerase (RNAP) which is responsible for gene transcription. However, to initiate transcription RNAP requires (reversible) association with a sigma subunit (this complex is called the RNAP holoenzyme) in order to recognize the promoter sequence at two conserved DNA sequences that are located approximately 10 and 35 bp upstream of the transcriptional start site (TSS) [2, 3]. Once promoter recognition and transcription initiation has occurred, the sigma factor is released and RNAP (then referred to as RNAP core enzyme) proceeds with transcription.

In well characterized bacteria (such as *Escherichia coli* and *Bacillus subtilis*) several different sigma factors (between 7 and 10) have been identified, being responsible for global modulation of transcriptional patterns in response to changing growth conditions and environmental challenges [4, 5]. Extensive studies performed in *E. coli* have employed RNA sequencing (RNA-Seq) to identify and assess promoters recognized by the vegetative sigma-70 or RpoD sigma factor, which is responsible for transcription of housekeeping genes active during the exponential growth phase [3]. Transcription of such housekeeping genes is directed by constitutive promoters, which do not normally depend on particular transcription factors (TFs), and which consist of sequences that exhibit a high level of conservation [6].

Transcription termination in bacteria is caused by one of two principal mechanisms: *i*) rho-dependent transcriptional termination, which requires the presence of a stem-loop mediated pause site and the termination factor rho, and *ii*) rho-independent transcriptional termination, which involves a stem-loop structure followed by a poly-T sequence [7, 8]. Rho-independent transcriptional termination signals can be predicted by particular on-line tools such as ARNold, which employs RNAMotif and Erpin tools (http://rna.igmors.u-psud.fr/toolbox/arnold/) [9].

Bifidobacteria enjoy an ever increasing scientific interest due to the purported beneficial effects they elicit on their (human) host [10]. However, these gut commensals have remained relatively unexplored until recently due to their strict anaerobic metabolism and recalcitrance to genetic investigations [11, 12]. In order to investigate genetic features responsible for successful adaptation of bifidobacteria to the gut environment, we have used *Bifidobacterium breve* UCC2003 as a bifidobacterial prototype which has now become one of the most intensely characterized strains from a functional genomics perspective. Recently, genes have been identified which are essential for normal vegetative growth of this particular strain by employing a so-called TraDIS approach, which utilizes a mutant library of random Tn5 insertions combined with NGS to map the Tn5 insertion locations [13]. This study showed that the identified essential genes do not only represent housekeeping genes that constitute (part of) the core-genome, but that they may also represent non-conserved, strain-specific functions.

In the current study, we determined the global transcriptome of exponentially growing *B. breve* UCC2003 cells using two different approaches involving strand-specific tiling arrays and RNA-Seq analyses. The data generated from these analyses facilitated an in-depth investigation of the (vegetative) transcriptional landscape of this strain and reveal the principal features responsible for transcriptional initiation in this strain.

## Methods

### Array design and data analysis

Array probes that were 60 bp in length and overlapping with a 22 nt sliding window were designed across the forward and reverse strand of the *B. breve* UCC2003 genome sequence. In this manner an array containing a total of 230,722 probes (115,361 for either DNA strand) were obtained from Agilent Technologies and used for this study. Probes designed on known housekeeping genes, i.e. *dna*A, *dna*N, *rec*F, *gyr*B and *gyr*A [14], were used as hybridization controls for the arrays.

An overnight culture of *B. breve* UCC2003 was inoculated into 2% glucose MRS (Difco) medium, grown until mid-log phase (at which point an OD_600nm_ value of approximately 0.5 had been achieved) and harvested by centrifugation. Cell disruption followed by DNA/RNA isolation was performed as described in a previous study [15]. A total of 5 μg of bifidobacterial gDNA constituting the baseline was labelled with Cy3 (green channel) as previously described [15], with the following modifications. Prior to labelling, RNAse was removed using phenol/chloroform extraction followed by ethanol precipitation. Bacterial RNA was directly labelled following isolation without cDNA synthesis with Cy5 (red channel) using the Kreatech Agilent RNA labeling kit EA-023. Labelled gDNA and mRNA were then hybridized employing the Agilent Gene Expression hybridization kit (5188–5242) as described in the Agilent manual, Two-Color Microarray-Based Gene Expression Analysis (v4.0) (publication no. G4140–90050). Following hybridization, the arrays were washed and scanned using Agilent’s G2565A DNA microarray scanner. The obtained results were processed with Agilent’s Feature Extraction software (version 9.5) and further analysed with the Limma package in Bioconductor (https://www.r-project.org/) [16].

Background correction of raw data was performed using the convolution model (normexp + offset method in Limma) and a linear model with empirical Bayes statistics was employed to fit the log ratios and retrieve the highly expressed probes (mRNA) compared to the baseline (gDNA), as from the relative manual (https://www.bioconductor.org).

### RNA-Seq experiment

Total RNA was isolated from *B. breve* UCC2003 cultures grown in MRS (Difco) following the same protocol as mentioned above for the tiling array experiment. The obtained cell pellet was resuspended in 1 ml of QIAZOL (Qiagen, United Kingdom) and placed in a tube containing 0.8 g of glass beads (diameter, 106 μm; Sigma). The cells were lysed by shaking the mix on a BioSpec homogenizer at 4 °C for 2 min (maximum setting). The mixture was then centrifuged at 12,000 rpm for 15 min, and the RNA-containing upper phase was recovered. RNA was further purified by phenol extraction and ethanol precipitation [17]. Quality and integrity of the RNA was checked by the Tape station 2200 (Agilent Technologies, USA) analysis. RNA concentration and purity were evaluated by Picodrop microlitre Spectrophotometer (Picodrop, UK).

For RNA sequencing, 2.5 μg of total RNA was treated by the Ribo-Zero Magnetic kit (Illumina) to remove ribosomal RNA, followed by purification of the rRNA-depleted sample by ethanol precipitation. RNA was further processed according to the manufacturer’s instructions. The yield of rRNA depletion was checked by Tape station 2200 (Agilent Technologies). Then, 400 ng of the rRNA-depleted RNA sample was fragmented using a Bioruptor NGS ultrasonicator (Diagenode, USA) followed by size evaluation using Tape station 2200 (Agilent Technologies). A whole transcriptome library was constructed using the TruSeq Stranded RNA LT Kit (Illumina). Samples were loaded into a Flow Cell V3 150 cycles (Illumina) as reported by the technical support guide. The reads were depleted of adapters, quality filtered (with overall quality, quality window and length filters) and aligned to the *Bifidobacterium* reference genome through BWA [18]. Counting of reads whose sequences correspond to ORFs was performed using HTSeq (http://htseq.readthedocs.io/en/release_0.9.1/) and analysis of the RPKM values was performed using the formula RPKM = numReads/(geneLength/1000 ∗ totalNum-Reads/1,000,000) [19].

### Identification of promoters and transcriptional terminators

In order to define a transcriptional unit (TU) in *B. breve*, transcriptional start sites (TSSs) and termination sites (TTSs) were deduced from the combination of tiling arrays and RNA-Seq data. Array probes were first aligned to the full nucleotide sequence of *B. breve* UCC2003 (Genbank: NC_020517) using BLAT aligner [20] with masking of highly repeated regions (IS elements and transposases) so as to obtain start and end coordinates of each probe mapped to the *B. breve* UCC2003 genome. For each expressed gene or operon the start and end coordinates of the transcript were obtained from the first to the last base position from probes that elicit significant hybridization signals (Limma computed FDR *p*-value of 0.0001) at which mRNA signal discriminates from the gDNA baseline and compared to the RNA-Seq mapped reads. In the case of RNA-Seq transcriptional starts and ends were defined at the first and last base of reads where an increase or drop in sequence coverage was observed. The best fit to a canonical −10 and −35 promoter sequence in *B. breve* was first searched using Meme Suite (http://meme-suite.org/) over a region of 62 bp upstream of the TSS of a training set of 75 high/medium level transcribed housekeeping genes. The obtained predicted canonical promoter was then used to compile a promoter list for the remaining transcribed genes using a combination of Meme (http://meme-suite.org/) and manual annotation in Artemis (http://www.sanger.ac.uk/science/tools/artemis). All manually annotated promoters were first re-aligned with Meme to ensure that −10 and −35 were detected at their optimal position.

Rho-independent transcriptional terminators were first predicted using ARNold [21] and manually refined comparing tiling arrays signals and RNA-Seq alignment in Artemis (http://www.sanger.ac.uk/science/tools/artemis). Where appropriate, additional terminators to the ones predicted above were included following a manual search for the presence of polyT stretches downstream of putative stem-loop structures at the end of transcripts.

Parameters associated with the predicted promoter sequences were first retrieved and stored in variables to be used as classifiers in Random Forest (RF) analysis. Information contained in these classifiers was extracted from the promoter region (from 24 bp upstream of the −35 sequence, to 9 bp downstream of the −10 sequence) resulting in a 62 basepair region (on average), which was manually aligned with the predicted transcription start and the −10 and −35 regions. Random forest (RF) analysis was then performed using the RandomForest v4.6–10 package in R (https://cran.r-project.org). Random forest classification was performed to identify signature classifiers for discrimination of not-expressed, low, medium and highly expressed genes. This classification model, consisting of 5000 decision trees was trained on random subsets of properties and sequences of promoter regions of expressed genes and 270,000 promoter-sized kmers in the intergenic region of *B. breve* UCC2003 (excluding the promoters) as negative control. The classifiers chosen for RF were: spacer length (bp), leader length (distance between promoter and start of the gene), AT % of the −35 upstream region, AT % of the −35 signal, AT% of the spacer, AT % of the −10 region and AT % of the −10 downstream region and all aligned bases in the 62 bp promoter region. Three classes were chosen based on the differential hybridization level between mRNA signal vs gDNA baseline: high (>10 fold), medium (3 < 10 fold) and low (<3 fold) level of transcription.

### Matching predicted core and/or essential genes with transcriptomic data

In order to assess if the identified transcribed genes are part of the *B. breve* core-genome and/or conserved across the *Bifidobacterium* genus, information from comparative genome analysis was integrated into our transcriptome dataset. Deduced amino acid sequences from the identified ORFs of *B. breve* UCC2003 were compared using BLASTP (e-value for significance: 0.0001) with orthologues previously retrieved from seven fully sequenced and publicly available *B. breve* genomes as well as 46 *Bifidobacterium* type strains [22–24].

In order to combine our expression data with information related to essential *B. breve* UCC2003 genes, the list of transcribed genes in our datasets was compared with genes that were deemed essential based on analysis of an insertional mutant library of this strain combined with TraDIS sequencing [22]. A circos plot was then generated for data visualization and circular representation of results (http://circos.ca/).

### Prediction of small RNAs

Prediction of sRNAs and RNA-based regulatory elements was performed using the RNAspace web server (http://www.rnaspace.org/) and the Rfam database (http://rfam.xfam.org/) and the entire nucleotide sequence of *B. breve* UCC2003 as input.

Artemis v16 (http://www.sanger.ac.uk/science/tools/artemis) was used to inspect the results and for data representation.

## Results and discussion

### Tiling arrays and RNA-Seq alignment

The transcriptome of exponentially growing *B. breve* UCC2003 cells, when cultivated under standard laboratory conditions (growth in liquid MRS medium supplemented with 2% glucose), was determined in this study using two different technologies involving a hybridization-based approach availing of whole-genome tiling arrays integrated with data obtained by (Illumina) high throughput RNA sequencing (Additional file 1: Table S1) (Additional file 2: Figure S1 panel a).

**Fig. 1:**
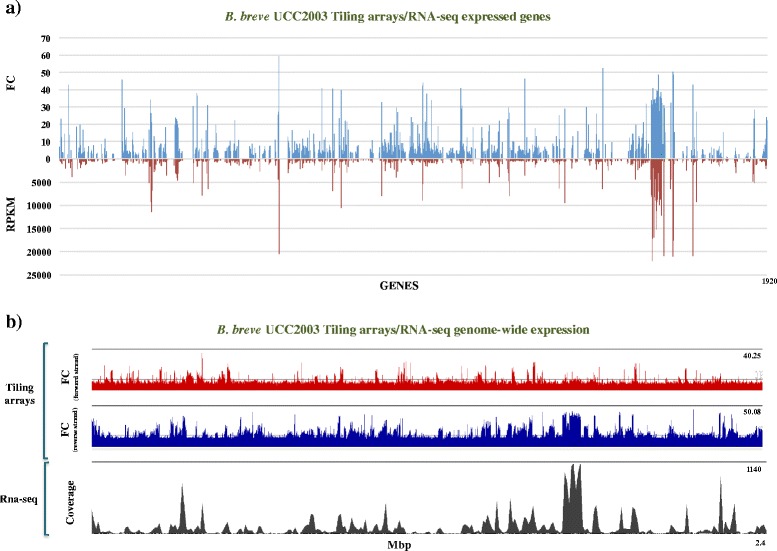
Tiling arrays and RNA-Seq alignment. Genome-wide transcription of the *B. breve* UCC2003 chromosome obtained using a combination of tiling array and RNA-Seq technologies. **a** Alignment of transcription patterns of *B. breve* genes (ORFs) as obtained from tiling array (blue) or RNA-Seq (red) data. Expression levels are indicated as RPKM (Reads Per Kilobase of transcript per Million mapped reads) in RNA-Seq dataset and FC (Fold-Change expressed as RNA signal strength vs gDNA baseline) in tiling arrays. **b** Genome-wide transcription of the *B. breve* UCC2003 chromosome as determined by a strand-specific tiling array (red for the forward and blue for the reverse strand) or RNA-Seq (grey)

The analysed data obtained from the tiling arrays revealed that a total of 852 coding sequences (CDSs), as well as 47 tRNA and 6 rRNA genes were transcribed under the applied conditions in *B. breve* UCC2003. Comparison of the micro array-mediated transcriptome output with the transcriptome as determined by sequencing data (RNA-Seq) showed that 84.3% of the genes identified as being transcribed in the tiling array approach were also identified by the RNA-Seq approach, confirming good reproducibility between these two technologies (Fig. 1 panels a & b). However, it is worth mentioning that the RNA-Seq dataset permitted the identification of 265 additional genes, which had not been detected as significantly transcribed in the tiling arrays (Additional file 1: Table S1). As the majority of these genes shows RPKM values around the cut-off of 150 RPKM, this suggests that either tiling array data are more affected by background hybridization signals across the genome or that RNA-Seq data lead a higher rate of false positives (Additional file 2: Figure S1 panels a & b).

**Fig. 2:**
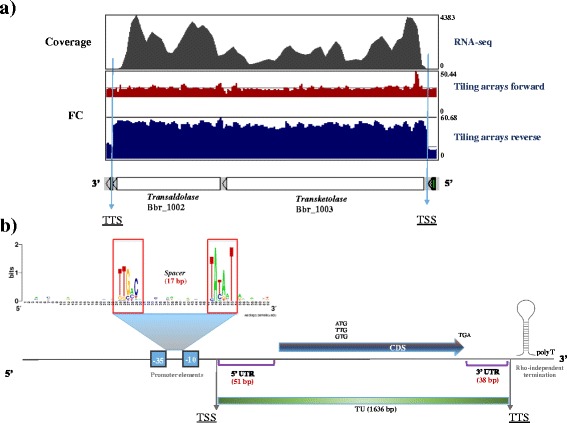
*B. breve* transcriptional unit organization. Characteristic elements of a typical, highly expressed TU in *B. breve*. **a** Comparative transcription patterns of highly transcribed TU encoding the transaldolase/transketolase proteins. Directional tiling arrays signals are expressed in red (forward strand) and blue (reverse strand), while RNA-Seq expression levels are indicated in grey. From this representation it is possible to appreciate the non-even distribution of RNA-Seq reads across the transcript as compared to the array signals. **b** Schematic representation of a bifidobacterial TU with relative main features (e.g. the promoter elements −10 and −35 located upstream a TSS as well as the TTS (rho-independent transcriptional termination)

Despite the above mentioned corresponding data generated by the two distinct transcriptome methods, some differences were observed. For example, we observed a non-even distribution of RNA-Seq reads across the same transcript, which is not (or much less so) noted for the tiling array approach, where the hybridization signals seem to be relatively evenly spread across a single transcript (Fig. 2 panel a). This difference can be explained by several factors known to affect RNA-Seq technology, such as the *i*) presence of mRNA secondary structures influencing the cDNA synthesis, *ii*) ambiguity in read mapping, *iii*) random hexamer priming in cDNA generation which biases the nucleotide composition at the start of sequencing reads, and/or *iv*) fragmentation during library preparation [25–27]. An advantage of the tiling array approach, as applied here, is that due to the direct labelling of mRNA (which therefore does not require cDNA synthesis) the obtained data is less affected by positional biases. What we observed in the tiling array approach is a more pronounced and consistent signal along individual transcripts (Fig. 2 panel a), also allowing a more accurate classification of genes based on fold-change or FC (level of RNA signal strength vs gDNA baseline) compared to RPKM [28].

The 22 bp sliding window used to design the array probes introduced a degree of uncertainty in the determination of the exact transcriptional start site (TSS), although a precise location of the TSS could in most cases be verified by RNA-Seq information (see below). Despite the high level of concordance obtained between the tiling array and RNA-Seq data sets, we found higher reproducibility for those genes exhibiting high or medium transcription levels. In fact, genes eliciting a low transcriptional level in tiling arrays were sometimes hard to identify as their hybridization signals were very close to baseline signal levels.

**Fig. 3:**
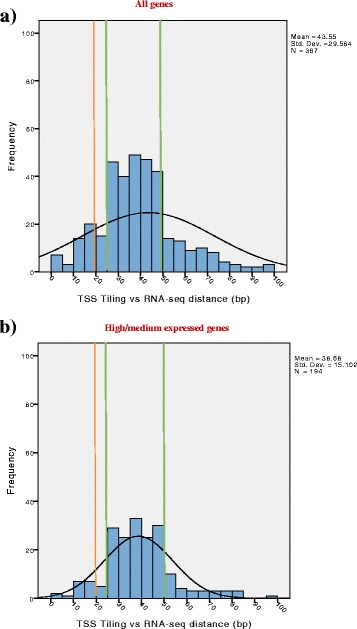
Tiling arrays and RNA-Seq accuracy. Evaluation of the obtained accuracy in determining the exact position of TSSs using tiling array and RNA-Seq data. **a** Barplot showing the discrepancy between TSSs predicted by tiling array or RNA-Seq data as based on all genes. A green line indicates the ranges of detection with higher frequency of hits, while an orange line indicates the expected discrepancy value. From this chart it is possible to assess how the discrepancy in predicting a TSS ranges between 25 and 50 bp. **b** Discrepancy between TSS predicted by tiling array and RNA-Seq approaches when based only on high and medium transcribed genes. A green line indicates the ranges of detection with higher frequency of hits, while an orange line indicates the expected discrepancy value. As from the chart is possble to observe a decrease of discrepant predictions located at the extremities of the distribution when the lowly transcribed genes are removed

With the objective of defining TSSs of vegetatively transcribed genes and operons in *B. breve*, array and RNA-Seq data were first independently processed and subsequently compared. In the specific case of tiling arrays, TSSs were assigned at the first base position of the first significantly expressed probe in the 5’-UTR region of a transcript. As the tiling array employed in the current study was designed based on a 22 bp sliding window, this was inevitably going to impact on the TSS-assignment accuracy. For this reason TSSs were also determined from RNA-Seq data and compared, which revealed that the vast majority of discrepancies between the obtained predictions ranged between 25 and 50 bp, where a given TSS predicted from RNA-Seq was always located downstream of the corresponding array-based TSS. As the arrays were designed based on 60 bp probes with 22 bp overlap, we expected an overestimation of TSS in tiling arrays of at least 22 bp. Our comparison of TSS prediction performed with these two approaches revealed that the associated error of predicting TSSs from our array design does not exceed the size of one probe (Fig. 3 panel a). A further comparison of our result with the TSS experimentally determined for three housekeeping genes in *B. breve* UCC2003 (*hrc*A:Bbr_1004, *clp*C:Bbr_1356 and *gro*ES:Bbr_1668) [29–31] returned a prediction error of 13, 23 and 32 bp respectively, which is also comparable with our predicted error. Interestingly, when we exclude weakly transcribed genes, we noticed a decrease of discrepant predictions located at the extremities of the distribution, confirming the suspected higher impact of weakly transcribed genes in TSS misassignments (Fig. 3 panel b). Weakly transcribed genes may be affected by factors such as higher background interference, lower mRNA stability or low number of guanines in probes (labelled at N7 position), making the associated predictions in some cases inconsistent.

### Transcriptional initiation and termination in *B. breve*

Integration of data generated using tiling arrays and RNA-Seq platforms allowed us to determine the (approximate) TSS of 413 genes and/or operons (including 2 rRNA loci and 33 tRNAs) transcribed in *B. breve* during exponential growth.

In order to establish whether such determined TSSs would allow us to predict a promoter consensus sequence for *B. breve*, the alignment of a 62 bp region upstream each determined TSS was performed followed by conserved motif finding in Meme (http://meme-suite.org/). This search was carried out with the idea of covering the binding region required for the RNA polymerase to initiate transcription [3, 32].

Our analysis identified typical bacterial vegetative promoter consensus motifs of TATAAT (−10 region) and TTGACA (−35 region), located within an optimum of 17 bp spacer distance, being especially conserved upstream of highly/medium expressed (housekeeping) genes (Fig. 2 panel b).

Analysis of the promoter sequences in all identified TSSs returned putative −10 and −35 regions with a certain degree of degeneration with a predicted spacer region rarely exceeding the 20 bp or being shorter than 14 bp. We also observed a higher tendency of conservation of the −10 element compared to the −35. The −10 TATA box in fact appeared to be the most conserved motif with thymine in 1^st^, adenine in 2^nd^ and thymine in 6^th^ position (Fig. 2 panel b). Regarding the −35 signal, despite a weaker degree of conservation across the assessed promoters, thymine in 1^st^ and 2^nd^ position seemed to be the most recurrent bases, followed by guanine in 3^rd^ and cytosine in 5^th^ position (Fig. 2 panel b).

Despite the finding that the intergenic regions are usually more AT rich as compared to the coding regions, our analysis did not identify a clear consensus sequence within 60 bp upstream the −10 TATA box, which seems to be the dominant low G + C signal upstream each TSSs.

As a further verification, we wanted to establish the average distance between the predicted conserved promoter motifs and the TSSs identified by RNA-Seq, setting the optimum expected distance to 10 bp. Our analysis revealed an average distance between predicted promoter and TSSs of 20 bp, suggesting that TSS prediction based on RNA-Seq data is still missing bases at the 5’-UTR, in line with what has previously been described for this technology in bacteria [33]. Nevertheless, the precision achieved by our sequencing (also confirmed by the comparison with the tiling array coordinates) allowed us to successfully detect (bifido)bacterial vegetative promoters (Additional file 3: Table S2).

In order to obtain a better characterization of bifidobacterial transcriptional units (TUs), the identification of transcriptional start sites (TSS) was followed by the analysis of putative transcriptional termination sites (TTSs).

In the case of transcriptional termination, we found that the array data represented the 3′ termination signal of operons better than the RNA-Seq information (and for this reason we did not include the latter data when predicting TTSs), probably because the tiling array data were based on direct mRNA labelling, without the necessity of cDNA synthesis, and for this reason these results are not affected by interfering factors such as 5′ enrichment of reads and/or uneven distribution of coverage along a single transcript (Fig. 2 panel a).

Based on our analysis, we were able to identify a total of 224 rho-independent terminators (accounting for 54% of the total of termination signals), each constituted by a GC-rich stem loop structure followed by the characteristic polyT region. In the case of bi-directional terminators we observed that the stem loop region is preceeded by polyA [9].

**Fig. 4:**
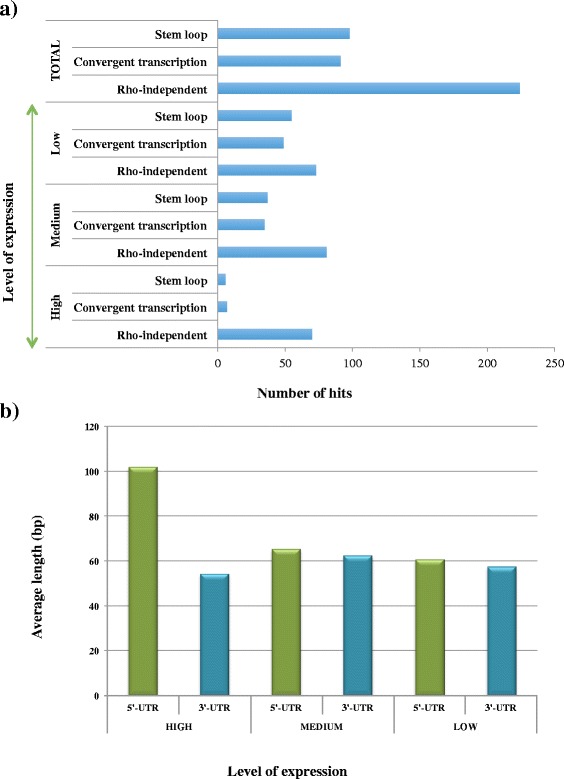
*B. breve* transcriptional unit elements and termination. **a** Barplot showing the different strategies in transcriptional termination identified in *B. breve* UCC2003, grouped by level of transcription (high, medium and low). From the Barplot it is possible to observe the high occurrence of rho-independent termination in this organism. **c** Barblot comparing the length of 5’-UTR and 3’-UTR to genes with transcription level high, medium and low. From the chart it is possible to observe a higher 5’-UTR length in highly transcribed genes as compared to gene with a medium and low level of transcription

Of the 413 identified TUs, 83 were classified as highly transcribed (see Methods section) of which 70 contained a rho-independent terminator, while of the 153 classified as moderately transcribed TUs, 81 were predicted to be subject to rho-independent termination. As regards to weakly transcribed TUs, only 73 terminators out of the 177 identified were classified as rho-independent, suggesting that rho-independent terminators represent strong termination signals especially for bifidobacterial genes and operons exhibiting high (and perhaps medium) level transcription (Fig. 4 panel a). Rho-independent termination is also typically found at the end of highly transcribed TUs (e.g. rRNA and tRNA specifying genes) of other bacteria such as *E. coli* and *Bacillus subtilis* [9, 34].

As previously reported, rho-independent terminators preceeded by polyA and followed by polyT sequences may be responsible for controlling transcriptional termination of convergently transcribed operons/genes within a given DNA region (Additional file 4: Figure S2 panel a). However, we also noticed that the lack of a polyA sequence in these elements may restrict the termination to one strand only (Additional file 4: Figure S2 panel b) [8].

As rho-independent terminators seem to represent the most frequently adopted strategy by *Bifidobacterium breve* UCC2003 to achieve transcriptional termination (especially of highly transcribed operons), we also observed that TTSs often locate at GC-rich stem-loops structures, not otherwise classified as rho-independent terminators. It can be argued that they constitute rho-dependent terminations or RNA polymerase pausing sites prior to the release of the transcription machinery, even though we did not identify a clear consensus among these regions. Nevertheless this observation suggests a possible involvement of GC-rich stem-loops as pausing sites for the RNA polymerase followed by its release.

In the case of convergently expressed operons (simultaneously transcribed in a tail-to-tail orientation), we often observed that no termination signal was detectable. However, in these cases we often noticed overlap between the 3’-UTR region of these opposing transcripts (Additional file 4: Figure S2 panel c). It has been shown that transcriptional interference or gene silencing can occur in convergently expressed operons when their 3’-UTR regions partially overlap. This phenomenon is caused by the direct collision and release of the transcriptional machinery progressing along either strand, resulting in random transcriptional termination that may extend into the adjacent operon [35]. Our findings therefore indicate that also in bifidobacteria convergent transcription is a strategy to modulate gene expression.

The transcriptome analysis performed in this study also allowed an investigation into the main features associated with TUs in *B. breve* (which may also apply to bifidobacteria in general). Deduced (vegetative growth-associated) TU length in *B. breve* was shown to vary from over 9 Kbp to ~200 bp (when constituted by a single gene), with a calculated average size of 1636 bp.

From a closer look at *B. breve* TUs, the 5’-UTR region is nearly always longer than the untranslated 3′ end (average of 51 bp vs 38 bp, respectively) (Fig. 4 panel b). Interestingly, if we associate this observation to a further classification based on transcription level (see Methods section), the average 5’-UTR length of highly transcribed genes (~100 bp) is significantly longer than the respective 5’-UTR in medium and low transcribed ones (~60 bp), suggesting possible regulatory/activating roles for these UP (upstream) regions (Fig. 4 panel b).

### Promoter strength and level of expression

With the aim of investigating factors that have the most obvious impact on the level of gene transcription in *B. breve*, the information obtained from the identification of promoters was stored in variables and used to generate classifiers for Random Forest (RF) analysis.

The classifiers chosen for RF were based on spacer length (bp), 5’-UTR length (distance between promoter and start of the gene), AT % of the −35 and corresponding upstream region, AT % of the −35 signal, AT% of the spacer, AT % of the −10 region and AT % of the −10 downstream region.

All promoters were grouped based on the level of RNA signal strength vs gDNA baseline (expressed as fold-change or FC). Such analyses revealed transcription levels being classified as high (FC > 10), medium (3 < FC < 10) or low (FC < 3), which were then subjected to RF (Random Forest) analysis performed on 5000 trees (representative of the whole sequence space).

**Fig. 5:**
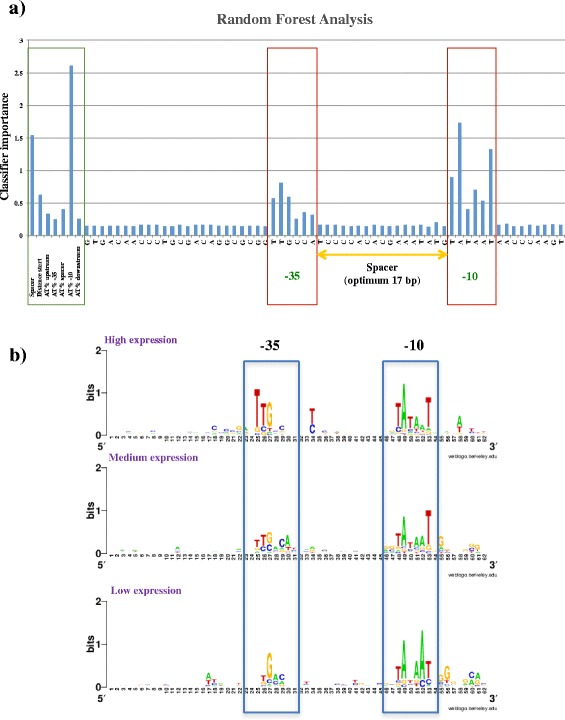
Promoter strength. Results of the Random Forest (RF) computation and analysis of the variation of promoter consensus across various transcription levels in *B. breve* UCC2003. **a** Barplot showing the results of the RF analysis conducted on 62 bp regions upstream of predicted TSSs. The plot shows the importance of each base position across the 62 bp region where the promoter elements (−10 and −35) can be detected (red blocks). Importance values of a number of additional classifiers is also indicated (green block). **b** Weblogo showing the consensus motif identified for the −10 and −35 sequences of the canonical promoter in *B. breve* across the levels of expression high, medium and low. Also in this case the promoter elements (−10 and −35) can be detected

Consistent with what we observed for the promoter consensus analysis, RF approach confirmed that the AT % of the −10 region shows the highest impact on gene transcription, immediately followed by the length of the spacer region (of which the optimum was established to be 17 bp). Our analysis also revealed that the 5’-UTR length may impact gene transcription, in fact a longer leader length seems to be associated with highly expressed genes (Fig. 5 panel a). Regarding the promoter elements, RF analysis returned highest importance in 1^st^, 2^nd^ and 6^th^ position for the −10 region, while the −35 region showed higher importance in the first three base positions despite having less impact on gene transcription (Fig. 5 panel a).

Unfortunately, our attempts to employ RF analysis to predict the level of transcription of a promoter based on these classifiers returned a high error rate (~66%), suggesting that the de novo prediction of transcription level cannot be deduced from the promoter with sufficient accuracy. However, our analysis highlighted certain trends in the degree of conservation of the promoter. Highly transcribed genes seem to possess a promoter that best resembles the canonical bifidobacterial consensus, while medium expressed genes possess a more degenerate −35, but a still highly conserved −10 region. Interestingly, weakly transcribed genes seem to possess both degenerated −10 and −35, with an AT-rich region located in the TATA box (Fig. 5 panel b). This is also consistent with our result in RF analysis showing that the GC content of the −10 region is the classifier that most substantially impacts on transcription in *B. breve*.

### Transcription of essential genes in *B. breve*

**Fig. 6:**
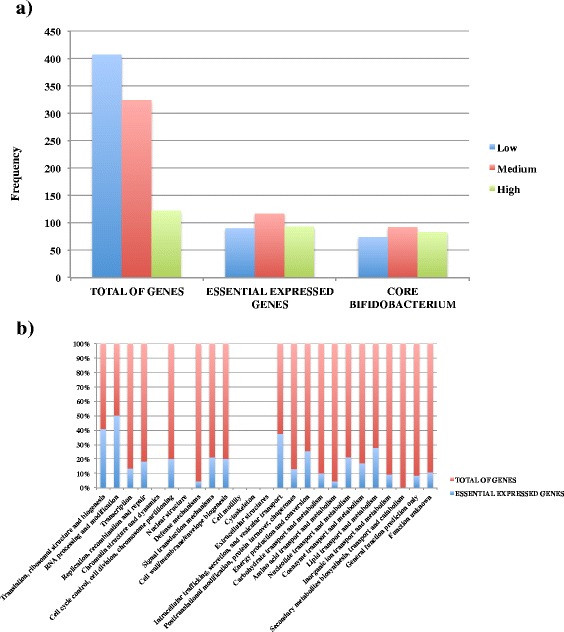
Essential expressed genes. Analysis showing the overlap between transcription level in core and essential genes. **a** Barplot showing the distribution of lowly, medium and highly transcribed genes of the essential genes of *B. breve* UCC2003 and the core genome of *Bifidobacterium* compared to the whole transcriptome (total of genes). The chart also illustrates the overlap between the core and essential transcribed genes in this organism. **b** Stacked-column plot showing the percentage of essential (blue) and total (red) expressed genes organized by COG categories. The plot displays how housekeeping COG categories (e.g. translation, ribosomal structure and biogenesis as well as RNA processing and modification) are the most represented in our dataset

To further analyse the obtained transcriptome information, we wanted to combine the results of our transcriptomics findings with TraDIS sequencing findings conducted to determine the essential genes in *B. breve* [22]. The alignment with this dataset was conducted based on the presumption that genes essential in *B. breve* should also be expressed in our transcriptomic data. Of the 854 CDSs identified in our transcriptome analysis, 35% also appear to be essential for the survival of *B.breve* UCC2003. These genes also appear homogeneously distributed between high, medium and low level of expression. However, 76% of the total of highly expressed genes is also essential in *B. breve*, confirming the hypothesis that the vast majority of essential housekeeping genes also appear to be highly expressed in *B. breve* (Fig. 6 panels a & b) (Additional file 5: Table S3). An alignment across the whole genus also showed that orthologs of such expressed genes can be found across the genus *Bifidobacterium*, in support of the notion that our findings can be used as a starting point for the investigation of (regulation of) gene expression as applied to the whole genus (Fig. 6 panel a).

From a closer inspection of the COG categories, it also appears that between 40% and 50% of those essential and highly transcribed genes in *B. breve* are assigned to functions involved in “Translation, ribosomal structures and biogenesis”, “RNA processing and modification” as well as “Intracellular trafficking and secretion” (Fig. 6 panel b).

It is furthermore worth mentioning that some essential and highly expressed housekeeping genes in *B. breve* appear to be co-transcribed and organized in operons of considerable dimension. This is the case for example of the 9.5 Kbp ribosomal operon (Bbr_1622–42) and the 7.2 Kbp ATP synthase operon (Bbr_0323–30), both characterized by an untranslated leader region of remarkable length (210 and 157 bp, respectively). Another notable example of essential TUs is represented by the ribosomal rRNA genes, organized in two 5.6 Kb regions in *B. breve* UCC2003 and exhibiting a transcription level that is higher than any other gene (>50 fold) in the genome (Additional file 6: Figure S3 panel a). Also, in this case the 5′ untranslated leader region identified by our transcriptome analysis is, at 281 bp, the longest identified in this organism. Together with the expression of two rRNA loci in *B. breve* UCC2003, our analysis also confirmed the constitutively high transcription of 47 of a total of 53 predicted transfer RNA genes specified by *B. breve* UCC2003 (Table 1). These predicted tRNAs are organized in 38 loci evenly distributed along the bacterial chromosome and their essentiality for this organism has previously been reported [22]. The alignment of our transcriptome data with the TraDIS-mediated essential gene outcome confirmed a constitutive expression for the tRNA-encoding genes specific for 20 amino acids in *B. breve*, especially in case where multiple copies of these genes (>3 copies) are present (tRNAs encoding for leucine, glycine, arginine, valine, serine and alanine) (Additional file 5: Table S3).

**Table 1:** Transfer RNA transcription in *B. breve*

Locus_tag	Annotation
Bbr_tRNA1–2	tRNAs Ala /Ile
Bbr_ tRNA3	tRNA Leu
Bbr_ tRNA4	tRNA Gly
Bbr_ tRNA5–6	tRNAs Glu /Gln
Bbr_ tRNA7	tRNA Ser
Bbr_ tRNA8	tRNA Lys
Bbr_ tRNA9	tRNA Lys
Bbr_tRNA11	tRNA Met
Bbr_ tRNA12	tRNA Arg
Bbr_ tRNA13	tRNA Gly
Bbr_ tRNA14–15	tRNAs Leu /Thr
Bbr_ tRNA16	tRNA Arg
Bbr_ tRNA17	tRNA His
Bbr_ tRNA19	tRNA Leu
Bbr_ tRNA20	tRNA Leu
Bbr_ tRNA22	tRNA Gln
Bbr_ tRNA23–24	tRNAs Ala
Bbr_ tRNA25–26	tRNAs Arg
Bbr_ tRNA27	tRNA Leu
Bbr_ tRNA28–32	tRNAs Gly/Cys/Val
Bbr_ tRNA33	tRNA Pro
Bbr_ tRNA34–35	tRNAs Asn
Bbr_ tRNA38	tRNA Asp
Bbr_ tRNA39–40	tRNAs Phe/Asp
Bbr_ tRNA41	tRNA Glu
Bbr_ tRNA42	tRNA Pro
Bbr_ tRNA43	tRNA Ser
Bbr_ tRNA44	tRNA Ser
Bbr_ tRNA45	tRNA Ser
Bbr_ tRNA47–48	tRNAs Thr/Tyr
Bbr_ tRNA49–51	tRNAs Val/Gly
Bbr_ tRNA52	tRNAs Trp
Bbr_ tRNA53	tRNA Ala

Another intriguing feature revealed by our dataset is the (constitutive) transcription of the CRISPR/Cas spacers in *B. breve* UCC2003. This system represents a defense mechanism of *B. breve* and is aimed at preventing the acquisition of foreign DNA, such as phages and mobile elements. Although this region has not been identified as essential for survival, our transcriptome data shows that a continuous surveillance of this defense mechanism remains activated in *B. breve* also under standard laboratory conditions, suggesting that this system is active (Additional file 6: Figure S3 panel b). Along with CRISPR/Cas genes, Restriction/Modification (R/M) systems play a significant role in evading the acquisition of foreign DNA in *B. breve* UCC2003, which is equipped with two complete (BbrII, BbrIII) and one partially functioning (BbrI) systems [36]. Of these three, it has been shown that only the methylase component is essential for bacterial survival [22]. We observed that all these genes are transcribed at low level (~2 fold above background level) in our dataset (Bbr_0216, Bbr_1119 and Bbr_1121), apparently ensuring a permanent default protection of the bacterial chromosome from their partner endonucleases (Additional file 5: Table S3).

### Housekeeping sRNA transcription and riboswitches in *B. breve*

The genome-wide transcriptional analysis conducted in this study allowed the annotation of a set of intergenic regions that may encompass sRNAs with housekeeping and/or regulatory functions in *B. breve* such as RNAseP, transfer-messenger RNA (tmRNA) and 4.5S RNA (Table 2).

**Table 2:** Novel sRNA identified in *B. breve*

sRNA	Genome coordinates (UCC2003)	Strand	Annotation
RNAseP	1,474,615–1,474,960	forward	Ribonuclease P
tmRNA	1,563,425–1,563,820	forward	Transfer messenger RNA
4.5S RNA	251,225–251,328	forward	Signal recognition particle RNA (SRP)
FMN riboswitch	1,655,817–1,655,970	forward	Flavin mononucleotide riboswitch
TPP riboswitch	886,294–886,401	forward	Thiamine pyrophosphate riboswitch (T-box)
YKOK leader	541,771–541,928	reverse	Metal-sensing RNA (M-box)

Based on our analysis we assigned RNAseP to a 346 bp highly transcribed intergenic region (>50 fold) with associated promoter and rho-independent terminator (Additional file 3: Table S2)(Additional file 7: Figure S4 panel a). With regards to the transfer-messenger RNA (tmRNA) (Table 2) we assigned this element to a 396 bp highly transcribed intergenic region (>50 fold), also in this case characterized by a promoter and a rho-independent termination signal (Additional file 3: Table S2, Additional file 7: Figure S4 panel b).

In the case of signal recognition particle RNA (SRP) or 4.5S RNA we assigned this sRNA to a 104 bp highly transcribed region (>50 fold) and we were able to identify the corresponding promoter while the associated transcriptional terminator is characterized by a stem loop structure (Additional file 3: Table S2, Additional file 7: Figure S4 panel c). It is worth mentioning that the alignment of this dataset with the TraDIS outcome [22] showed that no insertions are present in these particular regions, indicating that these regions are essential to *B. breve* (Additional file 7: Figure S4 panels a-c).

As regards small regulatory RNA elements, our analysis identified three previously not characterized in bifidobacteria: a flavin mononucleotide (FMN), a thiamine pyrophosphate (TPP) or T-box and a YKOK riboswitch. In the case of the identified FMN riboswitch, we located the expression of this element in the 5’-UTR region of two genes (locus tags Bbr_1328–29) resembling the modular organization of an energy coupling factor (ECF) transporter [37] (Additional file 8: Figure S5 panel a). Of these Bbr_1328 belongs to the COG3601 family of “riboflavin transport”, while Bbr_1329 constitutes an ABC transporter and ATPase of a putative ECF, suggesting the involvement of these genes and FMN riboswith in the transport of this particular B vitamin.

In the case of TPP-sensing riboswitch or T-box we assigned it to an expressed 5’-UTR region of an operon involved in thiamine biosynthesis (Bbr_0674–77), suggesting also in this case its involvement in the regulation of these genes (Additional file 8: Figure S5 panel b).

Finally, in the case of the YKOK leader or M-box (Mg^2+^ metal-sensingRNA) we identified this element in an expressed region located at the 5’-UTR of an ABC transporter (Bbr_0406–07), where the permease-encoding gene (Bbr_0407) belongs to the category COG4986 of “inorganic ion transport”, suggesting also in this case the involvement of these genes in bacterial metal ion homeostasis (Additional file 8: Figure S5 panel c).

Altogether these observations show the expression of a number of housekeeping RNAs and regulatory RNA elements in *B. breve*, suggesting the possibility of a new level of RNA-mediated regulation of gene expression also in members of this genus.

## Conclusions

Comparison of transcriptomic data obtained by tiling arrays and RNA-Seq showed reproducibility between these two technologies, but also revealed the benefit of using a combination of these two approaches in investigating gene expression. An advantage of using the hybridization-based technology of tiling arrays is the fact that it is less affected by positional biases, producing a consistent expression signal along transcripts. On the other hand the RNA-Seq dataset detected a higher number of transcribed genes and appeared less affected by background hybridization signals across the genome.

Based on the obtained transcriptome data we were able to map transcriptional start and termination sites (TTS) of the identified *B. breve* transcriptional units relevant to logarithmic growth. The obtained dataset allowed us to identify a typical bacterial consensus of TATAAT (−10 rgion) and TTGACA (−35 region) with an optimum spacer length of 17 bp upstream the TSS of transcribed genes or operon. Random Forest analysis revealed the parameters with the highest impact on transcription levels in *B. breve*, being the AT % of the −10 region the classifier with highest importance followed by the spacer length and the 5’-UTR length of transcripts. Our analysis highlights how the consensus of the promoter region appears to degenerate from the canonical consensus with the decrease in transcriptional level, however, prediction of transcription levels is still difficult and may require the inclusion of other (structural) features of the promoter region.

Our study also described how rho-independent termination represents the most common and effective termination signal adopted by *B. breve* (and perhaps *Bifidobacterium*), especially for highly and moderately transcribed operons. It also showed that there may be other strategies of transcriptional termination responsible for modulating gene expression in *B. breve*.

Furthermore, the alignment of our dataset with a recently published study on the essential genes of *B. breve* demonstered how the vast majority of those also appear to be expressed in our dataset, in particular those housekeeping genes of which orthologues can be found across the *Bifidobacterium* genus.

Finally, our analysis also allowed the identification of a number housekeeping sRNAs and regulatory RNA elements not previously identified in *B. breve* UCC2003 (or other bifidobacterial species), indicating that RNA-mediated regulation of gene expression also occurs in this organism (and genus). Altogether this study has generated a detailed and robust dataset to be used as a reference for transcription in the genus *Bifidobacterium*.

## Additional files

Additional file 1: Table S1. Transcribed genes as determined by RNA-Seq and tiling array analyses. A .docx document containing the list of genes detected as expressed in RNA-Seq and Tiling arrays experiments. For each gene the fold-change (FC expressed as level of RNA signal strength vs gDNA baseline) and RKPM values are also indicated. (DOCX 135 kb)

Additional file 2: Figure S1. RNA-Seq and tiling array comparisons. **a)** Bar chart showing *B. breve* UCC2003 genes detected as transcribed in RNA-Seq, but not in tiling arrays with associate gene count and level of expression (RPKM). **b)** Distribution of genes exhibiting discrepant transcription between RNA-Seq and tiling array approaches as grouped by level of transcription (RPKM). A red horizontal line indicates the baseline of transcription background, while in purple the average RPKM of transcribed genes is indicated. (PDF 94 kb)

Additional file 3: Table S2. Predicted transcriptional units (TUs) in *B. breve* and associated promoters. A .docx document containing the list of predicted transcriptional units (TUs) in *B. breve* and corresponding (predicted) promoters. For each TU also the transcription level and transcriptional termination is indicated. (DOCX 143 kb)

Additional file 4: Figure S2. *B. breve* termination of transcription. Artemis plot showing the different strategies of transcriptional termination of convergently expressed genes (dashed purple line) in *B. breve*: **a)** Double-stranded rho-independent termination observed between two convergently expressed genes employing a bidirectional terminator. In this case a clear-cut transcriptional termination is observed in either forward and reverse strands. **b)** Single stranded rho-independent termination observed between two convergently transcribed genes employing a strand specific terminator. In this case transcriptional termination is only observed in the forward strand. **c)** Tail-to-tail termination observed between two convergently transcribed genes without a termination signal. In this case no specific point of transcriptional termination can be observed. (PDF 474 kb)

Additional file 5: Table S3. Transcription of essential genes in *B. breve*. A .docx document containing the list of essential genes of *B. breve* UCC2003 with associated level of transcription as detected from our transcriptomic study. (DOCX 41 kb)

Additional file 6: Figure S3. *B. breve* ribosomal operon and CRISPR-Cas system transcription. Artemis plot showing the level of transcription of **a)** the rRNA operon and **b)** the CRISPR/Cas system in *B. breve* UCC2003 as detected in tiling arrays. The relative TU is indicated by a dashed purple line. (PDF 282 kb)

Additional file 7: Figure S4. *B. breve* sRNA expression. Artemis plot showing the sRNA transcription in *B. breve* of **a)** Ribonuclease P, **b)** tmRNA, and **c)** 4.5S SRP RNA. In all cases tiling array signals of forward (red) and reverse (blue) strand are indicated. (PDF 701 kb)

Additional file 8: Figure S5. *B. breve* regulatory RNA expression. Artemis plot showing the regulatory RNA transcription in *B. breve* of **a)** FMN, **b)** TPP, and **c)** YKOK elements. In all cases tiling array signals of forward (red) and reverse (blue) strand are indicated. (PDF 732 kb)

## Abbreviations

ATP: Adenosine triphosphate
CDS: Coding sequence
COG: Clusters of Orthologous Groups
CRISPR: Clustered Regularly Interspaced Short Palindromic Repeats
DNA: Deoxyribonucleic acid
ECF: Energy Coupling Factor
FDR: False Discovery Rate
FMN: Flavin mononucleotide
gDNA: Genomic DNA
mRNA: Messenger RNA
MRS: De Man Rogosa and Sharpe medium
NGS: Next Generation Sequencing
ORF: Open Reading Frame
R/M: Restriction/Modification
RF: Random Forest
RNA: Ribonucleic acid
RNAP: RNA polymerase
RNA-Seq: RNA sequencing
RPKM: Reads per Kilobase of transcript per Million of reads
rRNA: Ribosomal RNA
sRNA: Small RNA
TF: Transcriptional Factor
tmRNA: Transfer messenger RNA
TSS: Transcriptional Start Site
TTS: Transcriptional Termination Site
UTR: Untranslated region
