# Supplementary material for: Global transcriptional landscape and promoter mapping of the gut commensal Bifidobacterium breve UCC2003
Source: BMC Genomics. 2017 Dec 28;18:991. doi: 10.1186/s12864-017-4387-x (PMC5746004; doi:10.1186/s12864-017-4387-x)
Supplement: Supplementary file 1 — Transcribed genes as determined by RNA-Seq and tiling array analyses. A .docx document containing the list of genes detected as expressed in RNA-Seq and Tiling arrays experiments. For each gene the fold-change (FC expressed as level of RNA signal strength vs gDNA baseline) and RKPM values are also indicated. (DOCX 135 kb) [file 12864_2017_4387_MOESM1_ESM.docx]

**Table S1. RNA-seq and tiling arrays expressed genes.**

| **Locus_tag** | **(RNA-seq) RKPM** | **(Tiling arrays) FC** | **PRODUCT** | |
| --- | --- | --- | --- | --- |
| Bbr_0001 | 406 | 409 | Chromosomal replication initiator protein DnaA |  |
| Bbr_0002 | 541 | 542 | DNA polymerase III, beta chain |  |
| Bbr_0005 | 758 | 759 | DNA gyrase subunit B |  |
| Bbr_0006 | 379 | 381 | DNA gyrase subunit A |  |
| Bbr_0007 | 1037 | 1041 | Conserved hypothetical membrane spanning protein |  |
| Bbr_0010 | 251 | 252 | Beta-galactosidase |  |
| Bbr_0011 | 195 | nd | Conserved hypothetical membrane spanning protein, VanZ family |  |
| Bbr_0012 | 319 | 325 | NADP-specific glutamate dehydrogenase |  |
| Bbr_0016 | 2044 | 2045 | Conserved hypothetical protein in DPS family |  |
| Bbr_0026 | 746 | 746 | Permease protein of ABC transporter system for sugars |  |
| Bbr_0027 | 752 | 753 | Permease protein of ABC transporter system for sugars |  |
| Bbr_0033 | 210 | nd | Solute binding protein of ABC transporter system for sugars (MalE family) |  |
| Bbr_0034 | 1807 | 1813 | Conserved hypothetical protein in DPS family |  |
| Bbr_0035 | 181 | nd | Conserved hypothetical membrane spanning protein with duf21 and CBS domains |  |
| Bbr_0037 | 476 | 512 | Carbonic anhydrase |  |
| Bbr_0038 | 3902 | 3904 | Alkyl hydroperoxide reductase C22 protein |  |
| Bbr_0039 | 349 | nd | Thioredoxin reductase/Thioredoxin/Glutaredoxin family protein |  |
| Bbr_0048 | 173 | nd | Conserved hypothetical membrane spanning protein |  |
| Bbr_0052 | 2228 | 2230 | Phosphoenolpyruvate carboxylase |  |
| Bbr_0053 | 165 | nd | Conserved hypothetical membrane spanning protein with DUF1212 domain |  |
| Bbr_0057 | 652 | 659 | Tryptophanyl-tRNA synthetase |  |
| Bbr_0058 | 864 | 895 | Conserved hypothetical protein |  |
| Bbr_0059 | 151 | nd | Conserved hypothetical protein |  |
| Bbr_0060 | 1949 | 1950 | Glycogen or amylose phosphorylase |  |
| Bbr_0061 | 1475 | 1479 | Conserved hypothetical protein |  |
| Bbr_0063 | 1134 | 1144 | Conserved hypothetical membrane spanning protein in uncharacterized protein family (UPF0233) |  |
| Bbr_0064 | 343 | 347 | Conserved hypothetical secreted protein with DUF881 domain |  |
| Bbr_0065 | 251 | 252 | belongs to sortase family |  |
| Bbr_0066 | 273 | 275 | Para-aminobenzoate synthase glutamine amidotransferase component II |  |
| Bbr_0067 | 574 | 577 | Serine/threonine protein kinase with PASTA domain |  |
| Bbr_0068 | 418 | 421 | Serine/threonine protein kinase |  |
| Bbr_0069 | 400 | 404 | Penicillin-binding protein |  |
| Bbr_0070 | 486 | 494 | Cell division protein FtsW |  |
| Bbr_0071 | 486 | 487 | Protein phosphatase 2C |  |
| Bbr_0072 | 528 | 535 | Conserved hypothetical secreted protein with FHA domain |  |
| Bbr_0073 | 1261 | 1262 | Conserved hypothetical protein with FHA domain |  |
| Bbr_0074 | 184 | nd | Xaa-Pro dipeptidyl-peptidase |  |
| Bbr_0075 | 160 | nd | Lysophospholipase L2 |  |
| Bbr_0076 | 1153 | nd | Hsp20-family heat shock chaperone |  |
| Bbr_0081 | 325 | 328 | Peptide methionine sulfoxide reductase msrA/msrB |  |
| Bbr_0082 | 388 | 399 | Antibiotic resistance protein |  |
| Bbr_0086 | 288 | nd | Conserved hypothetical protein with DUF74 domain |  |
| Bbr_0089 | 638 | 641 | trypsin-like serine protease |  |
| Bbr_0090 | 224 | 225 | Lead, cadmium, zinc and mercury transporting ATPase |  |
| Bbr_0091 | 499 | 500 | Ferredoxin--NADP reductase |  |
| Bbr_0092 | 468 | 468 | Endopeptidase htpX, peptidase family M48 |  |
| Bbr_0094 | 398 | nd | Fructose-bisphosphate aldolase |  |
| Bbr_0095 | 582 | 582 | Adenylosuccinate synthetase |  |
| Bbr_0096 | 224 | 224 | Chloride channel protein |  |
| Bbr_0097 | 329 | nd | CrcB family protein |  |
| Bbr_0098 | 186 | nd | CrcB family protein |  |
| Bbr_0104 | 503 | 504 | Ketol-acid reductoisomerase/2-dehydropantoate 2-reductase |  |
| Bbr_0105 | 174 | 175 | Cellodextrin transport system transcriptional regulator |  |
| Bbr_0106 | 297 | nd | Cellodextrin binding protein |  |
| Bbr_0116 | 288 | 290 | 4-alpha-glucanotransferase |  |
| Bbr_0118 | 854 | 856 | Maltose/maltodextrin-binding protein |  |
| Bbr_0122 | 191 | nd | Transcriptional regulator, LacI family |  |
| Bbr_0124 | 817 | 818 | Chaperone protein dnaK |  |
| Bbr_0125 | 694 | 697 | GrpE protein |  |
| Bbr_0126 | 664 | 664 | Chaperone protein dnaJ |  |
| Bbr_0127 | 274 | 275 | Heat shock regulatory protein hspR |  |
| Bbr_0130 | 407 | 408 | Haloacid dehalogenase-like hydrolase (HAD superfamily) |  |
| Bbr_0131 | 351 | 352 | DedA family protein |  |
| Bbr_0141 | 154 | nd | DNA polymerase III subunit gamma/tau |  |
| Bbr_0147 | 331 | 334 | Aspartokinase |  |
| Bbr_0148 | 274 | 279 | Aspartokinase |  |
| Bbr_0149 | 245 | 247 | Aspartate-semialdehyde dehydrogenase |  |
| Bbr_0150 | 240 | 240 | Conserved hypothetical protein |  |
| Bbr_0151 | 613 | nd | Transposase |  |
| Bbr_0152 | 527 | nd | Transposase |  |
| Bbr_0173 | 191 | nd | Transcriptional regulator, GntR family |  |
| Bbr_0175 | 378 | 381 | 2-isopropylmalate synthase |  |
| Bbr_0176 | 253 | 268 | Penicillin binding protein |  |
| Bbr_0178 | 273 | 274 | DNA topoisomerase I |  |
| Bbr_0179 | 329 | 337 | Thymidylate kinase |  |
| Bbr_0180 | 198 | 233 | DNA polymerase III, delta' subunit |  |
| Bbr_0181 | 395 | 423 | Transcriptional regulator, LacI family |  |
| Bbr_0182 | 3537 | 3537 | Phosphocarrier protein HPr |  |
| Bbr_0183 | 734 | 735 | Phosphoenolpyruvate-protein phosphotransferase |  |
| Bbr_0186 | 739 | 743 | Conserved hypothetical protein |  |
| Bbr_0187 | 535 | 537 | Dipeptidase A, family C69 |  |
| Bbr_0188 | 544 | 546 | Formate--tetrahydrofolate ligase |  |
| Bbr_0189 | 1180 | 1193 | Conserved hypothetical protein |  |
| Bbr_0190 | 343 | 354 | Conserved hypothetical membrane spanning protein with GtrA-like domain |  |
| Bbr_0191 | 216 | 244 | Conserved hypothetical membrane spanning protein |  |
| Bbr_0193 | 329 | 333 | Conserved hypothetical protein |  |
| Bbr_0196 | 489 | 490 | Glutamyl-tRNA synthetase |  |
| Bbr_0199 | 586 | 588 | Transcriptional regulator, MarR family |  |
| Bbr_0203 | 808 | 809 | Conserved hypothetical membrane spanning protein, possibly efflux system |  |
| Bbr_0204 | 835 | 839 | Multi-domain protein possibly involved in fatty acids or polyketide biosynthesis |  |
| Bbr_0205 | 542 | 544 | Multi-domain protein possibly involved in fatty acids or polyketide biosynthesis |  |
| Bbr_0208 | 464 | 509 | Transcriptional regulator, TetR family |  |
| Bbr_0209 | 168 | 269 | Phosphopantetheinyl transferase |  |
| Bbr_0213 | 220 | 225 | Hypothetical protein |  |
| Bbr_0215 | 372 | 387 | Conserved hypothetical protein |  |
| Bbr_0216 | 297 | 299 | Modification methylase |  |
| Bbr_0221 | 177 | 178 | Conserved hypothetical membrane spanning protein with iron permease FTR1 family domain |  |
| Bbr_0222 | 854 | 858 | Conserved hypothetical secreted protein, probably involved in iron uptake |  |
| Bbr_0225 | 157 | nd | Permease protein of ABC transporter system |  |
| Bbr_0226 | 171 | nd | ATP-binding protein of ABC transporter system |  |
| Bbr_0227 | 331 | nd | Conserved hypothetical protein |  |
| Bbr_0231 | 366 | 367 | Conserved hypothetical membrane spanning protein with DUF1113 domain |  |
| Bbr_0232 | 888 | 908 | 6-phosphogluconate dehydrogenase |  |
| Bbr_0233 | 174 | 246 | 6-phosphogluconolactonase |  |
| Bbr_0234 | 454 | 460 | glucose-6-phosphate dehydrogenase subunit |  |
| Bbr_0235 | 399 | 400 | Glucose-6-phosphate 1-dehydrogenase |  |
| Bbr_0237 | 287 | nd | Dipeptidase A |  |
| Bbr_0238 | 445 | 493 | Glycosyltransferase |  |
| Bbr_0239 | 265 | 270 | Transcriptional regulator, TetR family |  |
| Bbr_0240 | 252 | 256 | Cell division protein ftsY |  |
| Bbr_0241 | 504 | 507 | Ammonium transporter |  |
| Bbr_0242 | 569 | 570 | Nitrogen regulatory protein P-II |  |
| Bbr_0245 | 218 | nd | Replicative DNA helicase |  |
| Bbr_0246 | 235 | nd | UDP-N-acetylmuramoylalanyl-D-glutamate--2, 6-diaminopimelate ligase |  |
| Bbr_0247 | 207 | nd | CobB/CobQ-like glutamine amidotransferase domain protein |  |
| Bbr_0248 | 4361 | 4383 | Conserved hypothetical protein |  |
| Bbr_0249 | 1496 | 1500 | ABC1 family protein kinase |  |
| Bbr_0254 | 9066 | 9068 | SSU ribosomal protein S6P |  |
| Bbr_0255 | 8088 | 8090 | Single-strand DNA binding protein |  |
| Bbr_0256 | 11105 | 11108 | SSU ribosomal protein S18P |  |
| Bbr_0257 | 6701 | 6703 | LSU ribosomal protein L9P |  |
| Bbr_0263 | 2789 | 2793 | MFS transporter |  |
| Bbr_0264 | 2114 | 2114 | Hypothetical protein |  |
| Bbr_0265 | 2347 | 2349 | Hypothetical protein |  |
| Bbr_0267 | 1096 | 1107 | Glycerol uptake facilitator protein |  |
| Bbr_0271 | 333 | nd | Orotate phosphoribosyltransferase |  |
| Bbr_0272 | 271 | nd | 23S rRNA methyltransferase |  |
| Bbr_0276 | 917 | 923 | Aspartyl/glutamyl-tRNA(Asn/Gln) amidotransferase subunit C |  |
| Bbr_0277 | 710 | 711 | Aspartyl/glutamyl-tRNA(Asn/Gln) amidotransferase subunit A |  |
| Bbr_0278 | 1247 | 1249 | Glutamyl-tRNA(Gln) amidotransferase subunit B |  |
| Bbr_0279 | 431 | 432 | Acetyltransferase (GNAT) family |  |
| Bbr_0280 | 617 | 617 | Conserved hypothetical protein |  |
| Bbr_0281 | 1435 | 1435 | Conserved hypothetical protein, possibly FAD-containing oxidoreductase |  |
| Bbr_0282 | 734 | 738 | Transcription termination factor rho |  |
| Bbr_0285 | 220 | nd | Beta-galactosidase |  |
| Bbr_0287 | 586 | 586 | Chorismate mutase |  |
| Bbr_0288 | 257 | 271 | Conserved hypothetical secreted protein |  |
| Bbr_0289 | 541 | 561 | Valyl-tRNA synthetase |  |
| Bbr_0291 | 199 | 201 | Endonuclease III |  |
| Bbr_0292 | 608 | 609 | Transcriptional regulatory protein glnR |  |
| Bbr_0293 | 604 | 607 | Conserved hypothetical membrane spanning protein |  |
| Bbr_0294 | 470 | 472 | Conserved hypothetical membrane spanning protein |  |
| Bbr_0295 | 3531 | 3533 | Inorganic pyrophosphatase |  |
| Bbr_0296 | 252 | 253 | Alpha-amylase family protein |  |
| Bbr_0300 | 286 | nd | Hypothetical secreted protein |  |
| Bbr_0307 | 155 | nd | Conserved hypothetical membrane spanning protein with DUF624 domain |  |
| Bbr_0319 | 1191 | nd | Phage transcriptional regulator |  |
| Bbr_0320 | 1211 | nd | Hypothetical protein |  |
| Bbr_0322 | 194 | nd | Homoserine O-succinyltransferase |  |
| Bbr_0323 | 2989 | 2990 | ATP synthase A chain |  |
| Bbr_0324 | 3534 | 3536 | ATP synthase C chain |  |
| Bbr_0325 | 2669 | 2671 | ATP synthase B chain |  |
| Bbr_0326 | 3190 | 3192 | ATP synthase delta chain |  |
| Bbr_0327 | 3883 | 3886 | ATP synthase alpha chain |  |
| Bbr_0328 | 4514 | 4516 | ATP synthase gamma chain |  |
| Bbr_0329 | 3225 | 3227 | ATP synthase beta chain |  |
| Bbr_0330 | 2894 | 2898 | ATP synthase epsilon chain |  |
| Bbr_0332 | 601 | 620 | Peptidyl-prolyl cis-trans isomerase |  |
| Bbr_0333 | 370 | 370 | Conserved hypothetical membrane spanning protein |  |
| Bbr_0334 | 490 | 492 | Conserved hypothetical secreted protein |  |
| Bbr_0335 | 348 | 387 | thioredoxin-like protein |  |
| Bbr_0336 | 345 | nd | Conserved hypothetical protein with CYTH domain, possible adenylate cyclase |  |
| Bbr_0339 | 279 | 280 | 1-acyl-sn-glycerol-3-phosphate acyltransferase |  |
| Bbr_0340 | 307 | 308 | Glycerol-3-phosphate dehydrogenase [NAD(P)+] |  |
| Bbr_0341 | 255 | 255 | D-alanine--D-alanine ligase |  |
| Bbr_0347 | 158 | nd | Conserved hypothetical membrane spanning protein with mechanosensitive ion channel |  |
| Bbr_0351 | 183 | nd | Transcriptional regulatory protein, LacI family |  |
| Bbr_0358 | 164 | nd | Conserved hypothetical protein |  |
| Bbr_0369 | 181 | nd | Conserved hypothetical membrane spanning protein |  |
| Bbr_0370 | 5845 | 5850 | SSU ribosomal protein S15P |  |
| Bbr_0371 | 741 | 742 | Polyribonucleotide nucleotidyltransferase |  |
| Bbr_0374 | 162 | nd | LemA protein |  |
| Bbr_0377 | 326 | 329 | Oxidoreductase, aldo/keto reductase family |  |
| Bbr_0379 | 353 | 386 | 16S rRNA processing protein rimM |  |
| Bbr_0380 | 1704 | 1705 | RNA binding protein |  |
| Bbr_0381 | 1743 | 1744 | SSU ribosomal protein S16P |  |
| Bbr_0382 | 184 | nd | Conserved hypothetical membrane spanning protein |  |
| Bbr_0383 | 336 | nd | Signal recognition particle, subunit FFH/SRP54 |  |
| Bbr_0384 | 426 | 448 | Conserved hypothetical membrane spanning protein in cation efflux family |  |
| Bbr_0385 | 237 | 241 | Cysteinyl-tRNA synthetase |  |
| Bbr_0386 | 416 | 417 | Glutamine amidotrasnferase Class I |  |
| Bbr_0387 | 249 | 252 | ATP-binding protein of ABC transporter system |  |
| Bbr_0389 | 182 | nd | Hypothetical protein |  |
| Bbr_0390 | 229 | nd | Acetolactate synthase small subunit |  |
| Bbr_0391 | 176 | nd | Acetolactate synthase large subunit |  |
| Bbr_0392 | 236 | nd | Ribonuclease III |  |
| Bbr_0393 | 8215 | 8218 | LSU ribosomal protein L32P |  |
| Bbr_0394 | 375 | nd | Conserved hypothetical protein |  |
| Bbr_0395 | 511 | nd | Conserved hypothetical protein |  |
| Bbr_0396 | 510 | nd | Phosphopantetheine adenylyltransferase |  |
| Bbr_0397 | 576 | 590 | Conserved hypothetical protein |  |
| Bbr_0401 | 152 | 152 | Nicotinate phosphoribosyltransferase |  |
| Bbr_0402 | 426 | 431 | Ribonuclease PH |  |
| Bbr_0403 | 530 | 533 | Conserved hypothetical protein with HAM1 family domain |  |
| Bbr_0405 | 2867 | 2869 | Glucose-6-phosphate isomerase |  |
| Bbr_0407 | 247 | nd | Permease protein of ABC transporter system |  |
| Bbr_0408 | 6135 | 6137 | LSU ribosomal protein L19P |  |
| Bbr_0409 | 381 | 384 | Signal peptidase I |  |
| Bbr_0410 | 180 | 183 | Ribonuclease HII |  |
| Bbr_0413 | 305 | nd | Long-chain-fatty-acid--CoA ligase |  |
| Bbr_0428 | 155 | 173 | Conserved hypothetical membrane spanning protein |  |
| Bbr_0429 | 405 | 413 | Conserved hypothetical protein |  |
| Bbr_0430 | 632 | 645 | Undecaprenyl-phosphate galactosephosphotransferase |  |
| Bbr_0431 | 380 | 381 | Protein tyrosine phosphatase |  |
| Bbr_0434 | 216 | 219 | Oligosaccharide repeat unit transporter |  |
| Bbr_0435 | 479 | 485 | Beta-1,6-N-acetylglucosaminyltransferase |  |
| Bbr_0436 | 450 | 461 | Hypothetical membrane spanning protein |  |
| Bbr_0437 | 717 | 729 | Acetyltransferase |  |
| Bbr_0438 | 475 | 476 | Glycosyltransferase |  |
| Bbr_0439 | 548 | 549 | Capsular polysaccharide biosynthesis protein |  |
| Bbr_0441 | 387 | 389 | Capsular polysaccharide biosynthesis protein |  |
| Bbr_0445 | 238 | nd | Glycosyltransferase |  |
| Bbr_0446 | 294 | nd | Acetyltransferase (cell wall biosynthesis) |  |
| Bbr_0447 | 236 | nd | Conserved hypothetical protein |  |
| Bbr_0448 | 240 | nd | Glycosyltransferase |  |
| Bbr_0461 | 677 | nd | Hypothetical protein |  |
| Bbr_0465 | 150 | nd | Hypothetical protein |  |
| Bbr_0466 | 766 | 774 | Hypothetical protein |  |
| Bbr_0467 | 853 | 854 | Conserved hypothetical protein with a helix-turn-helix motif |  |
| Bbr_0468 | 455 | 460 | Hypothetical protein |  |
| Bbr_0472 | 161 | nd | Conserved hypothetical membrane spanning protein |  |
| Bbr_0473 | 700 | 702 | Conserved hypothetical protein |  |
| Bbr_0474 | 681 | 682 | Chain length regulator (capsular polysaccharide biosynthesis) / Tyrosine-protein kinase (capsular polysaccharide biosynthesis) |  |
| Bbr_0475 | 379 | 380 | Conserved hypothetical protein |  |
| Bbr_0476 | 440 | 484 | Conserved hypothetical secreted protein with G5 domain |  |
| Bbr_0477 | 1346 | 1349 | Thioredoxin |  |
| Bbr_0478 | 315 | 320 | Conserved hypothetical protein |  |
| Bbr_0479 | 237 | 243 | Hydrolase |  |
| Bbr_0481 | 445 | nd | Glycine cleavage system H protein |  |
| Bbr_0482 | 276 | nd | Conserved hypothetical protein |  |
| Bbr_0484 | 232 | nd | 3-isopropylmalate dehydrogenase |  |
| Bbr_0486 | 258 | 259 | Transcription regulator, crp family |  |
| Bbr_0487 | 681 | 683 | Penicillin-binding protein |  |
| Bbr_0488 | 188 | nd | NADH-dependent flavin oxidoreductase |  |
| Bbr_0490 | 454 | 455 | Transcriptional regulator, DeoR family |  |
| Bbr_0491 | 708 | 708 | Galactose-1-phosphate uridylyltransferase |  |
| Bbr_0492 | 406 | 406 | Galactokinase |  |
| Bbr_0493 | 1024 | 1024 | Conserved hypothetical protein with ACT domain |  |
| Bbr_0494 | 476 | 476 | Conserved hypothetical protein |  |
| Bbr_0495 | 290 | 293 | RNA methyltransferase |  |
| Bbr_0497 | 228 | nd | Conserved hypothetical protein |  |
| Bbr_0498 | 638 | 639 | DNA-directed RNA polymerase beta chain |  |
| Bbr_0499 | 1226 | 1226 | DNA-directed RNA polymerase beta' chain |  |
| Bbr_0507 | 249 | nd | Conserved hypothetical membrane spanning protein, member of major facilitator superfamily (MFS) |  |
| Bbr_0508 | 741 | nd | Dihydrodipicolinate reductase |  |
| Bbr_0509 | 1064 | nd | Dihydrodipicolinate synthase |  |
| Bbr_0510 | 715 | nd | Metal-dependent hydrolase |  |
| Bbr_0511 | 683 | nd | Alanine aminopeptidase |  |
| Bbr_0512 | 395 | nd | Phosphoglucosamine mutase |  |
| Bbr_0513 | 456 | nd | Peptide deformylase |  |
| Bbr_0514 | 288 | nd | Conserved hypothetical protein |  |
| Bbr_0515 | 607 | nd | Bacterial Peptide Chain Release Factor 2 (RF-2) |  |
| Bbr_0516 | 579 | nd | Cell division ATP-binding protein ftsE |  |
| Bbr_0517 | 619 | nd | Cell division protein ftsX |  |
| Bbr_0518 | 535 | nd | Conserved hypothetical secreted protein with CHAP domain |  |
| Bbr_0519 | 337 | nd | SsrA-binding protein |  |
| Bbr_0520 | 254 | nd | Solute-binding protein of ABC transporter system |  |
| Bbr_0521 | 1085 | nd | Solute binding protein of ABC transporter system |  |
| Bbr_0522 | 450 | nd | Permease protein of ABC transporter system |  |
| Bbr_0523 | 275 | nd | ATP-binding protein of ABC transporter system |  |
| Bbr_0524 | 307 | nd | Glucosamine--fructose-6-phosphate aminotransferase |  |
| Bbr_0527 | 1929 | 1929 | Permease protein of ABC transporter system for sugars |  |
| Bbr_0528 | 1321 | 1323 | Permease protein of ABC transporter system for sugars |  |
| Bbr_0529 | 640 | 640 | Beta-galactosidase |  |
| Bbr_0530 | 1372 | 1374 | Solute-binding protein of ABC transporter system for sugars |  |
| Bbr_0533 | 252 | 253 | Solute-binding protein of ABC transporter system for peptides |  |
| Bbr_0541 | 629 | 631 | Autoinducer-2 production protein luxS |  |
| Bbr_0542 | 164 | nd | Serine O-acetyltransferase |  |
| Bbr_0543 | 187 | 208 | Amino acid permease |  |
| Bbr_0545 | 191 | nd | Deoxyguanosinetriphosphate triphosphohydrolase |  |
| Bbr_0549 | 388 | nd | Pyridoxine biosynthesis protein |  |
| Bbr_0550 | 391 | nd | Pyridoxine biosynthesis amidotransferase |  |
| Bbr_0551 | 214 | nd | Multiple substrate aminotransferase (MsaT) containing domain and a regulatory domain of the GntR family |  |
| Bbr_0553 | 2837 | 2862 | Conserved hypothetical protein |  |
| Bbr_0557 | 159 | nd | Solute-binding protein of ABC transporter system for sugars |  |
| Bbr_0561 | 157 | nd | Peptide methionine sulfoxide reductase msrA/msrB |  |
| Bbr_0563 | 160 | 161 | Conserved hypothetical membrane spanning protein, possibly ABC-type permease |  |
| Bbr_0567 | 392 | 393 | Phosphoribosylaminoimidazole carboxylase carboxyltransferase subunit |  |
| Bbr_0568 | 310 | 310 | Phosphoribosylaminoimidazole carboxylase NCAIR mutase subunit |  |
| Bbr_0569 | 567 | 567 | Ferric uptake regulation protein |  |
| Bbr_0573 | 172 | nd | Ferric uptake regulation protein |  |
| Bbr_0579 | 1687 | nd | Solute binding protein of ABC transporter system |  |
| Bbr_0580 | 904 | 904 | Phosphoglycerol transferase, contains sulfatase domain |  |
| Bbr_0581 | 230 | 235 | Aldehyde dehydrogenase (NAD(P)+) |  |
| Bbr_0582 | 488 | 492 | Phosphoribosylamine--glycine ligase |  |
| Bbr_0583 | 636 | 638 | Phosphoribosylformylglycinamidine cyclo-ligase |  |
| Bbr_0584 | 499 | 500 | Amidophosphoribosyltransferase |  |
| Bbr_0597 | 343 | 344 | Phosphoribosylformylglycinamidine synthase |  |
| Bbr_0598 | 1392 | 1393 | Phosphoribosylamidoimidazole-succinocarboxamide synthase |  |
| Bbr_0599 | 196 | 197 | Phosphoribosylglycinamide formyltransferase |  |
| Bbr_0601 | 247 | 275 | Glycosyltransferase |  |
| Bbr_0602 | 165 | nd | Low specificity-threonine aldolase |  |
| Bbr_0604 | 4718 | 4719 | SSU ribosomal protein S12P |  |
| Bbr_0605 | 3748 | 3750 | SSU ribosomal protein S7P |  |
| Bbr_0606 | 3929 | 3931 | Protein Translation Elongation Factor G (EF-G) |  |
| Bbr_0607 | 21542 | 21551 | Protein Translation Elongation Factor Tu (EF-TU) |  |
| Bbr_0613 | 196 | nd | CrcB family protein |  |
| Bbr_0631 | 2970 | 2972 | Protein Translation Elongation Factor P (EF-P) |  |
| Bbr_0632 | 2027 | 2030 | N utilization substance protein B |  |
| Bbr_0633 | 613 | 613 | Carbamoyl-phosphate synthase small chain |  |
| Bbr_0634 | 785 | 786 | Carbamoyl-phosphate synthase large chain |  |
| Bbr_0635 | 694 | 696 | Orotidine 5'-phosphate decarboxylase |  |
| Bbr_0636 | 399 | 408 | Guanylate kinase |  |
| Bbr_0641 | 270 | nd | Cystathionine beta-lyase |  |
| Bbr_0642 | 1161 | 1168 | Glutaredoxin |  |
| Bbr_0646 | 195 | 195 | Glutamate racemase |  |
| Bbr_0647 | 238 | 246 | Diaminopimelate epimerase |  |
| Bbr_0650 | 771 | 824 | Conserved hypothetical protein |  |
| Bbr_0651 | 333 | 337 | Conserved hypothetical secreted protein |  |
| Bbr_0653 | 441 | 450 | Conserved hypothetical protein |  |
| Bbr_0654 | 202 | 204 | Conserved hypothetical protein, contains Lon protease motif |  |
| Bbr_0655 | 685 | 685 | Conserved hypothetical protein |  |
| Bbr_0656 | 372 | 373 | Conserved hypothetical protein |  |
| Bbr_0657 | 442 | 442 | 1-deoxy-D-xylulose 5-phosphate reductoisomerase |  |
| Bbr_0658 | 384 | 386 | 1-hydroxy-2-methyl-2-(E)-butenyl 4-diphosphate synthase |  |
| Bbr_0659 | 201 | 201 | Conserved hypothetical membrane spanning protein |  |
| Bbr_0661 | 241 | 272 | Undecaprenyl pyrophosphate synthetase |  |
| Bbr_0662 | 208 | nd | Permease protein of ABC transporter system |  |
| Bbr_0663 | 2053 | 2055 | Solute binding protein of ABC transporter system |  |
| Bbr_0665 | 195 | nd | ATP-binding protein of ABC transporter system |  |
| Bbr_0667 | 206 | 207 | Conserved hypothetical protein |  |
| Bbr_0669 | 177 | 181 | ATP-binding Mrp-like protein |  |
| Bbr_0670 | 1865 | 1866 | Glutamine synthetase |  |
| Bbr_0671 | 891 | 893 | Conserved hypothetical membrane spanning protein |  |
| Bbr_0672 | 226 | 226 | Dihydrolipoamide dehydrogenase |  |
| Bbr_0673 | 910 | 912 | Conserved hypothetical membrane spanning protein |  |
| Bbr_0674 | 221 | 224 | Peptidase family M20A protein |  |
| Bbr_0675 | 194 | 194 | Permease protein of ABC transporter system, probably thiamine metabolism |  |
| Bbr_0678 | 240 | 240 | rRNA methylase |  |
| Bbr_0679 | 679 | 680 | Phenylalanyl-tRNA synthetase alpha chain |  |
| Bbr_0680 | 656 | 657 | Phenylalanyl-tRNA synthetase beta chain |  |
| Bbr_0681 | 702 | 703 | Conserved hypothetical membrane spanning protein |  |
| Bbr_0682 | 321 | 323 | N-acetyl-gamma-glutamyl-phosphate reductase |  |
| Bbr_0683 | 264 | 266 | Glutamate N-acetyltransferase/Amino-acid acetyltransferase |  |
| Bbr_0684 | 265 | 267 | Acetylglutamate kinase |  |
| Bbr_0685 | 224 | 225 | Acetylornithine aminotransferase |  |
| Bbr_0686 | 270 | 271 | Ornithine carbamoyltransferase |  |
| Bbr_0687 | 217 | 223 | Arginine repressor, argR |  |
| Bbr_0688 | 806 | 812 | Argininosuccinate synthase |  |
| Bbr_0692 | 304 | 306 | Argininosuccinate lyase |  |
| Bbr_0693 | 287 | nd | ThiS protein |  |
| Bbr_0694 | 160 | nd | Thiazole biosynthesis protein thiG |  |
| Bbr_0697 | 468 | nd | Hydrolase (HAD superfamily) |  |
| Bbr_0700 | 673 | 674 | Tyrosyl-tRNA synthetase |  |
| Bbr_0701 | 1255 | 1255 | Conserved hypothetical protein |  |
| Bbr_0702 | 811 | 814 | Haloacid dehalogenase-like hydrolase (HAD superfamily) |  |
| Bbr_0703 | 298 | 307 | Hemolysin-like protein with S4 and FtsJ-like methyltransferase domains |  |
| Bbr_0711 | 169 | nd | Transcriptional regulator, GntR family |  |
| Bbr_0715 | 246 | 251 | Threonine synthase |  |
| Bbr_0716 | 385 | 386 | Gamma-glutamyl phosphate reductase |  |
| Bbr_0717 | 464 | 466 | Conserved hypothetical protein |  |
| Bbr_0718 | 383 | 384 | Nicotinate-nucleotide adenylyltransferase |  |
| Bbr_0720 | 509 | 511 | Conserved hypothetical membrane spanning protein in uncharacterized protein family (UPF0182) |  |
| Bbr_0721 | 156 | 157 | Phosphinothricin N-acetyltransferase |  |
| Bbr_0722 | 252 | 253 | Peptidyl-tRNA hydrolase |  |
| Bbr_0723 | 210 | 211 | Transcription-repair coupling factor |  |
| Bbr_0724 | 294 | 296 | Oxidoreductase, aldo-keto reductase family |  |
| Bbr_0725 | 2663 | 2664 | Enolase |  |
| Bbr_0726 | 579 | 582 | Conserved hypothetical membrane spanning protein with septum formation initiator domain |  |
| Bbr_0727 | 426 | 429 | Conserved hypothetical protein with DUF501 domain |  |
| Bbr_0728 | 301 | 302 | phosphatase |  |
| Bbr_0730 | 183 | 193 | L-serine dehydratase |  |
| Bbr_0731 | 1259 | 1260 | Peptidyl-prolyl cis-trans isomerase |  |
| Bbr_0732 | 943 | 943 | Transcription elongation factor greA |  |
| Bbr_0734 | 197 | 201 | Sensory transduction protein kinase |  |
| Bbr_0735 | 302 | 302 | Cell division transcription factor WhmD |  |
| Bbr_0737 | 166 | nd | Transcriptional regulator |  |
| Bbr_0738 | 425 | 425 | Transcription factor WhiB |  |
| Bbr_0739 | 222 | 223 | Conserved hypothetical membrane spanning protein |  |
| Bbr_0741 | 171 | nd | Conserved hypothetical protein |  |
| Bbr_0744 | 300 | 306 | Sensory transduction protein kinase |  |
| Bbr_0745 | 613 | 617 | Two-component response regulator |  |
| Bbr_0746 | 348 | 364 | 1,4-alpha-glucan branching enzyme |  |
| Bbr_0747 | 353 | 354 | CarD-like transcriptional regulator |  |
| Bbr_0748 | 276 | 280 | 2-C-methyl-D-erythritol 2,4-cyclodiphosphate synthase |  |
| Bbr_0750 | 214 | nd | ATP-binding protein of ABC transporter system for metals |  |
| Bbr_0752 | 214 | nd | Bifunctional methylenetetrahydrofolate dehydrogenase/cyclohydrolase |  |
| Bbr_0753 | 6892 | 6894 | SSU ribosomal protein S1P |  |
| Bbr_0754 | 156 | nd | Dephospho-CoA kinase |  |
| Bbr_0756 | 446 | 448 | Tellurium resistance protein TerC-like integral membrane protein |  |
| Bbr_0757 | 2854 | 2861 | Pyruvate kinase |  |
| Bbr_0758 | 1468 | 1497 | ADP-ribose pyrophosphatase |  |
| Bbr_0759 | 1169 | nd | Putative response regulator with RNA-binding domain |  |
| Bbr_0760 | 320 | nd | DNA polymerase I |  |
| Bbr_0761 | 160 | nd | NIF3-related protein |  |
| Bbr_0762 | 289 | nd | Phosphohydrolase (MutT/nudix family protein) |  |
| Bbr_0763 | 195 | nd | Glycogen operon protein glgX |  |
| Bbr_0764 | 200 | nd | Conserved hypothetical protein |  |
| Bbr_0771 | 1647 | 1649 | Acetate kinase |  |
| Bbr_0772 | 207 | 208 | Phosphate acetyltransferase |  |
| Bbr_0773 | 445 | 446 | Ribose-phosphate pyrophosphokinase |  |
| Bbr_0774 | 232 | nd | Acyltransferase family protein |  |
| Bbr_0775 | 451 | nd | GMP synthase |  |
| Bbr_0776 | 10036 | 10039 | Xylulose-5-phosphate/Fructose-6-phosphate phosphoketolase |  |
| Bbr_0777 | 353 | 354 | UDP-N-acetylglucosamine pyrophosphorylase |  |
| Bbr_0778 | 479 | 479 | iojap protein family |  |
| Bbr_0779 | 603 | 603 | Phosphoglycerate mutase family protein |  |
| Bbr_0781 | 412 | 414 | Solute-binding protein of ABC transporter system |  |
| Bbr_0785 | 223 | 224 | Glutamine-dependent NAD(+) synthetase |  |
| Bbr_0786 | 401 | 402 | Conserved hypothetical protein |  |
| Bbr_0787 | 1209 | 1211 | Formate acetyltransferase |  |
| Bbr_0788 | 166 | nd | Pyruvate formate-lyase activating enzyme |  |
| Bbr_0789 | 351 | 357 | Conserved hypothetical protein |  |
| Bbr_0790 | 268 | 270 | Ribonuclease D |  |
| Bbr_0791 | 2713 | 2715 | Trigger factor, ppiase |  |
| Bbr_0794 | 1500 | 1502 | ATP-dependent Clp protease proteolytic subunit |  |
| Bbr_0795 | 1643 | 1644 | ATP-dependent Clp protease proteolytic subunit |  |
| Bbr_0796 | 152 | nd | ATP-dependent Clp protease ATP-binding subunit clpX |  |
| Bbr_0797 | 315 | nd | Cystathionine beta-lyase |  |
| Bbr_0798 | 191 | nd | Na+/H+ antiporter nhaA |  |
| Bbr_0801 | 258 | 261 | Succinate dehydrogenase iron-sulfur protein |  |
| Bbr_0802 | 215 | 218 | Succinate dehydrogenase flavoprotein subunit |  |
| Bbr_0807 | 603 | nd | O-acetylhomoserine aminocarboxypropyltransferase |  |
| Bbr_0808 | 626 | nd | ATP-binding protein of ABC transporter system |  |
| Bbr_0809 | 202 | nd | Permease protein of ABC transporter system |  |
| Bbr_0810 | 302 | nd | Two-component response regulator |  |
| Bbr_0812 | 179 | nd | Proline iminopeptidase |  |
| Bbr_0813 | 211 | nd | Pyrroline-5-carboxylate reductase |  |
| Bbr_0814 | 227 | 268 | Glutamine-binding protein glnH |  |
| Bbr_0818 | 468 | 484 | GTP-binding protein, probable translation factor |  |
| Bbr_0819 | 371 | 456 | Transcriptional regulator, AraC family |  |
| Bbr_0820 | 718 | 720 | Conserved hypothetical protein |  |
| Bbr_0827 | 252 | nd | Conserved hypothetical protein |  |
| Bbr_0836 | 342 | nd | Conserved hypothetical protein |  |
| Bbr_0844 | 218 | nd | hypothetical protein, contains helix-turn-helix domain |  |
| Bbr_0853 | 204 | nd | Arylsulfatase regulator (Fe-S oxidoreductase) |  |
| Bbr_0857 | 327 | 329 | Glyoxalase family protein |  |
| Bbr_0859 | 157 | 157 | Macrolide-efflux protein, MSF member |  |
| Bbr_0860 | 306 | 308 | Conserved hypothetical protein |  |
| Bbr_0862 | 716 | 717 | Uracil/xanthine permease |  |
| Bbr_0863 | 497 | 499 | ATP-dependent RNA helicase |  |
| Bbr_0870 | 167 | nd | ATP-binding protein of ABC transporter system |  |
| Bbr_0882 | 175 | 181 | Xanthine permease |  |
| Bbr_0883 | 347 | 348 | Xanthine phosphoribosyltransferase |  |
| Bbr_0884 | 202 | nd | ATP-dependent DNA helicase pcrA |  |
| Bbr_0885 | 285 | nd | Conserved hypothetical membrane spanning protein |  |
| Bbr_0888 | 7541 | 7544 | SSU ribosomal protein S4P |  |
| Bbr_0892 | 206 | 262 | Conserved hypothetical protein, putative transglutaminase, possible cysteine protease |  |
| Bbr_0893 | 363 | 434 | Hypothetical protein |  |
| Bbr_0894 | 424 | 460 | Hypothetical protein |  |
| Bbr_0895 | 338 | 342 | Phosphoglycerate mutase family protein |  |
| Bbr_0896 | 1570 | 1571 | Conserved hypothetical, possibly secreted protein |  |
| Bbr_0897 | 2496 | 2496 | Conserved hypothetical, possibly secreted protein |  |
| Bbr_0898 | 712 | 713 | Alanyl-tRNA synthetase |  |
| Bbr_0899 | 998 | 999 | Endonuclease involved in recombination |  |
| Bbr_0900 | 472 | 475 | Conserved hypothetical protein with aminodeoxychorismate lyase domain |  |
| Bbr_0902 | 159 | 159 | Chorismate synthase |  |
| Bbr_0905 | 702 | 705 | CTP synthase |  |
| Bbr_0906 | 370 | 371 | Conserved hypothetical protein with ErfK/YbiS/YcfS/YnhG domain |  |
| Bbr_0907 | 685 | 687 | FeS assembly protein SufB |  |
| Bbr_0908 | 693 | 693 | ATP-binding protein of ABC transporter system |  |
| Bbr_0909 | 749 | 751 | FeS assembly protein SufC |  |
| Bbr_0910 | 465 | 465 | Cysteine desulfurase/Selenocysteine lyase |  |
| Bbr_0911 | 1937 | 1938 | IscU protein |  |
| Bbr_0912 | 1317 | 1317 | Conserved hypothetical protein with DUF59 domain |  |
| Bbr_0913 | 431 | 469 | Glucose-1-phosphate adenylyltransferase |  |
| Bbr_0916 | 654 | 656 | HIT family protein |  |
| Bbr_0917 | 444 | 445 | PhoH protein |  |
| Bbr_0918 | 283 | 286 | Conserved hypothetical protein in uncharacterized protein family UPF0054 |  |
| Bbr_0919 | 244 | 247 | Conserved hypothetical membrane spanning protein with CBS and transporter associated domains |  |
| Bbr_0920 | 266 | 302 | GTP-binding protein era |  |
| Bbr_0921 | 2150 | 2151 | Long-chain-fatty-acid--CoA ligase |  |
| Bbr_0922 | 254 | 257 | NAD(P) transhydrogenase subunit alpha part 1 |  |
| Bbr_0923 | 382 | 395 | NAD(P) transhydrogenase alpha subunit |  |
| Bbr_0924 | 208 | 230 | NAD(P) transhydrogenase subunit beta |  |
| Bbr_0925 | 1045 | 1055 | Permease MFS superfamily |  |
| Bbr_0926 | 3713 | 3715 | LSU ribosomal protein L25P |  |
| Bbr_0927 | 319 | nd | Branched-chain amino acid aminotransferase |  |
| Bbr_0930 | 2788 | 2790 | SSU ribosomal protein S20P |  |
| Bbr_0931 | 292 | 294 | GTP-binding protein lepA |  |
| Bbr_0932 | 204 | 213 | Coproporphyrinogen oxidase (NAD) |  |
| Bbr_0936 | 283 | 288 | Glutamate synthase [NADPH] large chain |  |
| Bbr_0937 | 201 | 202 | Glutamate synthase [NADPH] small chain |  |
| Bbr_0939 | 730 | 731 | Solute binding protein of ABC transporter system for peptides |  |
| Bbr_0940 | 179 | 199 | Conserved hypothetical protein |  |
| Bbr_0941 | 445 | nd | Conserved hypothetical membrane spanning protein |  |
| Bbr_0942 | 246 | 250 | Glycosyltransferase |  |
| Bbr_0947 | 158 | 166 | Thioredoxin peroxidase |  |
| Bbr_0953 | 218 | 219 | Conserved hypothetical protein |  |
| Bbr_0954 | 289 | 292 | Conserved hypothetical protein with YfbU domain |  |
| Bbr_0955 | 299 | 305 | Conserved hypothetical protein |  |
| Bbr_0967 | 151 | 155 | tRNA (adenine-N(1)-)-methyltransferase |  |
| Bbr_0968 | 319 | 322 | Phosphohistidine phosphatase sixA |  |
| Bbr_0969 | 339 | 342 | 5-methyltetrahydropteroyltriglutamate-- homocysteine methyltransferase |  |
| Bbr_0971 | 619 | 619 | Choloylglycine hydrolase |  |
| Bbr_0972 | 202 | 202 | Glutamate-ammonia-ligase adenylyltransferase |  |
| Bbr_0973 | 874 | 879 | Aspartate carbamoyltransferase |  |
| Bbr_0974 | 1197 | 1197 | Aspartate carbamoyltransferase |  |
| Bbr_0975 | 601 | 601 | Dihydroorotase |  |
| Bbr_0976 | 280 | 282 | Orotidine 5'-phosphate decarboxylase |  |
| Bbr_0977 | 343 | 344 | Dihydroorotate dehydrogenase electron transfer subunit |  |
| Bbr_0978 | 302 | 302 | Dihydroorotate dehydrogenase, catalytic subunit |  |
| Bbr_0979 | 727 | 732 | Orotate phosphoribosyltransferase |  |
| Bbr_0981 | 171 | 181 | Transcriptional regulator, LysR family |  |
| Bbr_0984 | 232 | 233 | ATP-binding protein of ABC transporter system |  |
| Bbr_0985 | 385 | 386 | Conserved hypothetical protein with short chain dehydrogenase domain |  |
| Bbr_0988 | 220 | 220 | Aminotransferase |  |
| Bbr_0991 | 267 | 276 | Shikimate 5-dehydrogenase |  |
| Bbr_0992 | 206 | 208 | ATP-binding protein (contains P-loop) |  |
| Bbr_0993 | 472 | 473 | Conserved hypothetical protein |  |
| Bbr_0994 | 661 | 663 | Phosphoglycerate kinase |  |
| Bbr_0996 | 755 | 756 | Protein translocase subunit secG |  |
| Bbr_0997 | 240 | 241 | L-lactate dehydrogenase |  |
| Bbr_0998 | 276 | 277 | Hydrolase, HAD superfamily |  |
| Bbr_0999 | 241 | 244 | Aminotransferase |  |
| Bbr_1002 | 9009 | 9013 | Transaldolase |  |
| Bbr_1003 | 5395 | 5396 | Transketolase |  |
| Bbr_1004 | 198 | 206 | Heat-inducible transcription repressor hrcA |  |
| Bbr_1005 | 267 | 271 | Chaperone protein dnaJ |  |
| Bbr_1007 | 659 | 665 | Bacitracin resistance protein (Putative undecaprenol kinase) |  |
| Bbr_1009 | 641 | 641 | Threonyl-tRNA synthetase |  |
| Bbr_1010 | 828 | 830 | HIT family hydrolase |  |
| Bbr_1011 | 1457 | 1458 | Conserved hypothetical protein with DUF28 |  |
| Bbr_1012 | 268 | 268 | Crossover junction endodeoxyribonuclease ruvC |  |
| Bbr_1013 | 298 | 298 | Holliday junction DNA helicase ruvA |  |
| Bbr_1014 | 207 | 207 | Holliday junction DNA helicase ruvB |  |
| Bbr_1015 | 1668 | 1669 | Protein translocase subunit YajC |  |
| Bbr_1016 | 1235 | 1235 | Adenine phosphoribosyltransferase |  |
| Bbr_1017 | 629 | 631 | Succinyl-CoA synthetase beta chain |  |
| Bbr_1018 | 614 | 616 | Succinyl-CoA synthetase alpha chain |  |
| Bbr_1019 | 449 | 450 | Conserved hypothetical membrane spanning protein |  |
| Bbr_1020 | 735 | 736 | Phosphoribosylaminoimidazolecarboxamide formyltransferase/IMP cyclohydrolase |  |
| Bbr_1021 | 809 | 870 | Major intrinsic protein |  |
| Bbr_1022 | 424 | 424 | Ribosomal large subunit pseudouridine synthase B |  |
| Bbr_1023 | 315 | 315 | GTP-binding protein |  |
| Bbr_1025 | 694 | 698 | UTP--glucose-1-phosphate uridylyltransferase |  |
| Bbr_1026 | 785 | 785 | Conserved hypothetical protein |  |
| Bbr_1027 | 738 | 739 | Conserved hypothetical secreted protein |  |
| Bbr_1028 | 172 | 173 | Helicase helY |  |
| Bbr_1029 | 766 | 766 | Conserved hypothetical protein |  |
| Bbr_1033 | 671 | 671 | Transcriptional regulator, MerR family |  |
| Bbr_1034 | 834 | 835 | Signal transduction protein garA |  |
| Bbr_1035 | 251 | 253 | Conserved hypothetical protein with DUF881 domain |  |
| Bbr_1036 | 269 | 283 | Small basic protein |  |
| Bbr_1037 | 270 | 272 | Conserved hypothetical protein with DUF881 domain |  |
| Bbr_1038 | 493 | 495 | CDP-diacylglycerol--glycerol-3-phosphate 3-phosphatidyltransferase |  |
| Bbr_1039 | 523 | 525 | ATP phosphoribosyltransferase |  |
| Bbr_1040 | 1099 | 1101 | Phosphoribosyl-ATP pyrophosphatase |  |
| Bbr_1041 | 633 | 633 | Ribulose-phosphate 3-epimerase |  |
| Bbr_1042 | 500 | 502 | Prolipoprotein diacylglyceryl transferase |  |
| Bbr_1051 | 250 | 256 | Abortive infection protein AbiEi |  |
| Bbr_1052 | 326 | 328 | Conserved hypothetical protein with DUF984 domain |  |
| Bbr_1055 | 816 | 820 | Acetyltransferase (GNAT) family |  |
| Bbr_1066 | 227 | 227 | Conserved hypothetical protein with RelB antitoxin domain |  |
| Bbr_1067 | 216 | 216 | DNA damage inducible protein yafQ, possible toxin protein |  |
| Bbr_1075 | 237 | 247 | Transporter, drug/metabolite exporter family |  |
| Bbr_1076 | 283 | 286 | Hypothetical membrane spanning protein |  |
| Bbr_1077 | 332 | 335 | Hypothetical membrane spanning protein |  |
| Bbr_1078 | 686 | 688 | Lantibiotic transport ATP-binding protein |  |
| Bbr_1084 | 174 | 179 | Conserved hypothetical protein with helix-turn-helix motif |  |
| Bbr_1085 | 158 | 163 | Hypothetical membrane spanning protein |  |
| Bbr_1090 | 200 | 202 | Amino acid permease |  |
| Bbr_1091 | 279 | 283 | hypothetical protein |  |
| Bbr_1094 | 209 | 214 | Conserved hypothetical membrane spanning protein |  |
| Bbr_1097 | 198 | 199 | Phosphoribosyl-AMP cyclohydrolase |  |
| Bbr_1098 | 221 | 224 | Imidazole glycerol phosphate synthase, cyclase subunit |  |
| Bbr_1100 | 292 | 300 | Radical SAM family enzyme |  |
| Bbr_1101 | 620 | 622 | Phosphatidate cytidylyltransferase |  |
| Bbr_1102 | 1034 | 1035 | Ribosome Recycling Factor (RRF) |  |
| Bbr_1103 | 899 | 900 | Uridylate kinase |  |
| Bbr_1104 | 1828 | 1828 | Protein Translation Elongation Factor Ts (EF-Ts) |  |
| Bbr_1105 | 6043 | 6044 | SSU ribosomal protein S2P |  |
| Bbr_1106 | 245 | 246 | Peptide deformylase |  |
| Bbr_1107 | 315 | 317 | Long-chain-fatty-acid--CoA ligase |  |
| Bbr_1109 | 734 | 768 | GMP reductase |  |
| Bbr_1110 | 376 | 380 | Isocitrate dehydrogenase [NADP] |  |
| Bbr_1111 | 212 | 213 | Conserved hypothetical secreted protein |  |
| Bbr_1118 | 371 | 384 | Type II restriction-modification system restriction subunit, PstI isoschizomer |  |
| Bbr_1119 | 394 | 396 | Modification methylase |  |
| Bbr_1120 | 384 | 386 | restriction enzyme |  |
| Bbr_1121 | 264 | 265 | Cytosine methyl transferase, RM methylase bbrUIIM |  |
| Bbr_1124 | 252 | 253 | Ribosomal-protein-S18-alanine acetyltransferase |  |
| Bbr_1125 | 274 | 278 | Glycoprotease protein family |  |
| Bbr_1130 | 508 | 509 | Leucyl-tRNA synthetase |  |
| Bbr_1132 | 362 | 363 | Magnesium and cobalt transport protein corA |  |
| Bbr_1135 | 1173 | 1179 | Pyridoxamine 5'-phosphate oxidase pdxH |  |
| Bbr_1152 | 165 | nd | Hypothetical protein |  |
| Bbr_1153 | 153 | nd | Fic family protein |  |
| Bbr_1156 | 1311 | 1330 | Peptidyl-prolyl cis-trans isomerase |  |
| Bbr_1157 | 223 | 240 | GTP pyrophosphokinase/Guanosine-3',5'-bis(Diphosphate) 3'-pyrophosphohydrolase |  |
| Bbr_1158 | 787 | 793 | Deoxyuridine 5'-triphosphate nucleotidohydrolase |  |
| Bbr_1159 | 826 | 852 | Conserved hypothetical protein |  |
| Bbr_1160 | 305 | 324 | Conserved hypothetical protein |  |
| Bbr_1162 | 202 | 204 | DNA gyrase subunit A |  |
| Bbr_1168 | 231 | 231 | pfkB family carbohydrate kinase |  |
| Bbr_1170 | 324 | 325 | DNA gyrase subunit B |  |
| Bbr_1171 | 856 | nd | RNA polymerase principal sigma factor hrdB |  |
| Bbr_1174 | 203 | 215 | Serine/threonine protein kinase |  |
| Bbr_1175 | 489 | 538 | 1-acyl-sn-glycerol-3-phosphate acyltransferase |  |
| Bbr_1176 | 217 | 227 | Conserved hypothetical membrane spanning protein |  |
| Bbr_1178 | 1042 | 1049 | Protein translocase subunit secA |  |
| Bbr_1179 | 4702 | 4702 | Ribosome-associated factor Y |  |
| Bbr_1181 | 303 | nd | RecA protein |  |
| Bbr_1182 | 175 | nd | Conserved hypothetical protein |  |
| Bbr_1183 | 354 | nd | Transcriptional regulator |  |
| Bbr_1184 | 152 | nd | Competence-damage protein cinA |  |
| Bbr_1185 | 599 | 601 | CDP-diacylglycerol--glycerol-3-phosphate 3-phosphatidyltransferase |  |
| Bbr_1188 | 169 | 182 | tRNA delta(2)-isopentenylpyrophosphate transferase |  |
| Bbr_1189 | 208 | 211 | tRNA 2-methylthioadenosine synthase |  |
| Bbr_1190 | 195 | 213 | GTP pyrophosphokinase |  |
| Bbr_1192 | 523 | 525 | Conserved hypothetical membrane spanning protein in uncharacterized protein family UPF0005 |  |
| Bbr_1194 | 171 | 197 | Conserved hypothetical protein in acetyl transferase family |  |
| Bbr_1195 | 352 | 355 | Aconitate hydratase |  |
| Bbr_1197 | 210 | 213 | Conserved hypothetical secreted protein |  |
| Bbr_1198 | 215 | 216 | tRNA (Uracil-5-) -methyltransferase |  |
| Bbr_1199 | 390 | 390 | Conserved hypothetical membrane spanning protein |  |
| Bbr_1200 | 1043 | 1045 | Conserved hypothetical protein |  |
| Bbr_1202 | 1265 | 1267 | Oligopeptide transport ATP-binding protein oppD |  |
| Bbr_1203 | 1248 | 1250 | Oligopeptide transport system permease protein oppC |  |
| Bbr_1204 | 1735 | 1737 | Oligopeptide transport system permease protein oppB |  |
| Bbr_1205 | 3846 | 3847 | Oligopeptide-binding protein oppA |  |
| Bbr_1209 | 472 | 537 | Conserved hypothetical protein |  |
| Bbr_1212 | 305 | 309 | Conserved hypothetical membrane spanning protein |  |
| Bbr_1213 | 1055 | 1058 | GTP-binding protein TypA/BipA |  |
| Bbr_1224 | 179 | nd | Segregation and condensation protein ScpA |  |
| Bbr_1225 | 281 | nd | Chromosome partitioning protein parA |  |
| Bbr_1228 | 2167 | 2167 | LSU ribosomal protein L20P |  |
| Bbr_1229 | 3033 | 3034 | LSU ribosomal protein L35P |  |
| Bbr_1230 | 4456 | 4459 | Bacterial Protein Translation Initiation Factor 3 (IF-3) |  |
| Bbr_1233 | 7932 | 7937 | Glyceraldehyde 3-phosphate dehydrogenase |  |
| Bbr_1234 | 495 | 548 | Conserved hypothetical protein with YbaK / prolyl-tRNA synthetases associated domain |  |
| Bbr_1236 | 367 | 368 | Aldose 1-epimerase |  |
| Bbr_1237 | 292 | 302 | Aldose 1-epimerase |  |
| Bbr_1238 | 176 | 187 | Conserved hypothetical protein |  |
| Bbr_1239 | 235 | 242 | RNA polymerase ECF-type sigma factor |  |
| Bbr_1240 | 383 | 385 | UDP-N-acetylmuramoyl-L-alanyl-D-glutamate-- lysine ligase |  |
| Bbr_1241 | 465 | 467 | Cell wall biosynthesis-associated protein |  |
| Bbr_1244 | 446 | 448 | Folylpolyglutamate synthase/Dihydrofolate synthase |  |
| Bbr_1245 | 318 | nd | Xaa-Pro aminopeptidase |  |
| Bbr_1246 | 297 | nd | Phosphohydrolase (MutT/nudix family protein) |  |
| Bbr_1248 | 463 | 464 | Glucosamine-6-phosphate isomerase |  |
| Bbr_1251 | 284 | nd | N-acetylglucosamine repressor |  |
| Bbr_1252 | 267 | nd | pfkB family carbohydrate kinase |  |
| Bbr_1254 | 223 | 224 | D-tyrosyl-tRNA(Tyr) deacylase |  |
| Bbr_1255 | 218 | 218 | Cell division protein ftsQ |  |
| Bbr_1256 | 243 | 244 | UDP-N-acetylmuramate--alanine ligase |  |
| Bbr_1257 | 222 | 223 | UDP-N-acetylglucosamine--N-acetylmuramyl- (pentapeptide) pyrophosphoryl-undecaprenol N-acetylglucosamine transferase |  |
| Bbr_1258 | 173 | 174 | Cell division protein ftsW |  |
| Bbr_1259 | 379 | 379 | UDP-N-acetylmuramoylalanine--D-glutamate ligase |  |
| Bbr_1260 | 351 | 351 | Phospho-N-acetylmuramoyl-pentapeptide- transferase |  |
| Bbr_1261 | 336 | 337 | UDP-N-acetylmuramoyl-tripeptide--D-alanyl-D- alanine ligase |  |
| Bbr_1262 | 316 | 320 | Conserved hypothetical protein, probably involved in cell wall biosynthesis |  |
| Bbr_1263 | 289 | 289 | Peptidoglycan synthetase FtsI, penicillin-binding protein |  |
| Bbr_1264 | 447 | 448 | Conserved hypothetical protein |  |
| Bbr_1265 | 400 | 401 | S-adenosyl-methyltransferase mraW |  |
| Bbr_1266 | 343 | 343 | Cell division protein mraZ |  |
| Bbr_1267 | 234 | 236 | ATP-dependent DNA helicase |  |
| Bbr_1268 | 264 | 308 | D-3-phosphoglycerate dehydrogenase |  |
| Bbr_1269 | 393 | 397 | Conserved hypothetical protein with ATP cone domain |  |
| Bbr_1270 | 249 | 252 | LysM domain protein |  |
| Bbr_1271 | 192 | 193 | LexA repressor |  |
| Bbr_1273 | 3361 | 3362 | L-lactate dehydrogenase |  |
| Bbr_1277 | 152 | nd | Conserved hypothetical membrane spanning protein |  |
| Bbr_1278 | 586 | 589 | Glutamine synthetase |  |
| Bbr_1280 | 171 | 185 | 1-(5-phosphoribosyl)-5-[(5- phosphoribosylamino)methylideneamino] imidazole-4-carboxamide isomerase |  |
| Bbr_1281 | 262 | 264 | Imidazole glycerol phosphate synthase subunit hisH |  |
| Bbr_1282 | 367 | 368 | Conserved hypothetical protein |  |
| Bbr_1283 | 390 | 390 | Imidazoleglycerol-phosphate dehydratase |  |
| Bbr_1284 | 437 | 438 | Histidinol-phosphate aminotransferase |  |
| Bbr_1285 | 281 | 283 | Histidinol dehydrogenase |  |
| Bbr_1287 | 259 | 285 | Aldo/keto reductase family |  |
| Bbr_1293 | 569 | 571 | Myosin-crossreactive antigen |  |
| Bbr_1295 | 231 | 233 | Conserved hypothetical protein |  |
| Bbr_1296 | 244 | 246 | DNA polymerase III alpha subunit |  |
| Bbr_1298 | 346 | 355 | Ribosomal large subunit pseudouridine synthase D |  |
| Bbr_1299 | 369 | 370 | Lipoprotein signal peptidase |  |
| Bbr_1300 | 1918 | 1919 | Conserved hypothetical protein containing a repeated sequence found in lipoprotein LPP |  |
| Bbr_1301 | 664 | 664 | Conserved hypothetical membrane spanning protein with YGGT family |  |
| Bbr_1302 | 1203 | 1206 | Conserved hypothetical protein with DUF552 domain |  |
| Bbr_1303 | 828 | 828 | Cell division protein ftsZ |  |
| Bbr_1304 | 186 | 187 | tRNA-dihydrouridine synthase |  |
| Bbr_1305 | 818 | 820 | Glycyl-tRNA synthetase |  |
| Bbr_1308 | 205 | 212 | Hypothetical membrane spanning protein |  |
| Bbr_1309 | 261 | 262 | Transcriptional regulator |  |
| Bbr_1310 | 168 | 168 | Hypothetical membrane spanning protein |  |
| Bbr_1314 | 298 | 301 | Phosphomethylpyrimidine kinase/Hydroxymethylpyrimidine kinase |  |
| Bbr_1315 | 252 | 252 | Conserved hypothetical protein with DUF77 domain |  |
| Bbr_1321 | 193 | 194 | Transcriptional regulator, LacI family |  |
| Bbr_1325 | 630 | 630 | ROK family |  |
| Bbr_1327 | 385 | 392 | dTDP-rhamnosyl transferase rfbF |  |
| Bbr_1328 | 618 | 619 | Conserved hypothetical membrane spanning protein |  |
| Bbr_1329 | 163 | 167 | Fused ATP binding protein and permease of ABC transporter |  |
| Bbr_1330 | 249 | 261 | Hypothetical RNA binding protein |  |
| Bbr_1337 | 245 | nd | AAA family ATPase |  |
| Bbr_1344 | 178 | nd | Glutamate transport system permease protein gluC |  |
| Bbr_1345 | 272 | nd | Glutamate-binding protein gluB |  |
| Bbr_1346 | 229 | nd | Glutamate transport ATP-binding protein gluA |  |
| Bbr_1348 | 457 | 458 | Aspartyl-tRNA synthetase |  |
| Bbr_1349 | 361 | 362 | Histidyl-tRNA synthetase |  |
| Bbr_1350 | 725 | 726 | Conserved hypothetical protein with DUF349 domain |  |
| Bbr_1356 | 569 | 570 | Negative regulator of genetic competence clpC/mecB |  |
| Bbr_1357 | 227 | 335 | Universal stress protein family |  |
| Bbr_1358 | 553 | 561 | Conserved hypothetical protein |  |
| Bbr_1359 | 452 | 454 | Cold shock protein |  |
| Bbr_1360 | 235 | nd | Two component system histidine kinase |  |
| Bbr_1361 | 809 | nd | Two-component response regulator |  |
| Bbr_1362 | 363 | nd | Conserved hypothetical membrane spanning protein |  |
| Bbr_1364 | 6121 | 6124 | 60 kDa chaperonin GroEL |  |
| Bbr_1365 | 1237 | 1238 | Cold shock protein |  |
| Bbr_1366 | 560 | 564 | Conserved hypothetical protein |  |
| Bbr_1367 | 283 | nd | Uracil-DNA glycosylase |  |
| Bbr_1368 | 318 | nd | MoxR protein |  |
| Bbr_1369 | 201 | nd | Conserved hypothetical protein |  |
| Bbr_1370 | 404 | nd | Conserved hypothetical protein |  |
| Bbr_1371 | 366 | nd | Conserved hypothetical membrane spanning protein |  |
| Bbr_1372 | 267 | nd | Conserved hypothetical membrane spanning protein with a von Willebrand factor type A domain |  |
| Bbr_1373 | 310 | nd | Conserved hypothetical secreted protein |  |
| Bbr_1376 | 548 | 550 | Permease protein of ABC transporter system |  |
| Bbr_1377 | 582 | nd | Adenylosuccinate lyase |  |
| Bbr_1378 | 215 | nd | Conserved hypothetical membrane spanning protein in uncharacterized protein UPF0104 family |  |
| Bbr_1379 | 8773 | 8773 | DNA-binding protein HU |  |
| Bbr_1380 | 205 | nd | Conserved hypothetical protein with DUF275 domain |  |
| Bbr_1381 | 499 | nd | Conserved hypothetical protein with DUF797 domain |  |
| Bbr_1382 | 368 | nd | Inositol monophosphatase family protein |  |
| Bbr_1395 | 283 | nd | Haloacid dehalogenase-like hydrolase (HAD superfamily) |  |
| Bbr_1396 | 162 | nd | Methionyl-tRNA formyltransferase |  |
| Bbr_1397 | 466 | 470 | Dihydroxy-acid dehydratase |  |
| Bbr_1398 | 2198 | 2202 | DNA-directed RNA polymerase omega chain |  |
| Bbr_1399 | 650 | 653 | S-adenosylmethionine synthetase |  |
| Bbr_1407 | 279 | nd | CRISPR-associated protein Cas4 |  |
| Bbr_1408 | 664 | nd | CRISPR-associated protein |  |
| Bbr_1409 | 316 | nd | CRISPR-associated protein |  |
| Bbr_1410 | 394 | nd | CRISPR-associated protein |  |
| Bbr_1411 | 176 | nd | CRISPR-associated helicase Cas3 |  |
| Bbr_1414 | 416 | 418 | Isoleucyl-tRNA synthetase |  |
| Bbr_1433 | 215 | 215 | ATP-binding protein of ABC transporter system |  |
| Bbr_1434 | 247 | 248 | ATP-binding protein of ABC transporter system |  |
| Bbr_1435 | 244 | 245 | Transcriptional regulator, MarR family |  |
| Bbr_1444 | 309 | 311 | Glutamate--cysteine ligase |  |
| Bbr_1445 | 448 | 450 | Activator of (R)-2-hydroxyglutaryl-CoA dehydratase |  |
| Bbr_1446 | 595 | 597 | Anaerobic ribonucleoside-triphosphate reductase activating protein |  |
| Bbr_1447 | 1346 | 1349 | Anaerobic ribonucleoside-triphosphate reductase |  |
| Bbr_1448 | 210 | 216 | Exodeoxyribonuclease VII large subunit |  |
| Bbr_1449 | 366 | 384 | Exodeoxyribonuclease VII small subunit |  |
| Bbr_1450 | 277 | 346 | NAD(P)H oxidoreductase |  |
| Bbr_1451 | 361 | 380 | long chain fatty acid CoA ligase |  |
| Bbr_1456 | 179 | 180 | Aminotransferase |  |
| Bbr_1461 | 251 | 259 | 2,3,4,5-tetrahydropyridine-2-carboxylate N-succinyltransferase |  |
| Bbr_1463 | 1089 | 1089 | Methionine aminopeptidase |  |
| Bbr_1464 | 2302 | 2307 | Conserved hypothetical membrane spanning protein |  |
| Bbr_1465 | 675 | 676 | Zinc metalloprotease |  |
| Bbr_1467 | 403 | 404 | Prolyl-tRNA synthetase |  |
| Bbr_1470 | 172 | nd | Helicase |  |
| Bbr_1471 | 605 | 608 | Oligoribonuclease |  |
| Bbr_1472 | 1013 | 1014 | Inosine-5'-monophosphate dehydrogenase |  |
| Bbr_1473 | 407 | 408 | Undecaprenyl-phosphate alpha-N-acetylglucosaminephosphotransferase |  |
| Bbr_1474 | 574 | 574 | Sua5/YciO/YrdC/YwlC family protein |  |
| Bbr_1475 | 202 | nd | Branched-chain amino acid transport ATP-binding protein livF |  |
| Bbr_1476 | 424 | nd | Branched-chain amino acid transport ATP-binding protein livG |  |
| Bbr_1477 | 251 | nd | Branched-chain amino acid transport system permease protein livM |  |
| Bbr_1478 | 226 | nd | Branched-chain amino acid transport system permease protein livH |  |
| Bbr_1479 | 433 | nd | Leucine-, isoleucine-, valine-, threonine-, and alanine-binding protein |  |
| Bbr_1480 | 171 | nd | Peptide release factor-glutamine N5-methyltransferase |  |
| Bbr_1481 | 247 | nd | Bacterial Peptide Chain Release Factor 1 (RF-1) |  |
| Bbr_1482 | 6673 | 6676 | LSU ribosomal protein L31P |  |
| Bbr_1488 | 407 | 407 | Glucokinase/Xylose repressor |  |
| Bbr_1489 | 214 | nd | Conserved hypothetical membrane spanning protein, MFS member |  |
| Bbr_1490 | 678 | nd | ATP-binding protein of ABC transporter system |  |
| Bbr_1492 | 194 | nd | Conserved hypothetical membrane spanning protein |  |
| Bbr_1493 | 208 | nd | Dihydroneopterin aldolase/2-amino-4-hydroxy-6-hydroxymethyldihydropteridine pyrophosphokinase |  |
| Bbr_1494 | 270 | nd | Dihydropteroate synthase |  |
| Bbr_1495 | 357 | nd | GTP cyclohydrolase I |  |
| Bbr_1496 | 1395 | nd | Cell division protein ftsH |  |
| Bbr_1497 | 2377 | nd | Hypoxanthine-guanine phosphoribosyltransferase |  |
| Bbr_1499 | 211 | nd | Conserved hypothetical secreted protein with D-Ala-D-Ala carboxypeptidase 3 (S13) domain |  |
| Bbr_1502 | 383 | nd | N-acetylglucosaminyltransferase |  |
| Bbr_1503 | 350 | nd | Conserved hypothetical membrane spanning protein with DUF20 domain |  |
| Bbr_1504 | 220 | 244 | Glycosyltransferase |  |
| Bbr_1505 | 1144 | 1156 | Lactaldehyde reductase |  |
| Bbr_1506 | 246 | 275 | Cyclopropane-fatty-acyl-phospholipid synthase |  |
| Bbr_1507 | 569 | nd | tRNA (m(7)G46) methyltransferase |  |
| Bbr_1517 | 343 | nd | Phosphoserine phosphatase |  |
| Bbr_1523 | 220 | nd | MerR family regulatory protein |  |
| Bbr_1524 | 379 | nd | Conserved hypothetical protein |  |
| Bbr_1532 | 273 | nd | DNA processing chain A |  |
| Bbr_1533 | 160 | nd | Hypothetical protein |  |
| Bbr_1534 | 393 | nd | ATP-dependent DNA helicase rep |  |
| Bbr_1535 | 194 | nd | Conserved hypothetical protein with ABC transporter domain |  |
| Bbr_1551 | 986 | nd | Galactoside symporter |  |
| Bbr_1552 | 427 | nd | Beta-galactosidase |  |
| Bbr_1554 | 815 | 819 | Solute-binding protein of ABC transporter system (lactose) |  |
| Bbr_1562 | 808 | 853 | Protein tyrosine phosphatase |  |
| Bbr_1563 | 1242 | 1245 | Dihydrofolate reductase |  |
| Bbr_1564 | 479 | 483 | Thymidylate synthase |  |
| Bbr_1565 | 1025 | 1073 | Conserved hypothetical protein, OsmC-like protein |  |
| Bbr_1566 | 1343 | 1350 | Universal stress protein family |  |
| Bbr_1567 | 945 | 946 | Conserved hypothetical secreted protein with NlpC/P60 family domain |  |
| Bbr_1568 | 499 | 500 | Conserved hypothetical secreted protein with NlpC/P60 family domain |  |
| Bbr_1569 | 1043 | 1043 | Conserved hypothetical protein with CHAP domain |  |
| Bbr_1571 | 289 | nd | Conserved hypothetical membrane spanning protein |  |
| Bbr_1573 | 345 | 364 | Phosphate transport system protein phoU-like protein |  |
| Bbr_1574 | 4431 | 4433 | Phosphoglycerate mutase |  |
| Bbr_1576 | 414 | 415 | Lysyl-tRNA synthetase |  |
| Bbr_1580 | 308 | 318 | Conserved hypothetical membrane spanning protein |  |
| Bbr_1581 | 2358 | 2361 | Conserved hypothetical membrane spanning protein |  |
| Bbr_1582 | 1907 | 1909 | Conserved hypothetical membrane spanning protein with PspC domain |  |
| Bbr_1583 | 278 | 284 | Histidine kinase sensor of two component system |  |
| Bbr_1584 | 264 | 300 | Response regulator of two-component system |  |
| Bbr_1585 | 1626 | 1660 | UDP-glucose 4-epimerase |  |
| Bbr_1588 | 256 | 258 | Permease protein of ABC transporter system for sugars |  |
| Bbr_1589 | 333 | 335 | Permease protein of ABC transporter system for sugars |  |
| Bbr_1590 | 1700 | 1701 | Solute-binding protein of ABC transporter system for sugars |  |
| Bbr_1591 | 809 | 809 | Seryl-tRNA synthetase |  |
| Bbr_1592 | 395 | 474 | Conserved hypothetical secreted protein with presumed diacylglycerol kinase catalytic domain |  |
| Bbr_1593 | 590 | 648 | Transcription antiterminator, BglG family |  |
| Bbr_1594 | 1090 | 1093 | PTS system, fructose/glucose-specific IIABC component |  |
| Bbr_1595 | 894 | 897 | Phosphoglucomutase |  |
| Bbr_1597 | 761 | 762 | Rrf2 family protein |  |
| Bbr_1598 | 245 | nd | Pyridine nucleotide-disulfide oxidoreductase family protein |  |
| Bbr_1599 | 220 | nd | Ribonuclease HI |  |
| Bbr_1600 | 715 | 736 | Ribose 5-phosphate isomerase |  |
| Bbr_1602 | 710 | 712 | Conserved hypothetical secreted protein |  |
| Bbr_1604 | 314 | nd | Riboflavin kinase/FMN adenylyltransferase |  |
| Bbr_1605 | 209 | nd | tRNA pseudouridine synthase B |  |
| Bbr_1606 | 356 | nd | Ribosome-binding factor A |  |
| Bbr_1607 | 643 | 645 | Bacterial Protein Translation Initiation Factor 2 (IF-2) |  |
| Bbr_1608 | 269 | 271 | N utilization substance protein A |  |
| Bbr_1611 | 286 | nd | Conserved hypothetical protein in transglutaminase family |  |
| Bbr_1614 | 4183 | 4184 | 50S ribosomal protein L17 |  |
| Bbr_1615 | 5877 | 5878 | DNA-directed RNA polymerase alpha chain |  |
| Bbr_1616 | 7429 | 7429 | SSU ribosomal protein S11P |  |
| Bbr_1617 | 7871 | 7882 | SSU ribosomal protein S13P |  |
| Bbr_1618 | 21091 | 21097 | LSU ribosomal protein L36P |  |
| Bbr_1619 | 16717 | 16721 | Bacterial Protein Translation Initiation Factor 1 (IF-1) |  |
| Bbr_1620 | 1326 | 1326 | Adenylate kinase |  |
| Bbr_1621 | 1801 | 1802 | Protein translocase subunit secY |  |
| Bbr_1622 | 8533 | 8537 | 50S ribosomal protein L15 |  |
| Bbr_1623 | 16550 | 16551 | 50S ribosomal protein L30 |  |
| Bbr_1624 | 7487 | 7490 | 30S ribosomal protein S5 |  |
| Bbr_1625 | 8363 | 8368 | 50S ribosomal protein L18 |  |
| Bbr_1626 | 4661 | 4661 | 50S ribosomal protein L6 |  |
| Bbr_1627 | 9150 | 9156 | 30S ribosomal protein S8 |  |
| Bbr_1628 | 15628 | 15631 | 30S ribosomal protein S14-1 |  |
| Bbr_1629 | 11034 | 11038 | 50S ribosomal protein L5 |  |
| Bbr_1630 | 9299 | 9308 | 50S ribosomal protein L24 |  |
| Bbr_1631 | 9153 | 9156 | 50S ribosomal protein L14 |  |
| Bbr_1632 | 9358 | 9360 | 30S ribosomal protein S17 |  |
| Bbr_1633 | 2697 | 2698 | 50S ribosomal protein L29 |  |
| Bbr_1634 | 3282 | 3282 | 50S ribosomal protein L16 |  |
| Bbr_1635 | 3518 | 3518 | 30S ribosomal protein S3 |  |
| Bbr_1636 | 7160 | 7161 | 50S ribosomal protein L22 |  |
| Bbr_1637 | 10131 | 10133 | 30S ribosomal protein S19 |  |
| Bbr_1638 | 4821 | 4822 | 50S ribosomal protein L2 |  |
| Bbr_1639 | 9368 | 9371 | 50S ribosomal protein L23 |  |
| Bbr_1640 | 9455 | 9458 | 50S ribosomal protein L4 |  |
| Bbr_1641 | 10211 | 10214 | 50S ribosomal protein L3 |  |
| Bbr_1642 | 13021 | 13024 | 30S ribosomal protein S10 |  |
| Bbr_1645 | 344 | 347 | Aldehyde-alcohol dehydrogenase 2 |  |
| Bbr_1648 | 11096 | 11102 | 30S ribosomal protein S9 |  |
| Bbr_1649 | 20520 | 20525 | 50S ribosomal protein L13 |  |
| Bbr_1650 | 545 | 547 | 4-alpha-glucanotransferase |  |
| Bbr_1653 | 178 | nd | D-3-phosphoglycerate dehydrogenase |  |
| Bbr_1664 | 407 | nd | Ferredoxin |  |
| Bbr_1665 | 168 | nd | Amino acid permease |  |
| Bbr_1666 | 243 | nd | UDP-N-acetylenolpyruvoylglucosamine reductase |  |
| Bbr_1667 | 6796 | 6805 | LSU ribosomal protein L33P |  |
| Bbr_1668 | 4901 | 4910 | 10 kDa chaperonin GroES |  |
| Bbr_1669 | 576 | nd | Conserved hypothetical membrane spanning protein |  |
| Bbr_1670a | 1640 | nd |  |  |
| Bbr_1673 | 292 | 294 | DNA and RNA helicase related protein |  |
| Bbr_1674 | 294 | 295 | Conserved hypothetical protein with FHA domain |  |
| Bbr_1675 | 19861 | 19864 | LSU ribosomal protein L12P (L7/L12) |  |
| Bbr_1676 | 16072 | 16073 | LSU ribosomal protein L10P |  |
| Bbr_1677 | 154 | nd | Conserved hypothetical protein |  |
| Bbr_1678 | 1058 | 1063 | Guanine-hypoxanthine permease |  |
| Bbr_1682 | 307 | nd | Phosphate-binding protein |  |
| Bbr_1686 | 337 | nd | 5'-methylthioadenosine nucleosidase/S-adenosylhomocysteine nucleosidase |  |
| Bbr_1687 | 227 | nd | 3-deoxy-7-phosphoheptulonate synthase |  |
| Bbr_1700 | 633 | 633 | Aminopeptidase C |  |
| Bbr_1701 | 364 | 366 | Conserved hypothetical protein |  |
| Bbr_1702 | 225 | nd | Pyrrolidone-carboxylate peptidase |  |
| Bbr_1703 | 163 | nd | 2-C-methyl-D-erythritol 4-phosphate cytidylyltransferase |  |
| Bbr_1709 | 2481 | 2484 | LSU ribosomal protein L28P |  |
| Bbr_1710 | 2146 | 2150 | Ribokinase |  |
| Bbr_1713 | 257 | nd | Hypothetical protein |  |
| Bbr_1714 | 310 | nd | Conserved hypothetical protein with RelB antitoxin domain |  |
| Bbr_1722 | 509 | 659 | BioY protein |  |
| Bbr_1723 | 485 | 533 | Conserved hypothetical protein with possible biotin-(acetyl-CoA carboxylase) ligase domain |  |
| Bbr_1726 | 10404 | 10407 | LSU ribosomal protein L1P |  |
| Bbr_1727 | 19817 | 19820 | LSU ribosomal protein L11P |  |
| Bbr_1729 | 1147 | 1148 | Transcription antitermination protein nusG |  |
| Bbr_1730 | 912 | 912 | Protein translocase subunit secE |  |
| Bbr_1731 | 613 | 615 | Aspartate aminotransferase |  |
| Bbr_1733 | 234 | nd | Glutamate 5-kinase |  |
| Bbr_1734 | 391 | nd | GTP-binding protein, GTP1/OBG family |  |
| Bbr_1735 | 6200 | 6201 | LSU ribosomal protein L27P |  |
| Bbr_1736 | 9197 | 9199 | LSU ribosomal protein L21P |  |
| Bbr_1737 | 270 | nd | Ribonuclease G |  |
| Bbr_1738 | 205 | nd | Succinyl-diaminopimelate desuccinylase |  |
| Bbr_1746 | 199 | nd | Transporter |  |
| Bbr_1747 | 209 | nd | ATP-binding protein of ABC transporter system |  |
| Bbr_1751 | 700 | 702 | Homoserine dehydrogenase |  |
| Bbr_1753 | 484 | 485 | Diaminopimelate decarboxylase |  |
| Bbr_1754 | 474 | 475 | Arginyl-tRNA synthetase |  |
| Bbr_1755 | 385 | nd | Transcriptional regulator, TetR family |  |
| Bbr_1757 | 343 | 362 | UDP-N-acetylglucosamine 1-carboxyvinyltransferase |  |
| Bbr_1758 | 259 | nd | NADH oxidase H2O-forming |  |
| Bbr_1759 | 264 | nd | Solute binding protein of ABC transporter system for amino acids |  |
| Bbr_1764 | 180 | nd | Dihydroorotate dehydrogenase |  |
| Bbr_1769 | 211 | 214 | Phosphoesterase |  |
| Bbr_1770 | 284 | 286 | Polyphosphate kinase |  |
| Bbr_1771 | 257 | nd | Phosphohydrolase (MutT/nudix family protein) |  |
| Bbr_1772 | 220 | nd | Conserved hypothetical protein |  |
| Bbr_1777 | 936 | 946 | Uracil phosphoribosyltransferase |  |
| Bbr_1778 | 172 | nd | Conserved hypothetical protein |  |
| Bbr_1779 | 338 | 380 | Glycerophosphoryl diester phosphodiesterase |  |
| Bbr_1783 | 579 | 584 | Fumarylacetoacetate hydrolase family protein |  |
| Bbr_1784 | 421 | 423 | Conserved hypothetical membrane spanning protein |  |
| Bbr_1786 | 268 | nd | UDP-galactopyranose mutase |  |
| Bbr_1788 | 234 | nd | Glycosyltransferase involved in cell wall biogenesis |  |
| Bbr_1791 | 160 | nd | Phosphoglycerol transferase |  |
| Bbr_1792 | 292 | 302 | Glycosyltransferase |  |
| Bbr_1793 | 666 | 667 | ATP-binding protein ABC transporter system for polysaccharides |  |
| Bbr_1794 | 597 | 598 | Permease protein of ABC transporter systemfor polysaccharides |  |
| Bbr_1795 | 497 | 499 | Alpha-L-Rha alpha-1,2-L-rhamnosyltransferase/alpha-L-Rha alpha-1,3-L-rhamnosyltransferase |  |
| Bbr_1796 | 406 | 414 | Glycosyltransferase |  |
| Bbr_1797 | 521 | 525 | Glucose-1-phosphate thymidylyltransferase |  |
| Bbr_1798 | 1134 | 1135 | dTDP-4-dehydrorhamnose 3,5-epimerase/dTDP-4-dehydrorhamnose reductase |  |
| Bbr_1799 | 1519 | 1522 | dTDP-glucose 4,6-dehydratase |  |
| Bbr_1800 | 170 | 172 | Conserved hypothetical membrane spanning protein |  |
| Bbr_1801 | 325 | 385 | Glycosyltransferase involved in cell wall biogenesis |  |
| Bbr_1802 | 369 | 396 | Glycosyl hydrolases family 25,lysozyme |  |
| Bbr_1803 | 556 | 558 | Transcriptional regulator, LytR family |  |
| Bbr_1804 | 412 | 415 | Permease protein of ABC transporter system |  |
| Bbr_1805 | 251 | 253 | ATP-binding protein of ABC transporter system |  |
| Bbr_1807 | 169 | nd | Conserved hypothetical protein |  |
| Bbr_1808 | 869 | 870 | Kup system potassium uptake protein |  |
| Bbr_1809 | 249 | nd | DNase, TatD family |  |
| Bbr_1818 | 206 | 222 | Aminopeptidase C |  |
| Bbr_1820 | 598 | 599 | Methionyl-tRNA synthetase |  |
| Bbr_1823 | 260 | nd | Tetrapyrrole (Corrin/Porphyrin) methylase family protein |  |
| Bbr_1830 | 212 | nd | Possible sugar O-acetyltransferase |  |
| Bbr_1831 | 190 | nd | Transcriptional regulator, LacI family |  |
| Bbr_1839 | 174 | nd | Amino acid permease |  |
| Bbr_1842 | 242 | 265 | Amino acid permease |  |
| Bbr_1843 | 240 | 448 | Conserved hypothetical membrane spanning protein |  |
| Bbr_1844 | 830 | 849 | Permease protein of ABC transporter system for sugars |  |
| Bbr_1845 | 1705 | 1706 | Permease protein of ABC transporter system for sugars |  |
| Bbr_1846 | 301 | nd | Transcriptional regulator, LacI family |  |
| Bbr_1847 | 2907 | 2909 | Solute binding protein of ABC transporter system for sugars |  |
| Bbr_1848 | 262 | nd | Phosphoglycerate mutase family protein |  |
| Bbr_1849 | 242 | nd | Conserved hypothetical protein |  |
| Bbr_1850 | 593 | nd | Chromate reductase/NADPH-dependent FMN reductase/Oxygen-insensitive NADPH nitroreductase |  |
| Bbr_1851 | 181 | nd | Phage infection protein |  |
| Bbr_1853 | 157 | 172 | SIR2 family protein |  |
| Bbr_1854 | 162 | 170 | Threonine dehydratase |  |
| Bbr_1855 | 188 | nd | alpha-glucosidase |  |
| Bbr_1860 | 165 | nd | Solute binding protein of ABC transporter system for sugars |  |
| Bbr_1867 | 810 | nd | Raffinose-binding protein |  |
| Bbr_1874 | 349 | 351 | Conserved hypothetical membrane spanning protein |  |
| Bbr_1875 | 517 | 520 | Deoxycytidine triphosphate deaminase |  |
| Bbr_1876 | 242 | 245 | Conserved hypothetical protein |  |
| Bbr_1877 | 200 | 202 | Calcium-transporting ATPase |  |
| Bbr_1879 | 249 | nd | PTS system, glucose-specific IIABC component |  |
| Bbr_1880 | 441 | nd | PTS system, N-acetylglucosamine-specific IIBC component |  |
| Bbr_1882 | 539 | 547 | SpoU rRNA methylase family protein |  |
| Bbr_1885 | 236 | nd | Conserved hypothetical protein |  |
| Bbr_1890 | 4265 | 4267 | ATP-binding protein of ABC transporter system for sugars |  |
| Bbr_1891 | 278 | nd | Transcriptional regulator, GntR family |  |
| Bbr_1892 | 890 | 891 | PTS system, IIC component |  |
| Bbr_1893 | 3131 | 3149 | PTS system, IIB component |  |
| Bbr_1894 | 5044 | 5047 | PTS system, IIA component |  |
| Bbr_1898 | 728 | nd | Ribonucleoside-diphosphate reductase beta chain |  |
| Bbr_1899 | 1171 | nd | Ribonucleoside-diphosphate reductase alpha chain |  |
| Bbr_1900 | 1306 | nd | NrdI protein |  |
| Bbr_1901 | 1068 | nd | Glutaredoxin nrdH |  |
| Bbr_1909 | 351 | nd | Conserved hypothetical protein |  |
| Bbr_1910 | 262 | nd | Conserved hypothetical secreted protein with G5 and DUF348 domains |  |
| Bbr_1911 | 161 | nd | Dimethyladenosine transferase |  |
| Bbr_1912 | 269 | nd | 4-diphosphocytidyl-2-C-methyl-D-erythritol kinase |  |
| Bbr_1913 | 602 | nd | Conserved hypothetical protein |  |
| Bbr_1914 | 220 | 236 | tRNA nucleotidyltransferase |  |
| Bbr_1915 | 410 | 412 | Phosphohydrolase (MutT/nudix family protein) |  |
| Bbr_1916 | 199 | 201 | Conserved hypothetical secreted protein |  |
| Bbr_1917 | 256 | 257 | Conserved hypothetical membrane spanning protein with virulence factor mviN domain |  |
| Bbr_1918 | 215 | 221 | Thioredoxin reductase |  |
| Bbr_1919 | 363 | 368 | Chromosome partitioning protein parB |  |
| Bbr_1920 | 386 | 393 | Chromosome partitioning protein parA |  |
| Bbr_1921 | 265 | 267 | Methyltransferase gidB (Glucose inhibited division protein B) |  |
| Bbr_1922 | 571 | 571 | Jag protein |  |
| Bbr_1923 | 1440 | 1444 | Inner membrane protein (Preprotein translocase subunit YidC) |  |
| Bbr_1925 | 2472 | 2473 | Ribonuclease P protein component |  |
| Bbr_1926 | 1449 | 1451 | LSU ribosomal protein L34P |  |
