# Supplementary figures and images for: Global transcriptional landscape and promoter mapping of the gut commensal Bifidobacterium breve UCC2003
Source: BMC Genomics. 2017 Dec 28;18:991. doi: 10.1186/s12864-017-4387-x (PMC5746004; doi:10.1186/s12864-017-4387-x)

a)

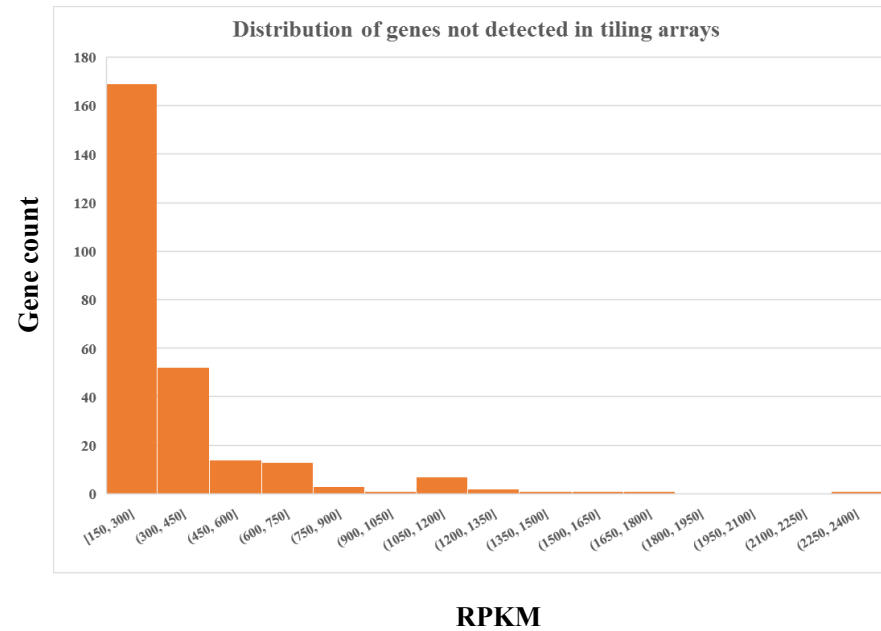

b)

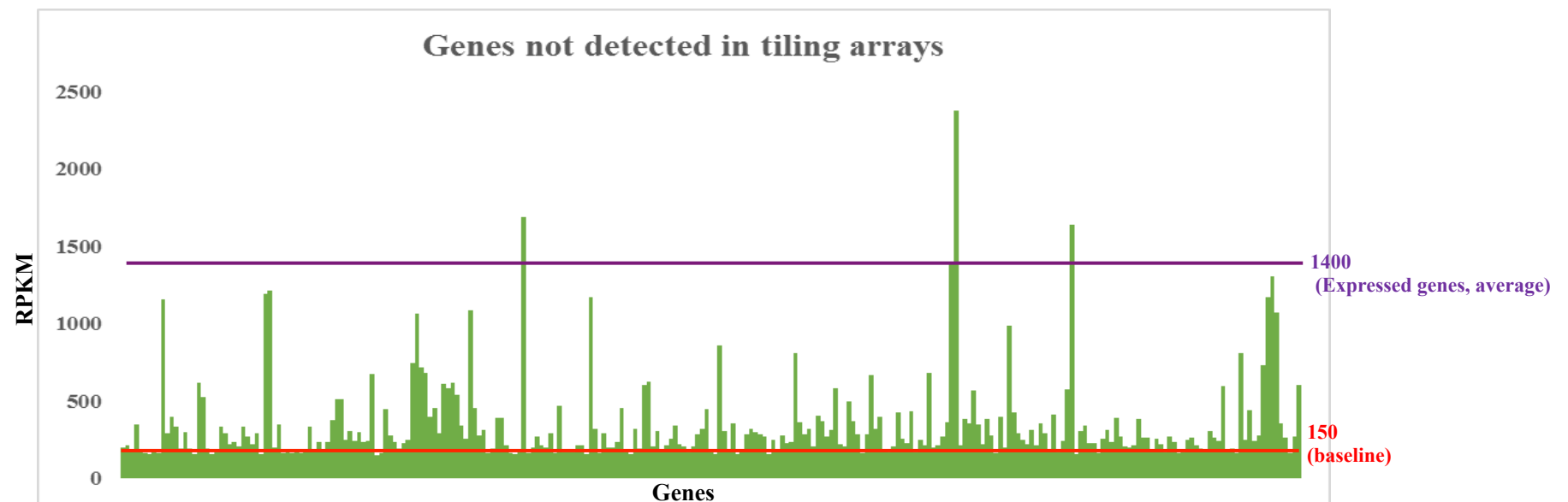

Supplement: Supplementary file 2 — RNA-Seq and tiling array comparisons. a) Bar chart showing B. breve UCC2003 genes detected as transcribed in RNA-Seq, but not in tiling arrays with associate gene count and level of expression (RPKM). b) Distribution of genes exhibiting discrepant transcription between RNA-Seq and tiling array approaches as grouped by level of transcription (RPKM). A red horizontal line indicates the baseline of transcription background, while in purple the average RPKM of transcribed genes is indicated. (PDF 94 kb) [file 12864_2017_4387_MOESM2_ESM.pdf]

a)

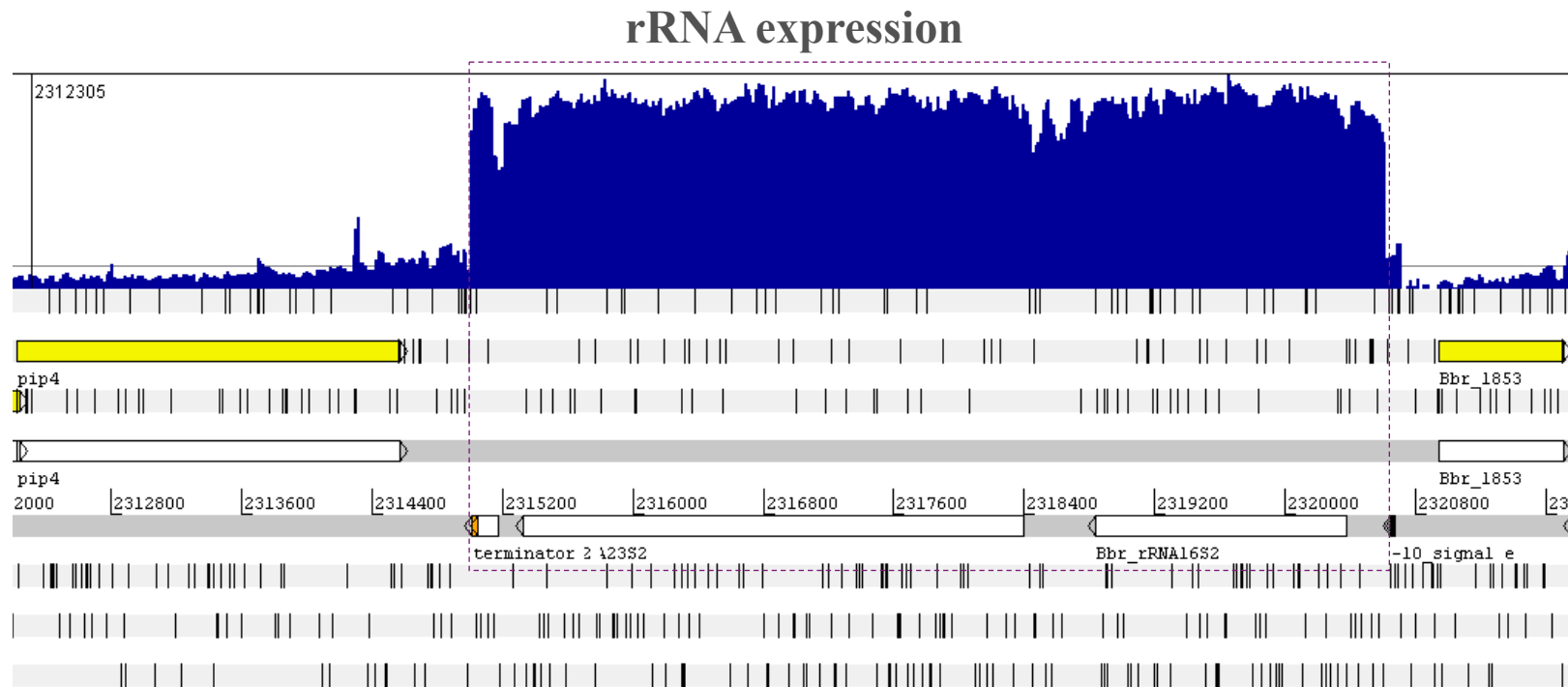

b)

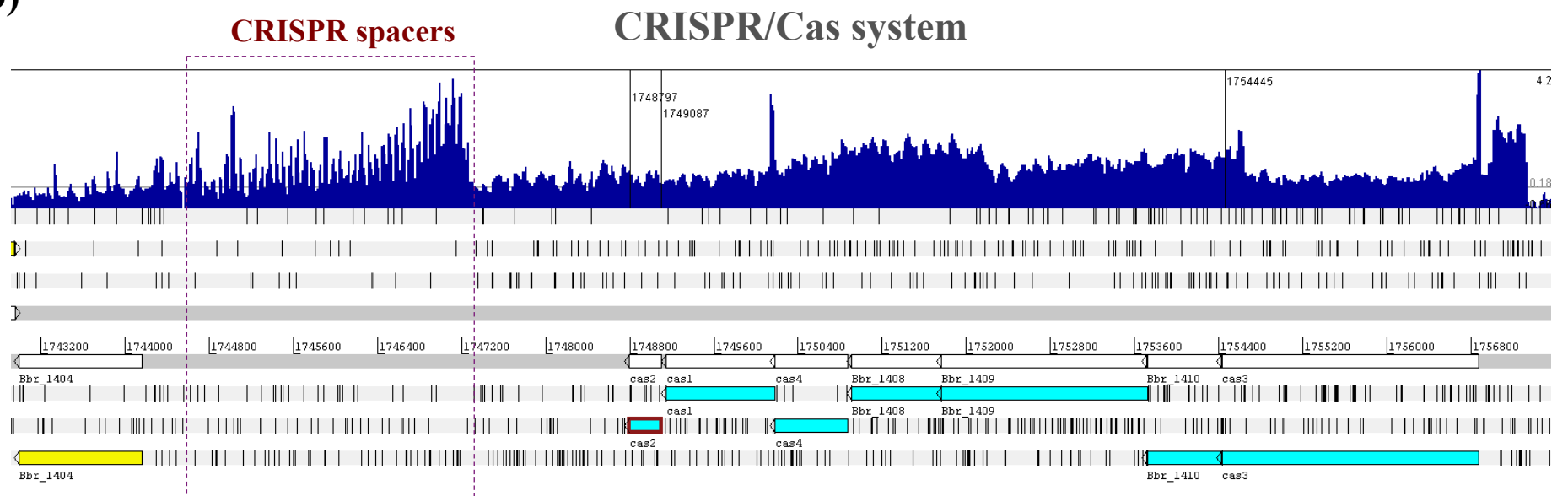

Supplement: Supplementary file 6 — B. breve ribosomal operon and CRISPR-Cas system transcription. Artemis plot showing the level of transcription of a) the rRNA operon and b) the CRISPR/Cas system in B. breve UCC2003 as detected in tiling arrays. The relative TU is indicated by a dashed purple line. (PDF 282 kb) [file 12864_2017_4387_MOESM6_ESM.pdf]

a)

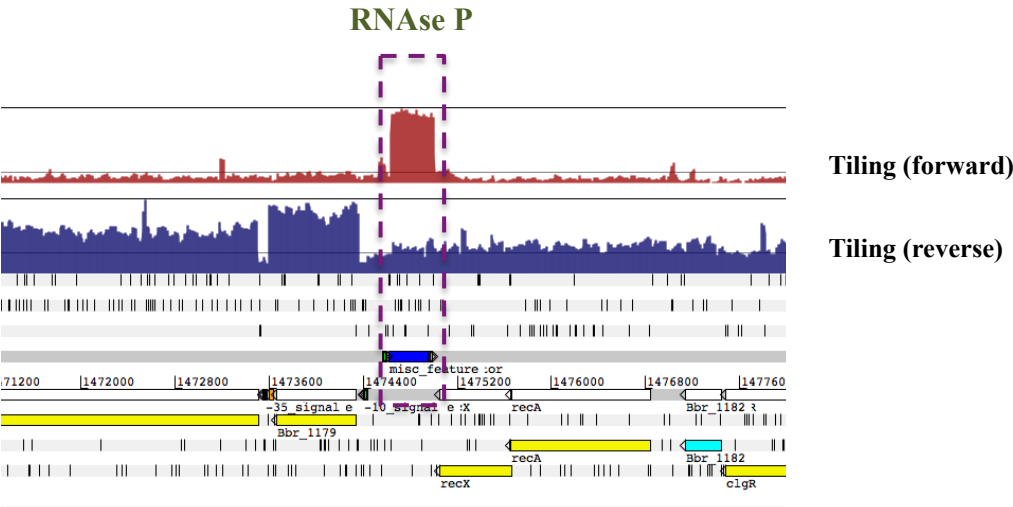

b)

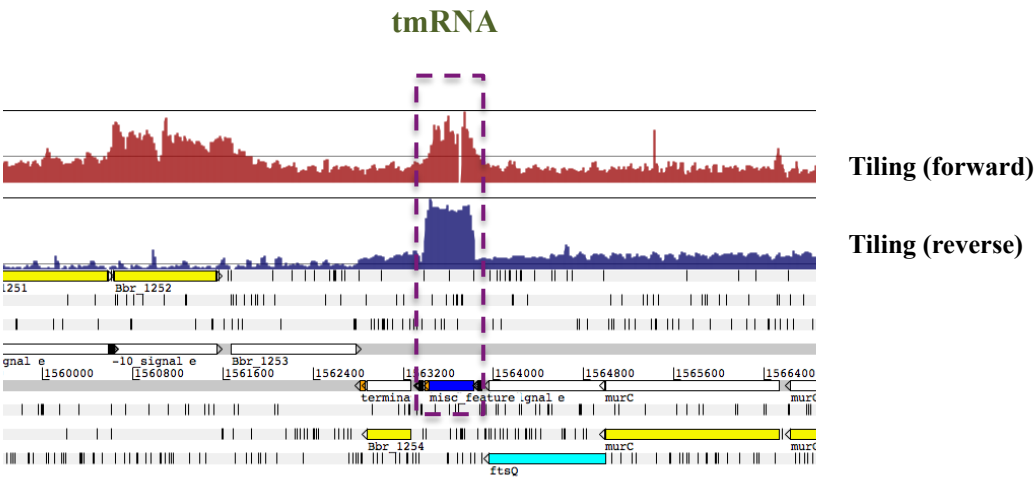

c)

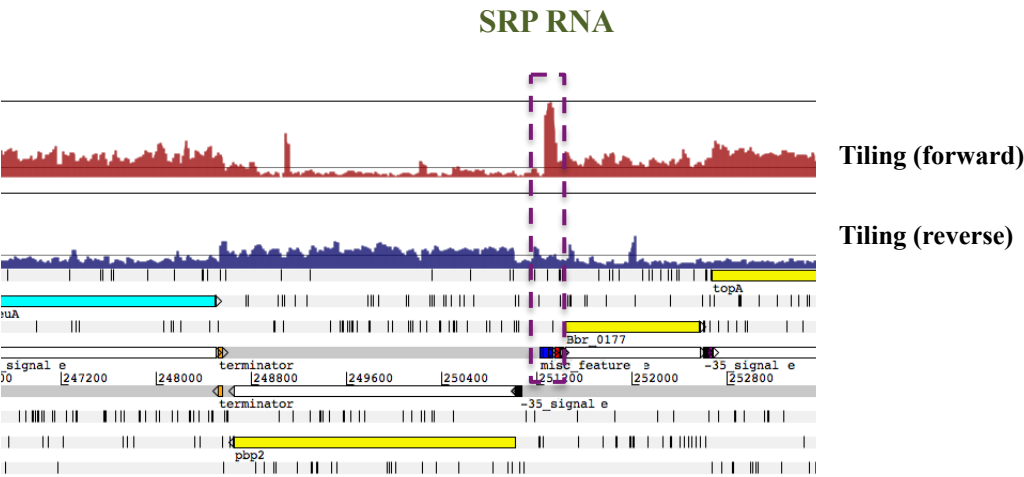

Supplement: Supplementary file 7 — B. breve sRNA expression. Artemis plot showing the sRNA transcription in B. breve of a) Ribonuclease P, b) tmRNA, and c) 4.5S SRP RNA. In all cases tiling array signals of forward (red) and reverse (blue) strand are indicated. (PDF 701 kb) [file 12864_2017_4387_MOESM7_ESM.pdf]
