# Supplementary material for: Global transcriptional landscape and promoter mapping of the gut commensal Bifidobacterium breve UCC2003
Source: BMC Genomics. 2017 Dec 28;18:991. doi: 10.1186/s12864-017-4387-x (PMC5746004; doi:10.1186/s12864-017-4387-x)
Supplement: Supplementary file 3 — Predicted transcriptional units (TUs) in B. breve and associated promoters. A .docx document containing the list of predicted transcriptional units (TUs) in B. breve and corresponding (predicted) promoters. For each TU also the transcription level and transcriptional termination is indicated. (DOCX 143 kb) [file 12864_2017_4387_MOESM3_ESM.docx]

**Table S2. Predicted transcriptional units (TUs) in *B. breve* and associated promoters.**

| **Locus** | **Strand** | **Termination** | **Level** | **5'-UTR** | **3'-UTR** | **-35** | **-10** | **Spacer** |
| --- | --- | --- | --- | --- | --- | --- | --- | --- |
| Bbr_0002 | forward | loop | low | 83 | 2 | CATACA | GATAAT | 18 |
| Bbr_0005-07 | forward | rho-indep | low | 116 | 21 | ATGGCG | TAGAAT | 18 |
| Bbr_0010 | forward | loop | low | 73 | 350 | TTGATT | TATATT | 17 |
| Bbr_0012 | reverse | loop | low | 40 | 42 | TTGTCA | TACAAT | 17 |
| Bbr_0016 | reverse | rho-indep | high | 38 | 17 | CGGCCC | TAGAAT | 19 |
| Bbr_0026-27 | reverse | loop | low | 117 | 11 | TTGACC | TATGGT | 17 |
| Bbr_0034 | reverse | loop | high | 38 | 57 | GGCCCG | TAGAAT | 18 |
| Bbr_0037 | reverse | nd | low | 34 | 50.5 | GCGAAT | TACTAT | 16 |
| Bbr_0038-39 | forward | rho-indep | medium | 41 | 77 | GTGCGA | TACGAT | 17 |
| Bbr_0052 | reverse | rho-indep | high | 124 | 25 | GCTAAG | TACACT | 17 |
| Bbr_0057 | reverse | rho-indep | low | 38 | 23 | TTGTTC | TACCAT | 18 |
| Bbr_0058 | forward | rho-indep | medium | 54 | 47 | GTGAGC | TATTAG | 18 |
| Bbr_0060 | forward | loop | medium | 108 | 235 | GTGATA | TATGCA | 17 |
| Bbr_0061 | reverse | loop | medium | 61 | 28 | TCCCCA | TAGAAT | 23 |
| Bbr_0063 | reverse | loop | medium | 32 | 24 | TTTACT | TATTAT | 18 |
| Bbr_0064-66 | forward | rho-indep | medium | 51 | 9 | TCTCCA | CATAAT | 17 |
| Bbr_0067-73 | reverse | rho-indep | medium | 25 | 24 | TTTACC | TAGGAT | 21 |
| Bbr_0081-82 | forward | nd | medium | 9 | 50.5 | CGTACC | TGTAAC | 17 |
| Bbr_0089 | forward | rho-indep | low | 95 | 64 | TTCAAC | ATTAAC | 21 |
| Bbr_0090 | forward | rho-indep | low | 168 | 11 | ATGTCT | AATAAT | 14 |
| Bbr_0091 | forward | rho-indep | low | 7 | 75 | TTGCAA | TAACCT | 15 |
| Bbr_0092 | forward | loop | low | 26 | 19 | TTCACC | AATATT | 19 |
| Bbr_0095-96 | forward | loop | medium | 50 | 21 | GTGAGA | TAGAGG | 15 |
| Bbr_0104 | forward | rho-indep | low | 74 | 37 | AAGCCC | CAGAAT | 16 |
| Bbr_0105 | forward | rho-indep | low | 33 | 36 | GGGAGA | TACAAT | 17 |
| Bbr_0116 | reverse | rho-indep | low | 116 | 284 | TTGCAA | TACGGT | 17 |
| Bbr_0118 | forward | rho-indep | low | 133 | 21 | ATGCTA | AGCAAT | 16 |
| Bbr_0124-25 | forward | rho-indep | medium | 84 | 71 | TTGCCA | TGTAGG | 16 |
| Bbr_0126-27 | forward | rho-indep | low | 22 | 40 | TCGCCA | TAAAGA | 14 |
| Bbr_0130-31 | forward | rho-indep | low | 53 | 33 | TTGTCG | TGGAAT | 16 |
| Bbr_0147-48 | forward | rho-indep | low | 58 | 30 | TTCGCT | TATAAT | 19 |
| Bbr_0149 | reverse | rho-indep | low | 79 | 23 | CGGCCA | TACAAT | 20 |
| Bbr_0150 | forward | loop | low | 82 | 88 | TAGCGA | TACACT | 16 |
| Bbr_0175 | forward | rho-indep | low | 99 | 16 | TTGGGA | TGGAAG | 14 |
| Bbr_0176 | reverse | rho-indep | low | 21 | 104 | CTGAGA | TTCAAG | 20 |
| Bbr_0177-80 | forward | nd | low | 25 | 50.5 | GTGAAT | TGTAAT | 20 |
| Bbr_0181 | reverse | nd | medium | 37 | 4 | TTGTTT | TAATTT | 15 |
| Bbr_0182 | reverse | rho-indep | high | 69 | 29 | TGGATT | TATACT | 16 |
| Bbr_0183 | forward | rho-indep | high | 168 | 35 | TTGATA | TAGAAT | 17 |
| Bbr_0186-88 | forward | loop | medium | 24 | 93 | TTGAGT | TGCAAC | 17 |
| Bbr_0189 | reverse | rho-indep | medium | 27 | 209 | GTGCAT | TAGACT | 18 |
| Bbr_0190 | forward | rho-indep | medium | 13 | 57 | TGTTGA | AAAACT | 14 |
| Bbr_0191 | reverse | rho-indep | low | 145 | 123 | GTGTCG | TTAAAT | 15 |
| Bbr_0193 | forward | loop | low | 26 | 48 | TCGAGG | TACAAT | 19 |
| Bbr_0196 | forward | rho-indep | medium | 50.5 | 50.5 | GTGGCG | CACAAT | 16 |
| Bbr_0199 | reverse | loop | low | 50 | 16 | TTAGGA | TATAGT | 19 |
| Bbr_0203-05 | forward | rho-indep | medium | 27 | 44 | CGGAGA | GATAAC | 20 |
| Bbr_0208 | forward | nd | low | 145 | 50.5 | ATGTCT | CTCAAT | 15 |
| Bbr_0212-13 | reverse | rho-indep | low | 43 | 137 | TCGTCG | TACAAT | 16 |
| Bbr_0214 | reverse | loop | low | 63 | 8 | AGCACA | TATCCT | 19 |
| Bbr_0215-16 | reverse | loop | low | 50.5 | 50.5 | TCTACA | ACTAAT | 16 |
| Bbr_0221-22 | forward | rho-indep | low | 35 | 26 | TAGAGT | TATTGA | 18 |
| Bbr_0231-32 | forward | loop | medium | 57 | 161 | GTGTCA | CCTAAG | 15 |
| Bbr_0233-35 | reverse | loop | low | 55 | 6 | TAGATA | AATAAT | 19 |
| Bbr_0238-39 | reverse | loop | low | 8 | 10 | TCCCCG | TAAGCT | 18 |
| Bbr_0240 | forward | rho-indep | low | 43 | 52 | GTGCCA | TGGAAT | 24 |
| Bbr_0241-42 | forward | rho-indep | medium | 104 | 47 | ACGAAA | TTTAAC | 16 |
| Bbr_0248-49 | forward | loop | medium | 50.5 | 50.5 | CCGAAA | TACATT | 17 |
| Bbr_0254-57 | forward | rho-indep | high | 109 | 58 | GCGGCA | TAGGAT | 17 |
| Bbr_0263-65 | forward | nd | medium | 26 | 1146 | TCCGCA | TATCAT | 19 |
| Bbr_0267 | forward | rho-indep | low | 87 | 27 | TCGCCA | AACAAT | 21 |
| Bbr_0276-78 | forward | rho-indep | medium | 7 | 12 | CTGCGT | TATAGT | 15 |
| Bbr_0279-81 | forward | rho-indep | medium | 39 | 187 | CGTTCA | TATGTT | 15 |
| Bbr_0282 | forward | loop | medium | 116 | 4 | TTTGCA | TAGAGT | 18 |
| Bbr_0287-88 | forward | loop | low | 57 | 50.5 | CTCACA | TATACA | 20 |
| Bbr_0289-94 | reverse | rho-indep | medium | 75 | 37 | GCTACA | TTCAAC | 15 |
| Bbr_0295 | reverse | rho-indep | high | 35 | 97 | TAGCCG | TAGTGT | 17 |
| Bbr_0296 | reverse | loop | low | 37 | 10 | TTGCAG | CACAAT | 20 |
| Bbr_0323-30 | forward | rho-indep | high | 157 | 23 | TTGACT | AATAAT | 17 |
| Bbr_0332 | reverse | loop | low | 27 | 16 | CTGTCT | GAAAAT | 16 |
| Bbr_0333-35 | forward | loop | medium | 30 | 50.5 | GGTAAT | TAGAAT | 16 |
| Bbr_0338-41 | forward | rho-indep | low | 42 | 19 | TCCGTA | TAAACT | 17 |
| Bbr_0370-71 | forward | rho-indep | high | 56 | 40 | TTGGCG | TAGAGA | 16 |
| Bbr_0376a | forward | nd | medium | 133 | 30 | TTGACG | AAACAT | 16 |
| Bbr_0377 | forward | rho-indep | low | 61 | 82 | GTGAAA | TACGCT | 14 |
| Bbr_0379-81 | reverse | rho-indep | high | 128 | 23 | TCACCA | TATAAT | 21 |
| Bbr_0384 | forward | rho-indep | medium | 157 | 108 | TCGAGT | GAGAAT | 15 |
| Bbr_0385 | reverse | rho-indep | low | 16 | 113 | AGGCCA | TCAAAC | 15 |
| Bbr_0386-87 | forward | loop | low | 7 | 8 | TTAACG | CACAAT | 20 |
| Bbr_0393 | reverse | rho-indep | high | 24 | 28 | TTAAGA | AATCGT | 18 |
| Bbr_0397 | forward | loop | medium | 47 | 107 | TTTACA | AATTAT | 17 |
| Bbr_0401 | forward | loop | medium | 38 | 53 | TCCCCA | TACACT | 17 |
| Bbr_0402-03 | forward | loop | medium | 33 | 53 | TAGTGA | GACAAC | 15 |
| Bbr_0405 | forward | loop | high | 72 | 226 | TTTTTA | TATTCT | 16 |
| Bbr_0408 | forward | rho-indep | high | 230 | 61 | GTTCTG | TACAAT | 14 |
| Bbr_0409-10 | forward | loop | low | 18 | 11 | CCGGCA | CATTAT | 16 |
| Bbr_0427 | forward | nd | low | 34 | 50.5 | TTGACA | TATAAC | 18 |
| Bbr_0428 | reverse | nd | low | 232 | 50.5 | TTCCGA | TATAGC | 21 |
| Bbr_0429 | forward | rho-indep | medium | 56 | 55 | TGTGCA | CATAAT | 11 |
| Bbr_0430 | reverse | nd | medium | 127 | 18 | TTATCT | TAAATT | 17 |
| Bbr_0431 | forward | rho-indep | medium | 116 | 179 | TTACCA | TAAAGT | 17 |
| Bbr_0434-41 | reverse | loop | medium | 224 | 6 | GTGTGA | GATAAT | 14 |
| Bbr_0464 | forward | rho-indep | low | 56 | 45 | TCGAAC | TAAGAT | 17 |
| Bbr_0466-68 | forward | rho-indep | medium | 86 | 80 | ATGTCA | AAATAT | 17 |
| Bbr_0473 | reverse | rho-indep | medium | 42 | 37 | TTGTAA | TACAAT | 17 |
| Bbr_0474 | forward | rho-indep | medium | 43 | 35 | TCGAGA | TAAATT | 17 |
| Bbr_0475 | forward | loop | medium | 46 | 222 | CTGGCA | GATAAA | 17 |
| Bbr_0476 | reverse | loop | low | 32 | 81 | TATTCA | TATGAT | 14 |
| Bbr_0477 | reverse | rho-indep | high | 41 | 82 | TTGCCG | GGGAAA | 17 |
| Bbr_0478-79 | forward | loop | low | 15 | 3 | GTGCCC | GATAAT | 18 |
| Bbr_0486-87 | forward | loop | medium | 43 | 44 | TCGAAT | TACAAT | 14 |
| Bbr_0489 | reverse | loop | low | 27 | 21 | TTAAAG | CACAAT | 20 |
| Bbr_0490-92 | forward | rho-indep | low | 105 | 221 | TTGGTT | GATAAT | 17 |
| Bbr_0493-94 | forward | rho-indep | medium | 40 | 86 | ATGAAC | TATACT | 21 |
| Bbr_0495 | reverse | nd | low | 50.5 | 50.5 | TTAGCA | CAGAAT | 21 |
| Bbr_0498-99 | forward | rho-indep | medium | 138 | 60 | GTCGCG | TATAGC | 15 |
| Bbr_0527-29 | forward | loop | low | 47 | 24 | TGGACA | TATGAT | 16 |
| Bbr_0530 | forward | nd | medium | 159 | 28 | TTGCGA | TATACT | 16 |
| Bbr_0532 | reverse | nd | low | 36 | 31 | AGCCCA | TAGAAT | 17 |
| Bbr_0533-37 | forward | nd | medium | 66 | 22 | GTGCAA | TATGAT | 17 |
| Bbr_0541 | forward | rho-indep | medium | 26 | 21 | TTGCTA | GAACAT | 20 |
| Bbr_0543 | reverse | rho-indep | low | 117 | 31 | CTGTTT | ACTAAT | 17 |
| Bbr_0553 | reverse | nd | medium | 107 | 50.5 | TTAAGG | TGAAAT | 16 |
| Bbr_0562 | forward | nd | low | 75 | 50.5 | ATGCGG | TATACA | 15 |
| Bbr_0565 | reverse | nd | low | 32 | 50.5 | TTGCCG | TCGATT | 19 |
| Bbr_0567-69 | forward | rho-indep | medium | 17 | 6 | GCGCCC | TATTCT | 16 |
| Bbr_0579-81 | forward | loop | medium | 81 | 66 | ATGATA | TATTCT | 18 |
| Bbr_0582-84 | reverse | rho-indep | medium | 26 | 215 | TTGGTA | GTTAAT | 13 |
| Bbr_0597-99 | reverse | rho-indep | medium | 17 | 34 | TTACCG | AATAAT | 17 |
| Bbr_0604-07 | forward | rho-indep | high | 240 | 45 | TTGTCC | GATATT | 17 |
| Bbr_0631-36 | forward | loop | high | 59 | 50.5 | TTCACT | CATAAT | 19 |
| Bbr_0641 | forward | rho-indep | low | 62 | 38 | TTCAAT | TATGGT | 17 |
| Bbr_0642 | forward | rho-indep | high | 66 | 451 | CAGACG | TATGAT | 15 |
| Bbr_0645-46 | reverse | nd | low | 98 | 3 | TTCAGA | AACAAT | 15 |
| Bbr_0647 | forward | loop | low | 28 | 3 | TGTTCA | TATTGT | 20 |
| Bbr_0648 | reverse | loop | low | 31 | 33 | TTCCTA | GTTAAT | 16 |
| Bbr_0649 | forward | rho-indep | low | 50 | 6 | TTGTCA | GTTTTG | 19 |
| Bbr_0650-51 | reverse | loop | medium | 30 | 30 | ACGTCA | TATTAG | 16 |
| Bbr_0653 | reverse | nd | medium | 33 | 50.5 | TCCAGC | TACACT | 20 |
| Bbr_0654-55 | forward | nd | medium | 52 | 12 | GCGAGA | TATTTG | 15 |
| Bbr_0656-61 | forward | loop | medium | 41 | 27 | TACACG | TACAGT | 20 |
| Bbr_0663 | reverse | rho-indep | medium | 168 | 16 | TTGCGC | TATACT | 15 |
| Bbr_0667-69 | forward | rho-indep | low | 76 | 428 | TAGGCT | GAGAAT | 19 |
| Bbr_0670 | reverse | rho-indep | medium | 21 | 20 | GCGCGA | TATATT | 18 |
| Bbr_0671-72 | reverse | rho-indep | medium | 32 | 26 | ATGGCT | CATACT | 15 |
| Bbr_0673 | reverse | nd | medium | 93 | 12 | GTCACC | TGGAAT | 19 |
| Bbr_0674 | forward | rho-indep | low | 91 | 38 | TTGTTT | TGTCAT | 15 |
| Bbr_0675-87/88 | forward | nd | medium | 173 | 50.5 | TTGCCT | TATAGT | 17 |
| Bbr_0688 | forward | rho-indep | medium | 42 | 126 | TGCATA | TATACT | 18 |
| Bbr_0692 | forward | rho-indep | low | 47 | 98 | TCCACC | TAGTCT | 20 |
| Bbr_0700-03 | forward | loop | medium | 30 | 50.5 | TTGACT | TTTTAT | 16 |
| Bbr_0715 | reverse | rho-indep | low | 47 | 39 | GCGACG | TAGAAT | 18 |
| Bbr_0716-18 | forward | nd | medium | 46 | 50.5 | CTGCGT | GTTAAG | 15 |
| Bbr_0720-24 | forward | loop | medium | 81 | 50.5 | TTCGCT | TTTAAT | 17 |
| Bbr_0725 | forward | rho-indep | high | 43 | 44 | TTCACA | TACTCT | 17 |
| Bbr_0730-32 | forward | nd | low | 29 | 33 | ATGCTG | TATACG | 18 |
| Bbr_0734 | forward | rho-indep | low | 52 | 17 | TGGACA | TATTCT | 18 |
| Bbr_0735 | reverse | rho-indep | medium | 55 | 38 | TTGAAA | TACCTT | 18 |
| Bbr_0738-40 | forward | nd | medium | 39 | 50.5 | TTGACA | TGTAAT | 18 |
| Bbr_0744-46 | reverse | loop | medium | 52 | 27 | TAGCGT | TAGACT | 18 |
| Bbr_0747-48 | forward | loop | medium | 40 | 14 | GTCCCA | AATTAT | 18 |
| Bbr_0753 | forward | rho-indep | high | 77 | 36 | TGGGCA | TAAATA | 15 |
| Bbr_0757 | forward | rho-indep | medium | 63 | 41 | TTGTCA | TAGACT | 18 |
| Bbr_0758 | reverse | rho-indep | medium | 39 | 60 | TCGTCA | TACAAT | 17 |
| Bbr_0771 | reverse | rho-indep | high | 56 | 42 | TTGTAG | TCTAAT | 14 |
| Bbr_0772 | reverse | loop | high | 45 | 50.5 | GTGAAC | TACAAT | 15 |
| Bbr_0773 | reverse | loop | medium | 35 | 8 | TTTACG | TATGCT | 15 |
| Bbr_0776 | forward | rho-indep | high | 106 | 37 | TTCAAA | TACCAT | 16 |
| Bbr_0777-79 | forward | loop | medium | 64 | 25 | CCGACA | TATTGT | 14 |
| Bbr_0787 | forward | rho-indep | medium | 48 | 37 | TTCTCA | GTGAAG | 20 |
| Bbr_0791 | forward | nd | medium | 23 | 32 | TTGCCT | GAAAAT | 16 |
| Bbr_0794-95 | forward | rho-indep | medium | 28 | 57 | TTCAGT | AACAAT | 20 |
| Bbr_0802 | reverse | nd | low | 30 | 50.5 | ATGACG | AAGAAT | 20 |
| Bbr_0806 | reverse | loop | low | 32 | 40 | CCTACA | GATTAT | 16 |
| Bbr_0814-17 | reverse | rho-indep | low | 109 | 103 | TTGGCG | TATCCT | 21 |
| Bbr_0818 | forward | loop | low | 62 | 32 | CGGATA | CATATT | 18 |
| Bbr_0819-20 | reverse | nd | low | 35 | 50.5 | TTGTTT | TATAAT | 17 |
| Bbr_0857 | reverse | loop | low | 44 | 24 | TTCCGA | TATCAT | 18 |
| Bbr_0859-62 | forward | rho-indep | medium | 97 | 50.5 | GTGTCC | TATATC | 17 |
| Bbr_0863 | forward | loop | medium | 71 | 50.5 | GTGAAA | CACAAG | 19 |
| Bbr_0865-66 | forward | nd | low | 35 | 50.5 | CGGAAA | TACAAT | 20 |
| Bbr_0882-83 | reverse | rho-indep | low | 36 | 38 | CGGACA | TATCAT | 21 |
| Bbr_0888 | forward | nd | high | 190 | 76 | CTGTCC | TATTGT | 15 |
| Bbr_0889-90 | reverse | loop | low | 53 | 229 | AGGATA | AGTAAT | 19 |
| Bbr_0891-92 | forward | loop | low | 29 | 60 | TGGGCA | TACTAG | 17 |
| Bbr_0893-94 | reverse | loop | low | 35 | 96 | CTGACA | TATGAT | 14 |
| Bbr_0895-00 | forward | loop | low | 7 | 55 | TTCCTG | TAGCAT | 17 |
| Bbr_0902-12 | forward | rho-indep | medium | 38 | 82 | GTGAAC | TAACAT | 15 |
| Bbr_0913 | reverse | rho-indep | medium | 83 | 22 | TTGCGT | AATTAT | 16 |
| Bbr_0915 | forward | rho-indep | low | 40 | 50.5 | TTAGCA | TGCAAT | 14 |
| Bbr_0916-20 | forward | nd | low | 60 | 28 | TTGGCT | TGCAAT | 13 |
| Bbr_0921 | reverse | rho-indep | high | 62 | 22 | GTGACG | TACAAT | 17 |
| Bbr_0922-24 | forward | rho-indep | medium | 50 | 37 | CCCATA | TACAAT | 16 |
| Bbr_0925 | reverse | rho-indep | low | 62 | 28 | TGTCCA | CACTCT | 17 |
| Bbr_0926 | forward | rho-indep | high | 137 | 47 | CCGCCA | TATAAT | 16 |
| Bbr_0930 | reverse | rho-indep | high | 44 | 25 | TTGCAC | TATCCT | 21 |
| Bbr_0931-32 | forward | loop | low | 37 | 45 | ATGCCA | TACACT | 21 |
| Bbr_0936-37 | forward | rho-indep | low | 104 | 700 | TTGACT | TATATG | 15 |
| Bbr_0939-40 | forward | rho-indep | low | 46 | 59 | TGCATA | TATGAT | 20 |
| Bbr_0942-47 | forward | nd | medium | 41 | 50.5 | TTGTAT | AAGAAG | 15 |
| Bbr_0948 | reverse | nd | low | 57 | 50.5 | TCGGCG | CATATT | 18 |
| Bbr_0955 | forward | loop | low | 48 | 145 | TAGTAT | TATCTT | 16 |
| Bbr_0967-69 | forward | loop | low | 39 | 10 | CTTACA | TAGACT | 17 |
| Bbr_0971-72 | forward | rho-indep | medium | 40 | 113 | GTTCCC | TATCAT | 15 |
| Bbr_0973-79 | forward | rho-indep | low | 48 | 23 | CTGTCC | TAAATT | 16 |
| Bbr_0981-83 | reverse | loop | low | 35 | 84 | ATGACT | ATCAAT | 16 |
| Bbr_0984-85 | forward | rho-indep | low | 47 | 14 | CGTCCA | TACTAT | 19 |
| Bbr_0988 | reverse | rho-indep | low | 69 | 25 | GCGGCA | CATGGT | 17 |
| Bbr_0989-90 | forward | nd | low | 65 | 50 | TCGAAT | TATTCT | 17 |
| Bbr_0991-92 | forward | nd | low | 10 | 50.5 | ATGTCG | TATTCA | 17 |
| Bbr_0993 | forward | rho-indep | low | 81 | 46 | TTCGGT | TATCAC | 16 |
| Bbr_0994-96 | forward | rho-indep | medium | 40 | 18 | ATGCCT | AAAAAT | 17 |
| Bbr_0997-99 | forward | rho-indep | low | 84 | 10 | TTTTCA | TATACC | 14 |
| Bbr_1002-03 | reverse | rho-indep | high | 99 | 47 | CTACCA | CATAAC | 19 |
| Bbr_1004-05 | forward | loop | low | 47 | 52 | TTAGCA | TAATAC | 18 |
| Bbr_1006 | reverse | loop | low | 40 | 45 | TTGGTA | ATGAAT | 18 |
| Bbr_1007 | forward | loop | medium | 37 | 91 | GTTCTA | TTCAAC | 19 |
| Bbr_1009-20 | forward | nd | medium | 69 | 265 | TTGCCA | TATCGT | 18 |
| Bbr_1021 | reverse | loop | low | 57 | 55 | ATCATA | TAGACT | 20 |
| Bbr_1022-23 | forward | loop | low | 94 | 7 | TAGTCC | TATATA | 15 |
| Bbr_1025-31 | forward | loop | low | 14 | 11 | GTTACA | TATGAC | 14 |
| Bbr_1033-42 | reverse | rho-indep | medium | 48 | 24 | GTGAGG | TATATC | 16 |
| Bbr_1043-44 | reverse | rho-indep | low | 53 | 154 | TAGCCC | AATAAG | 21 |
| Bbr_1046 | reverse | nd | low | 38 | 50.5 | CTGCAC | TATGAT | 16 |
| Bbr_1047 | forward | loop | low | 89 | 50 | GTGAGC | TATCAT | 15 |
| Bbr_1050-52 | reverse | loop | low | 113 | 23 | TTGTCA | TGTATT | 21 |
| Bbr_1055 | reverse | nd | medium | 100 | 50.5 | GTGTCA | ACCAAT | 14 |
| Bbr_1056 | reverse | loop | low | 26 | 27 | TTGCGC | TTGAAT | 17 |
| Bbr_1066-73 | forward | nd | low | 84 | 50.5 | TAAACA | TACCAT | 18 |
| Bbr_1074-78 | reverse | nd | low | 156 | 50.5 | TTGCCC | GCGAAT | 14 |
| Bbr_1079-83 | reverse | rho-indep | low | 47 | 97 | TTGTCT | CATAAT | 18 |
| Bbr_1084-85 | forward | nd | low | 39 | 50.5 | TTGATT | TATACT | 16 |
| Bbr_1090 | forward | loop | low | 55 | 163 | GCGCAA | TATACT | 18 |
| Bbr_1091 | reverse | loop | low | 123 | 27 | TTGACG | TATAAT | 21 |
| Bbr_1094 | forward | nd | low | 41 | 50.5 | TCCCCA | GTAAAT | 17 |
| Bbr_1095-98 | reverse | nd | low | 88 | 50.5 | TTCCCA | TAGTCT | 16 |
| Bbr_1100-03 | reverse | nd | medium | 134 | 50.5 | CTGCAA | TACGCT | 20 |
| Bbr_1104-05 | reverse | rho-indep | high | 201 | 42 | AGCATA | TATTCT | 17 |
| Bbr_1106-07 | reverse | rho-indep | medium | 58 | 90 | TAGTCA | TATAGT | 16 |
| Bbr_1109 | reverse | nd | medium | 43 | 45 | GTGGAA | TGAAAT | 17 |
| Bbr_1110 | forward | nd | low | 29 | 36 | GTTACA | TAAAAC | 15 |
| Bbr_1111 | forward | nd | low | 42 | 50.5 | GGGACT | TAGAAT | 19 |
| Bbr_1118-21 | reverse | loop | low | 7 | 76 | GTGATT | TATAAT | 16 |
| Bbr_1130-32 | reverse | rho-indep | medium | 40 | 58 | AGGCCA | AATAAC | 15 |
| Bbr_1135 | reverse | rho-indep | high | 47 | 62 | GTGCCG | TATCCT | 17 |
| Bbr_1141 | reverse | rho-indep | low | 128 | 63 | CTGTAT | TATACT | 17 |
| Bbr_1156 | forward | rho-indep | medium | 20 | 21 | AGCTCA | CTCAAG | 17 |
| Bbr_1157-59 | reverse | rho-indep | medium | 62 | 49 | TTTACA | TATCCT | 16 |
| Bbr_1160 | forward | nd | low | 40 | 50.5 | TTGTAA | CATACT | 16 |
| Bbr_1162 | forward | nd | low | 24 | 13 | GCGCCG | TAATAT | 16 |
| Bbr_1168-71 | reverse | nd | medium | 19 | 13 | TCTACT | GATAAG | 18 |
| Bbr_1173-74 | forward | rho-indep | low | 20 | 9 | TTTTCA | CATAAC | 16 |
| Bbr_1175 | reverse | nd | medium | 57 | 11 | TTGCTA | CATAAC | 14 |
| Bbr_1176 | forward | rho-indep | medium | 26 | 46 | ATTCCT | TATTGG | 17 |
| Bbr_1177 | reverse | rho-indep | low | 50 | 17 | TTCAAG | TACAAT | 17 |
| Bbr_1178 | reverse | rho-indep | medium | 42 | 27 | ATGTCT | TATTCT | 15 |
| Bbr_1179 | reverse | nd | high | 73 | 37 | CCGGCT | TACAAT | 18 |
| Bbr_1185 | reverse | rho-indep | medium | 36 | 30 | TCCAGC | TAGAAG | 15 |
| Bbr_1188-89 | reverse | nd | low | 23 | 50.5 | CGCATC | TAGAAT | 17 |
| Bbr_1190 | forward | nd | low | 7 | 50.5 | CTCCCA | TATATT | 21 |
| Bbr_1192 | reverse | rho-indep | medium | 30 | 34 | TTCATG | TAGTAT | 21 |
| Bbr_1195-00 | reverse | rho-indep | medium | 51 | 48 | GCGGCC | TCTAAG | 16 |
| Bbr_1202-04 | reverse | nd | high | 74 | 23 | TTTACA | TAAACT | 18 |
| Bbr_1205 | reverse | rho-indep | high | 134 | 44 | TGGTTG | AAAAAT | 14 |
| Bbr_1213 | reverse | loop | medium | 104 | 2 | TTGTGT | TAACAT | 15 |
| Bbr_1228-30 | reverse | loop | high | 153 | 15 | GTCAAT | TAGACT | 14 |
| Bbr_1233-34 | forward | loop | medium | 69 | 50.5 | TTGCCA | TACAGT | 18 |
| Bbr_1236 | forward | rho-indep | medium | 57 | 21 | CTTCGA | TAGACT | 14 |
| Bbr_1237 | forward | rho-indep | medium | 41 | 208 | ACGCCG | TACGAT | 19 |
| Bbr_1238-39 | reverse | rho-indep | low | 27 | 27 | CTCACA | TATGGG | 15 |
| Bbr_1240-42 | forward | nd | low | 33 | 50.5 | ACGCCA | TAGAAT | 16 |
| Bbr_1244 | reverse | nd | medium | 228 | 50.5 | GTTTCA | TAAACT | 14 |
| Bbr_1245-46 | forward | rho-indep | medium | 49 | 22 | ATGCCA | TAGTAT | 18 |
| Bbr_1247-48 | reverse | rho-indep | low | 148 | 37 | GTGTCC | TAGTAT | 19 |
| Bbr_1254 | reverse | rho-indep | low | 50.5 | 50.5 | ATGAGA | TATCTG | 16 |
| Bbr_1255-66 | reverse | rho-indep | low | 51 | 536 | GGGAAA | TAAAGT | 17 |
| Bbr_1267-68 | forward | nd | low | 48 | 34 | TTCCCC | TAAATT | 18 |
| Bbr_1269-70 | reverse | loop | medium | 46 | 20 | TTGACG | CACTCT | 17 |
| Bbr_1271 | forward | rho-indep | low | 26 | 57 | TTGATT | TATGCT | 17 |
| Bbr_1273 | reverse | rho-indep | high | 47 | 118 | TTTATA | TACAAG | 16 |
| Bbr_1275-76 | forward | nd | low | 44 | 50.5 | TTATCA | CAGCAT | 18 |
| Bbr_1278 | reverse | rho-indep | medium | 36 | 25 | GTAACA | GTTAAC | 17 |
| Bbr_1280-85 | reverse | rho-indep | low | 31 | 12 | GTTCCT | TACGTT | 16 |
| Bbr_1287 | forward | rho-indep | medium | 33 | 27 | CAGCCA | TAAAAT | 17 |
| Bbr_1293 | reverse | loop | medium | 47 | 7 | TGTCCG | TATCTG | 17 |
| Bbr_1294-96 | reverse | loop | low | 22 | 24 | CTCGCC | GACAAG | 17 |
| Bbr_1398-01 | reverse | nd | medium | 76 | 50.5 | TTCTCA | CATGTT | 22 |
| Bbr_1302-03 | reverse | rho-indep | medium | 46 | 32 | TCGCGT | TATCCT | 17 |
| Bbr_1304-10 | reverse | nd | low | 70 | 118 | TTGACA | CATAAT | 15 |
| Bbr_1311-12 | forward | nd | medium | 124 | 50.5 | ACGACA | GATAAT | 21 |
| Bbr_1313 | reverse | nd | low | 49 | 50.5 | TTGATG | TGAGAT | 17 |
| Bbr_1314-15 | forward | nd | low | 23 | 163 | AGGAAA | CATCAT | 14 |
| Bbr_1325 | reverse | rho-indep | medium | 91 | 30 | GCAACA | CACAAT | 16 |
| Bbr_1327 | reverse | nd | medium | 60 | 50.5 | TTCACT | AGCAAT | 18 |
| Bbr_1328-30 | forward | nd | low | 164 | 22 | TTGCAC | TATGAA | 17 |
| Bbr_1334 | forward | nd | low | 130 | 50.5 | GTGCAA | TACACT | 20 |
| Bbr_1348-49 | reverse | nd | medium | 138 | 130 | TTGACG | TATATT | 14 |
| Bbr_1350 | forward | loop | medium | 85 | 13 | TGCACC | TATAAT | 17 |
| Bbr_1356 | reverse | rho-indep | high | 31 | 120 | TAAACG | TAGAGT | 14 |
| Bbr_1357 | forward | loop | low | 43 | 33 | GTGAGA | TATGCT | 15 |
| Bbr_1358-59 | reverse | loop | medium | 29 | 73 | GTGAGG | CATTAT | 18 |
| Bbr_1364 | reverse | rho-indep | high | 118 | 33 | TTGGCA | GCTAAT | 16 |
| Bbr_1365 | reverse | rho-indep | medium | 100 | 29 | TTTGTA | TACAAT | 17 |
| Bbr_1366 | reverse | rho-indep | low | 34 | 23 | GTGGCT | TACACT | 18 |
| Bbr_1376 | reverse | nd | medium | 89 | 50.5 | TCCAGC | TATACT | 15 |
| Bbr_1379 | forward | nd | high | 53 | 50 | TTTTCG | TATCAT | 16 |
| Bbr_1397 | reverse | rho-indep | low | 67 | 22 | ATGACT | AAAAAT | 14 |
| Bbr_1398-99 | forward | rho-indep | medium | 42 | 27 | TTGGAA | TATACT | 18 |
| Bbr_1414 | reverse | rho-indep | medium | 343 | 164 | TGCAGA | TAGGAT | 18 |
| Bbr_1451 | reverse | rho-indep | medium | 164 | 101 | TGCAGC | TATCCT | 18 |
| Bbr_1455-56 | reverse | nd | low | 78 | 50.5 | TTGTCC | CAAACT | 17 |
| Bbr_1461 | forward | rho-indep | low | 51 | 38 | ATGACG | CAAAAC | 16 |
| Bbr_1463 | reverse | rho-indep | high | 34 | 54 | TGGCAG | TAGAGT | 17 |
| Bbr_1464 | reverse | rho-indep | high | 50 | 21 | TTGATG | TAGCCT | 17 |
| Bbr_1465 | forward | rho-indep | low | 185 | 38 | TTCTCC | TTGAAG | 16 |
| Bbr_1467 | reverse | rho-indep | low | 25 | 27 | TTCACG | TATGAC | 16 |
| Bbr_1472 | reverse | rho-indep | medium | 96 | 52 | GTGAAG | TATAGT | 15 |
| Bbr_1482 | reverse | rho-indep | high | 73 | 46 | TTCCCC | TACAAT | 15 |
| Bbr_1488 | reverse | rho-indep | medium | 40 | 165 | GTGGGA | TATTTT | 19 |
| Bbr_1504 | forward | loop | low | 94 | 47 | CTGCGG | TATTGT | 16 |
| Bbr_1505 | reverse | rho-indep | medium | 83 | 46 | TTCAGC | TAACAT | 18 |
| Bbr_1506 | forward | rho-indep | low | 55 | 50.5 | ATGACT | TAGAAG | 20 |
| Bbr_1554 | reverse | rho-indep | medium | 67 | 51 | TTGACA | CATAAT | 17 |
| Bbr_1558-60 | forward | rho-indep | low | 61 | 34 | GTGCAT | ACTAAC | 18 |
| Bbr_1562-64 | reverse | loop | low | 86 | 8 | TTTATG | TCTAAG | 19 |
| Bbr_1565 | forward | rho-indep | medium | 104 | 110 | TACGCA | CATAAT | 17 |
| Bbr_1566 | reverse | rho-indep | medium | 47 | 15 | TTGCCA | TATTGT | 21 |
| Bbr_1567 | reverse | nd | medium | 31 | 19 | TTGATG | AAGATT | 16 |
| Bbr_1568-69 | reverse | loop | medium | 69 | 18 | TGGGCA | TACAGT | 17 |
| Bbr_1573 | forward | rho-indep | medium | 45 | 46 | TTGAAG | AATAAT | 17 |
| Bbr_1574 | reverse | rho-indep | medium | 7 | 29 | GTGTTT | TAGACT | 15 |
| Bbr_1576 | reverse | loop | medium | 92 | 50.5 | TTCTTA | TACACT | 15 |
| Bbr_1580-82 | reverse | nd | medium | 21 | 50.5 | TTGTGG | TGTAAT | 14 |
| Bbr_1583-84 | forward | nd | low | 42 | 7 | GTTCCA | CACAAT | 15 |
| Bbr_1585 | reverse | nd | medium | 48 | 91 | GTTCGA | GATAAA | 16 |
| Bbr_1587-90 | reverse | rho-indep | medium | 54 | 120 | TTGACA | TAAACT | 17 |
| Bbr_1591 | reverse | loop | low | 43 | 50.5 | ATGAGA | TATTAT | 17 |
| Bbr_1592 | forward | nd | low | 37 | 50.5 | GCGAAA | AACAAT | 17 |
| Bbr_1594 | reverse | nd | medium | 214 | 50.5 | TTGCCG | TATAAT | 17 |
| Bbr_1595 | forward | rho-indep | medium | 38 | 33 | TTCGCA | CACAAT | 20 |
| Bbr_1600 | forward | rho-indep | medium | 59 | 28 | GTCACA | GATAAG | 20 |
| Bbr_1601-02 | reverse | rho-indep | low | 48 | 49 | CTGTGA | ACTAAT | 16 |
| Bbr_1607 | reverse | rho-indep | medium | 154 | 79 | GTGCTA | TATGCT | 14 |
| Bbr_1608 | reverse | loop | low | 61 | 57 | GAGCGT | TACACT | 15 |
| Bbr_1614-19 | reverse | rho-indep | high | 35 | 31 | TTGACA | TATGCC | 17 |
| Bbr_1620-21 | reverse | rho-indep | medium | 57 | 32 | TTGTCT | TATGCT | 18 |
| Bbr_1622-42 | reverse | loop | high | 210 | 29 | TCCAGT | TAGATA | 16 |
| Bbr_1645 | reverse | loop | medium | 151 | 72 | ATGCAG | TATATT | 17 |
| Bbr_1648-49 | reverse | rho-indep | high | 172 | 32 | TTGCCC | TATACT | 17 |
| Bbr_1667 | reverse | rho-indep | high | 28 | 23 | GCGCCC | ACTAAA | 18 |
| Bbr_1668 | reverse | nd | high | 96 | 25 | TTGGCA | TACGAT | 18 |
| Bbr_1673-74 | reverse | loop | low | 28 | 30 | TGCGCA | GATAAT | 21 |
| Bbr_1675-76 | reverse | nd | high | 212 | 42 | TGTCGC | AATCAT | 16 |
| Bbr_1678 | forward | rho-indep | medium | 141 | 69 | TGTTCA | TATAAT | 16 |
| Bbr_1700-01 | reverse | loop | low | 44 | 50.5 | GCTTCA | TACAGT | 16 |
| Bbr_1709 | reverse | rho-indep | high | 87 | 21 | TTGCGG | TATATT | 17 |
| Bbr_1710 | forward | rho-indep | medium | 106 | 13 | TTCCCC | TATAAT | 16 |
| Bbr_1722 | forward | nd | low | 231 | 50.5 | ATCGCA | TACGAT | 16 |
| Bbr_1723 | reverse | nd | low | 18 | 50.5 | GAGACA | GCAAAT | 16 |
| Bbr_1726-27 | reverse | rho-indep | high | 185 | 28 | TTGTTG | CATAAT | 14 |
| Bbr_1729-30 | reverse | rho-indep | high | 9 | 17 | TTTCTT | GATGCG | 16 |
| Bbr_1731 | reverse | rho-indep | low | 32 | 76 | TTGAGA | AAACAT | 16 |
| Bbr_1735-36 | reverse | rho-indep | high | 107 | 24 | GTGTCG | TAAAAA | 15 |
| Bbr_1749-51 | reverse | nd | low | 56 | 50.5 | GTGTCG | TAGTAA | 16 |
| Bbr_1753-54 | reverse | loop | low | 115 | 8 | GTCGCC | AAGAAT | 17 |
| Bbr_1757 | reverse | nd | medium | 48 | 50.5 | TTCACG | TATGGC | 16 |
| Bbr_1769 | reverse | rho-indep | low | 199 | 14 | TTCAAC | GTGAAT | 18 |
| Bbr_1770 | forward | loop | low | 31 | 47 | CTGAAT | TAGCTT | 14 |
| Bbr_1777 | forward | loop | medium | 20 | 144 | GGGGCA | GAGAAA | 19 |
| Bbr_1783-84 | reverse | nd | low | 50 | 50.5 | TTCGTT | TAGAAT | 17 |
| Bbr_1792-97 | reverse | nd | medium | 44 | 50.5 | TTGAAT | TATGTT | 19 |
| Bbr_1798-00 | reverse | rho-indep | medium | 75 | 35 | GCGACT | TCTAAG | 16 |
| Bbr_1801 | forward | nd | low | 40 | 50.5 | TCGCTT | CTTAAG | 17 |
| Bbr_1802-03 | reverse | nd | low | 162 | 50.5 | AGGAAT | TACACT | 18 |
| Bbr_1804 | forward | rho-indep | low | 86 | 17 | TTCCCA | TATAAC | 15 |
| Bbr_1808 | reverse | loop | medium | 75 | 57 | TTGGAA | AAGAAT | 15 |
| Bbr_1818 | forward | rho-indep | low | 93 | 11 | CTGAGC | TATACT | 17 |
| Bbr_1820 | reverse | rho-indep | low | 119 | 60 | GTGCCC | GATAAT | 20 |
| Bbr_1842 | forward | nd | low | 65 | 50.5 | CGGTCT | TACAAT | 17 |
| Bbr_1843-45 | reverse | nd | medium | 125 | 50.5 | TTGACA | TACCAT | 17 |
| Bbr_1847 | reverse | rho-indep | high | 88 | 126 | TTGCAA | CACAAT | 17 |
| Bbr_1854 | reverse | rho-indep | low | 26 | 105 | GTCATT | TACAAT | 18 |
| Bbr_1871 | forward | rho-indep | low | 28 | 5 | TTAATG | TATACT | 18 |
| Bbr_1872-74 | reverse | rho-indep | low | 195 | 5 | TAGACA | TATAAA | 16 |
| Bbr_1875-76 | forward | rho-indep | low | 37 | 23 | CTAATA | GCTAGA | 17 |
| Bbr_1877 | reverse | nd | low | 50 | 29 | CGCAGT | GACAAC | 18 |
| Bbr_1882 | forward | rho-indep | medium | 7 | 22 | CCGCGT | GACAAT | 18 |
| Bbr_1890 | reverse | rho-indep | high | 175 | 81 | TTGCCT | TATAGT | 16 |
| Bbr_1892-94 | reverse | rho-indep | high | 54 | 45 | GTGAAG | TTAAAT | 17 |
| Bbr_1915-18 | forward | rho-indep | low | 22 | 36 | ACGGGG | TATGAT | 17 |
| Bbr_1919-26 | reverse | rho-indep | high | 92 | 48 | TCGAAA | TATTTT | 17 |
| rRNA1 | reverse | rho-indep | high | 281 | 17 | TTGCAT | TAAGTT | 17 |
| rRNA2 | reverse | rho-indep | high | 271 | 12 | TTGCGC | TGTAAG | 15 |
| tRNA1-2 | reverse | rho-indep | high | 32 | 7 | TTGACA | TATTAT | 16 |
| tRNA3 | reverse | rho-indep | high | 14 | 11 | TTGTGC | TGTAAG | 15 |
| tRNA4 | forward | rho-indep | high | 26 | 8 | GGCGCA | TACACT | 17 |
| tRNA5-6 | forward | rho-indep | high | 47 | 4 | TTGCGG | TACAGT | 17 |
| tRNA7 | reverse | rho-indep | high | 42 | 37 | CTTATC | TATTCG | 17 |
| tRNA8 | forward | rho-indep | high | 9 | 6 | TTGAAA | TAGTTT | 17 |
| tRNA9 | forward | rho-indep | high | 38 | 12 | TTGCGA | TATATT | 16 |
| tRNA11 | reverse | rho-indep | high | 28 | 13 | TTGACT | TATTGT | 16 |
| tRNA12 | forward | rho-indep | high | 13 | 43 | TTGGCG | TACAGT | 16 |
| tRNA13 | forward | rho-indep | high | 28 | 43 | TTGCCA | TATAAT | 16 |
| tRNA14-15 | reverse | rho-indep | high | 43 | 31 | TGGACT | TATACT | 16 |
| tRNA16 | forward | rho-indep | medium | 11 | 4 | CGGAGC | TAGACT | 17 |
| tRNA17 | reverse | rho-indep | high | 32 | 6 | TGTATA | GCTAAT | 15 |
| tRNA19 | forward | rho-indep | medium | 22 | 7 | GTCTTC | AATAAC | 16 |
| tRNA20 | forward | rho-indep | high | 25 | 31 | ATGGCG | TATGCT | 15 |
| tRNA22 | reverse | nd | high | 28 | 41 | CTTTCA | TATAAA | 18 |
| tRNA23-24 | forward | rho-indep | high | 37 | 6 | TTTGCA | CGTAAT | 16 |
| tRNA25-26 | forward | rho-indep | high | 28 | 4 | GTTGCA | AGTAAT | 15 |
| tRNA27 | reverse | rho-indep | high | 28 | 8 | TTGCAT | GCTAAG | 14 |
| tRNA28-32 | forward | rho-indep | high | 43 | 6 | TGGACG | TATAGT | 17 |
| tRNA33 | forward | rho-indep | high | 24 | 8 | TTGCCA | TAAATT | 16 |
| tRNA34-35 | reverse | rho-indep | high | 40 | 13 | TTTTCC | TATATG | 16 |
| tRNA38 | reverse | rho-indep | high | 29 | 5 | GTGTCA | TAGCAT | 16 |
| tRNA39-40 | reverse | rho-indep | high | 38 | 7 | TTTGCG | TATAAG | 15 |
| tRNA41 | forward | rho-indep | high | 32 | 29 | CGGGCG | TAAAAC | 15 |
| tRNA42 | forward | rho-indep | high | 25 | 4 | CCGAGT | CATAAT | 15 |
| tRNA43 | reverse | rho-indep | medium | 20 | 11 | TTCCCT | TATCGT | 14 |
| tRNA44 | reverse | rho-indep | high | 25 | 4 | TTGCCG | CATAAT | 16 |
| tRNA45 | reverse | rho-indep | high | 35 | 4 | TCGGCA | CATGGT | 16 |
| tRNA47-48 | reverse | rho-indep | high | 45 | 49 | TTGCGT | TATATA | 18 |
| tRNA49-51 | reverse | rho-indep | high | 30 | 8 | GTGCAT | TATCTT | 17 |
| tRNA52 | reverse | nd | high | 65 | 33 | TTAATC | TATAAC | 15 |
| tRNA53 | reverse | rho-indep | high | 39 | 2 | TTGCGT | TATTAT | 14 |
| RNAseP | forward | rho-indep | high | 18 | 2 | TCGCCA | TATAAT | 21 |
| tmRNA | reverse | rho-indep | high | 43 | 5 | TTGCAA | TATGGT | 17 |
| 4.5S RNA | forward | loop | high | 37 | 26 | CCGGCA | TAAAAA | 15 |
