# Supplementary material for: Global transcriptional landscape and promoter mapping of the gut commensal Bifidobacterium breve UCC2003
Source: BMC Genomics. 2017 Dec 28;18:991. doi: 10.1186/s12864-017-4387-x (PMC5746004; doi:10.1186/s12864-017-4387-x)
Supplement: Supplementary file 5 — Transcription of essential genes in B. breve. A .docx document containing the list of essential genes of B. breve UCC2003 with associated level of transcription as detected from our transcriptomic study. (DOCX 41 kb) [file 12864_2017_4387_MOESM5_ESM.docx]

**Table S3. Expression of essential genes in *B. breve*.**

| **Locus_tag** | **Annotation** | **Expression level** |
| --- | --- | --- |
| Bbr_0001 | Chromosomal replication initiator protein DnaA | low |
| Bbr_0002 | DNA polymerase III, beta chain | low |
| Bbr_0003 | DNA replication and repair protein recF | no |
| Bbr_0004 | Conserved hypothetical protein | no |
| Bbr_0005 | DNA gyrase subunit B | low |
| Bbr_0006 | DNA gyrase subunit A | low |
| Bbr_0007 | Conserved hypothetical membrane spanning protein | low |
| Bbr_0038 | Alkyl hydroperoxide reductase C22 protein | medium |
| Bbr_0039 | Thioredoxin reductase/Thioredoxin/Glutaredoxin family protein | no |
| Bbr_0064 | Conserved hypothetical secreted protein with DUF881 domain | medium |
| Bbr_0070 | Cell division protein FtsW | medium |
| Bbr_0071 | Protein phosphatase 2C | medium |
| Bbr_0072 | Conserved hypothetical secreted protein with FHA domain | medium |
| Bbr_0073 | Conserved hypothetical protein with FHA domain | medium |
| Bbr_0091 | Ferredoxin--NADP reductase | low |
| Bbr_0124 | Chaperone protein dnaK | medium |
| Bbr_0125 | GrpE protein | medium |
| Bbr_0141 | DNA polymerase III subunit gamma/tau | no |
| Bbr_0150 | Conserved hypothetical protein | low |
| Bbr_0178 | DNA topoisomerase I | low |
| Bbr_0179 | Thymidylate kinase | low |
| Bbr_0180 | DNA polymerase III, delta' subunit | low |
| Bbr_0182 | Phosphocarrier protein HPr | high |
| Bbr_0183 | Phosphoenolpyruvate-protein phosphotransferase | high |
| Bbr_0188 | Formate--tetrahydrofolate ligase | medium |
| Bbr_0190 | Conserved hypothetical membrane spanning protein with GtrA-like domain | medium |
| Bbr_0192 | Phosphoglycerate mutase | no |
| Bbr_0196 | Glutamyl-tRNA synthetase | medium |
| Bbr_0216 | Modification methylase | low |
| Bbr_0221 | Conserved hypothetical membrane spanning protein with iron permease FTR1 family domain | low |
| Bbr_0222 | Conserved hypothetical secreted protein, probably involved in iron uptake | low |
| Bbr_0223 | Conserved hypothetical membrane spanning protein | no |
| Bbr_0224 | Permease protein of ABC transporter system | no |
| Bbr_0225 | Permease protein of ABC transporter system | no |
| Bbr_0226 | ATP-binding protein of ABC transporter system | no |
| Bbr_0227 | Conserved hypothetical protein | no |
| Bbr_0232 | 6-phosphogluconate dehydrogenase | medium |
| Bbr_0233 | 6-phosphogluconolactonase | low |
| Bbr_0234 | glucose-6-phosphate dehydrogenase subunit | low |
| Bbr_0235 | Glucose-6-phosphate 1-dehydrogenase | low |
| Bbr_0240 | Cell division protein ftsY | low |
| Bbr_0245 | Replicative DNA helicase | no |
| Bbr_0246 | UDP-N-acetylmuramoylalanyl-D-glutamate--2, 6-diaminopimelate ligase | no |
| Bbr_0247 | CobB/CobQ-like glutamine amidotransferase domain protein | no |
| Bbr_0254 | SSU ribosomal protein S6P | high |
| Bbr_0255 | Single-strand DNA binding protein | high |
| Bbr_0256 | SSU ribosomal protein S18P | high |
| Bbr_0264 | Hypothetical protein | medium |
| Bbr_0271 | Orotate phosphoribosyltransferase | no |
| Bbr_0276 | Aspartyl/glutamyl-tRNA(Asn/Gln) amidotransferase subunit C | medium |
| Bbr_0277 | Aspartyl/glutamyl-tRNA(Asn/Gln) amidotransferase subunit A | medium |
| Bbr_0278 | Glutamyl-tRNA(Gln) amidotransferase subunit B | medium |
| Bbr_0281 | Conserved hypothetical protein, possibly FAD-containing oxidoreductase | medium |
| Bbr_0282 | Transcription termination factor rho | medium |
| Bbr_0289 | Valyl-tRNA synthetase | medium |
| Bbr_0292 | Transcriptional regulatory protein glnR | medium |
| Bbr_0293 | Conserved hypothetical membrane spanning protein | medium |
| Bbr_0295 | Inorganic pyrophosphatase | high |
| Bbr_0300 | Hypothetical secreted protein | no |
| Bbr_0319 | Phage transcriptional regulator | no |
| Bbr_0323 | ATP synthase A chain | high |
| Bbr_0324 | ATP synthase C chain | high |
| Bbr_0325 | ATP synthase B chain | high |
| Bbr_0326 | ATP synthase delta chain | high |
| Bbr_0327 | ATP synthase alpha chain | high |
| Bbr_0328 | ATP synthase gamma chain | high |
| Bbr_0329 | ATP synthase beta chain | high |
| Bbr_0330 | ATP synthase epsilon chain | high |
| Bbr_0333 | Conserved hypothetical membrane spanning protein | medium |
| Bbr_0334 | Conserved hypothetical secreted protein | medium |
| Bbr_0339 | 1-acyl-sn-glycerol-3-phosphate acyltransferase | low |
| Bbr_0340 | Glycerol-3-phosphate dehydrogenase [NAD(P)+] | low |
| Bbr_0341 | D-alanine--D-alanine ligase | low |
| Bbr_0360 | Holo-[acyl-carrier protein] synthase, belongs to 4-phosphopantetheinyl transferase superfamily | no |
| Bbr_0369 | Conserved hypothetical membrane spanning protein | no |
| Bbr_0370 | SSU ribosomal protein S15P | high |
| Bbr_0371 | Polyribonucleotide nucleotidyltransferase | high |
| Bbr_0379 | 16S rRNA processing protein rimM | high |
| Bbr_0380 | RNA binding protein | high |
| Bbr_0383 | Signal recognition particle, subunit FFH/SRP54 | no |
| Bbr_0385 | Cysteinyl-tRNA synthetase | low |
| Bbr_0386 | Glutamine amidotrasnferase Class I | low |
| Bbr_0393 | LSU ribosomal protein L32P | high |
| Bbr_0395 | Conserved hypothetical protein | no |
| Bbr_0396 | Phosphopantetheine adenylyltransferase | no |
| Bbr_0405 | Glucose-6-phosphate isomerase | high |
| Bbr_0408 | LSU ribosomal protein L19P | high |
| Bbr_0409 | Signal peptidase I | low |
| Bbr_0427 | Manganese transport protein mntH | low |
| Bbr_0434 | Oligosaccharide repeat unit transporter | medium |
| Bbr_0467 | Conserved hypothetical protein with a helix-turn-helix motif | medium |
| Bbr_0481 | Glycine cleavage system H protein | no |
| Bbr_0487 | Penicillin-binding protein | medium |
| Bbr_0498 | DNA-directed RNA polymerase beta chain | medium |
| Bbr_0499 | DNA-directed RNA polymerase beta' chain | high |
| Bbr_0510 | Metal-dependent hydrolase | no |
| Bbr_0512 | Phosphoglucosamine mutase | no |
| Bbr_0515 | Bacterial Peptide Chain Release Factor 2 (RF-2) | no |
| Bbr_0519 | SsrA-binding protein | no |
| Bbr_0524 | Glucosamine--fructose-6-phosphate aminotransferase | no |
| Bbr_0544 | Alanine racemase | no |
| Bbr_0546 | DNA primase | no |
| Bbr_0548 | Conserved hypothetical protein | no |
| Bbr_0567 | Phosphoribosylaminoimidazole carboxylase carboxyltransferase subunit | medium |
| Bbr_0568 | Phosphoribosylaminoimidazole carboxylase NCAIR mutase subunit | medium |
| Bbr_0598 | Phosphoribosylamidoimidazole-succinocarboxamide synthase | medium |
| Bbr_0604 | SSU ribosomal protein S12P | high |
| Bbr_0605 | SSU ribosomal protein S7P | high |
| Bbr_0606 | Protein Translation Elongation Factor G (EF-G) | high |
| Bbr_0607 | Protein Translation Elongation Factor Tu (EF-TU) | high |
| Bbr_0631 | Protein Translation Elongation Factor P (EF-P) | high |
| Bbr_0632 | N utilization substance protein B | high |
| Bbr_0636 | Guanylate kinase | high |
| Bbr_0646 | Glutamate racemase | low |
| Bbr_0652 | DNA-helicase-II-like protein | no |
| Bbr_0654 | Conserved hypothetical protein, contains Lon protease motif | medium |
| Bbr_0656 | Conserved hypothetical protein | medium |
| Bbr_0657 | 1-deoxy-D-xylulose 5-phosphate reductoisomerase | medium |
| Bbr_0658 | 1-hydroxy-2-methyl-2-(E)-butenyl 4-diphosphate synthase | medium |
| Bbr_0661 | Undecaprenyl pyrophosphate synthetase | medium |
| Bbr_0668 | NAD-dependent DNA ligase | low |
| Bbr_0669 | ATP-binding Mrp-like protein | low |
| Bbr_0670 | Glutamine synthetase | medium |
| Bbr_0671 | Conserved hypothetical membrane spanning protein | medium |
| Bbr_0679 | Phenylalanyl-tRNA synthetase alpha chain | medium |
| Bbr_0680 | Phenylalanyl-tRNA synthetase beta chain | medium |
| Bbr_0691 | Transposase | no |
| Bbr_0700 | Tyrosyl-tRNA synthetase | medium |
| Bbr_0701 | Conserved hypothetical protein | medium |
| Bbr_0706 | Hypothetical protein | no |
| Bbr_0707 | ATP-NAD kinase | no |
| Bbr_0711 | Transcriptional regulator, GntR family | no |
| Bbr_0718 | Nicotinate-nucleotide adenylyltransferase | medium |
| Bbr_0722 | Peptidyl-tRNA hydrolase | medium |
| Bbr_0725 | Enolase | high |
| Bbr_0727 | Conserved hypothetical protein with DUF501 domain | medium |
| Bbr_0732 | Transcription elongation factor greA | medium |
| Bbr_0735 | Cell division transcription factor WhmD | medium |
| Bbr_0745 | Two-component response regulator | medium |
| Bbr_0747 | CarD-like transcriptional regulator | medium |
| Bbr_0748 | 2-C-methyl-D-erythritol 2,4-cyclodiphosphate synthase | medium |
| Bbr_0749 | Permease protein of ABC transporter system for metals | no |
| Bbr_0750 | ATP-binding protein of ABC transporter system for metals | no |
| Bbr_0752 | Bifunctional methylenetetrahydrofolate dehydrogenase/cyclohydrolase | no |
| Bbr_0753 | SSU ribosomal protein S1P | high |
| Bbr_0754 | Dephospho-CoA kinase | no |
| Bbr_0759 | Putative response regulator with RNA-binding domain | no |
| Bbr_0771 | Acetate kinase | high |
| Bbr_0773 | Ribose-phosphate pyrophosphokinase | medium |
| Bbr_0775 | GMP synthase | no |
| Bbr_0776 | Xylulose-5-phosphate/Fructose-6-phosphate phosphoketolase | high |
| Bbr_0777 | UDP-N-acetylglucosamine pyrophosphorylase | medium |
| Bbr_0785 | Glutamine-dependent NAD(+) synthetase | low |
| Bbr_0794 | ATP-dependent Clp protease proteolytic subunit | medium |
| Bbr_0795 | ATP-dependent Clp protease proteolytic subunit | medium |
| Bbr_0801 | Succinate dehydrogenase iron-sulfur protein | low |
| Bbr_0802 | Succinate dehydrogenase flavoprotein subunit | low |
| Bbr_0829 | Hypothetical protein | no |
| Bbr_0864 | Cyclomaltodextrinase | no |
| Bbr_0884 | ATP-dependent DNA helicase pcrA | no |
| Bbr_0888 | SSU ribosomal protein S4P | high |
| Bbr_0894 | Hypothetical protein | low |
| Bbr_0896 | Conserved hypothetical, possibly secreted protein | low |
| Bbr_0898 | Alanyl-tRNA synthetase | low |
| Bbr_0899 | Endonuclease involved in recombination | low |
| Bbr_0905 | CTP synthase | medium |
| Bbr_0907 | FeS assembly protein SufB | medium |
| Bbr_0908 | ATP-binding protein of ABC transporter system | medium |
| Bbr_0909 | FeS assembly protein SufC | medium |
| Bbr_0910 | Cysteine desulfurase/Selenocysteine lyase | medium |
| Bbr_0911 | IscU protein | medium |
| Bbr_0912 | Conserved hypothetical protein with DUF59 domain | medium |
| Bbr_0918 | Conserved hypothetical protein | low |
| Bbr_0920 | GTP-binding protein era | low |
| Bbr_0926 | LSU ribosomal protein L25P | high |
| Bbr_0930 | SSU ribosomal protein S20P | high |
| Bbr_0945 | Hypothetical protein | medium |
| Bbr_0946 | Conserved hypothetical protein | medium |
| Bbr_0993 | Conserved hypothetical protein | low |
| Bbr_0994 | Phosphoglycerate kinase | medium |
| Bbr_0995 | Triosephosphate isomerase | medium |
| Bbr_1002 | Transaldolase | high |
| Bbr_1003 | Transketolase | high |
| Bbr_1007 | Bacitracin resistance protein (Putative undecaprenol kinase) | medium |
| Bbr_1009 | Threonyl-tRNA synthetase | medium |
| Bbr_1020 | Phosphoribosylaminoimidazolecarboxamide formyltransferase/IMP cyclohydrolase | medium |
| Bbr_1022 | Ribosomal large subunit pseudouridine synthase B | low |
| Bbr_1023 | GTP-binding protein | low |
| Bbr_1025 | UTP--glucose-1-phosphate uridylyltransferase | low |
| Bbr_1026 | Conserved hypothetical protein | low |
| Bbr_1029 | Conserved hypothetical protein | low |
| Bbr_1031 | Conserved hypothetical membrane spanning protein | low |
| Bbr_1032 | Conserved hypothetical protein | no |
| Bbr_1034 | Signal transduction protein garA | medium |
| Bbr_1035 | Conserved hypothetical protein with DUF881 domain | medium |
| Bbr_1036 | Small basic protein | medium |
| Bbr_1037 | Conserved hypothetical protein with DUF881 domain | medium |
| Bbr_1041 | Ribulose-phosphate 3-epimerase | medium |
| Bbr_1066 | Conserved hypothetical protein with RelB antitoxin domain | low |
| Bbr_1084 | Conserved hypothetical protein with helix-turn-helix motif | low |
| Bbr_1085 | Hypothetical membrane spanning protein | low |
| Bbr_1101 | Phosphatidate cytidylyltransferase | medium |
| Bbr_1102 | Ribosome Recycling Factor (RRF) | medium |
| Bbr_1103 | Uridylate kinase | medium |
| Bbr_1104 | Protein Translation Elongation Factor Ts (EF-Ts) | high |
| Bbr_1105 | SSU ribosomal protein S2P | high |
| Bbr_1111 | Conserved hypothetical secreted protein | low |
| Bbr_1119 | Modification methylase | low |
| Bbr_1121 | Cytosine methyl transferase, RM methylase bbrUIIM | low |
| Bbr_1127 | DNA polymerase III, delta subunit | low |
| Bbr_1130 | Leucyl-tRNA synthetase | medium |
| Bbr_1135 | Pyridoxamine 5'-phosphate oxidase pdxH | high |
| Bbr_1157 | GTP pyrophosphokinase/Guanosine-3',5'-bis(Diphosphate) 3'-pyrophosphohydrolase | medium |
| Bbr_1158 | Deoxyuridine 5'-triphosphate nucleotidohydrolase | medium |
| Bbr_1159 | Conserved hypothetical protein | medium |
| Bbr_1162 | DNA gyrase subunit A | low |
| Bbr_1170 | DNA gyrase subunit B | medium |
| Bbr_1171 | RNA polymerase principal sigma factor hrdB | no |
| Bbr_1175 | 1-acyl-sn-glycerol-3-phosphate acyltransferase | medium |
| Bbr_1178 | Protein translocase subunit secA | medium |
| Bbr_1181 | RecA protein | no |
| Bbr_1183 | Transcriptional regulator | no |
| Bbr_1185 | CDP-diacylglycerol--glycerol-3-phosphate 3-phosphatidyltransferase | low |
| Bbr_1186 | Cell division protein ftsK | low |
| Bbr_1188 | tRNA delta(2)-isopentenylpyrophosphate transferase | low |
| Bbr_1213 | GTP-binding protein TypA/BipA | medium |
| Bbr_1228 | LSU ribosomal protein L20P | high |
| Bbr_1229 | LSU ribosomal protein L35P | high |
| Bbr_1230 | Bacterial Protein Translation Initiation Factor 3 (IF-3) | high |
| Bbr_1232 | Thiamin pyrophosphokinase | no |
| Bbr_1233 | Glyceraldehyde 3-phosphate dehydrogenase | medium |
| Bbr_1234 | Conserved hypothetical protein with YbaK / prolyl-tRNA synthetases associated domain | low |
| Bbr_1235 | 4-hydroxy-3-methylbut-2-enyl diphosphate reductase | no |
| Bbr_1238 | Conserved hypothetical protein | low |
| Bbr_1240 | UDP-N-acetylmuramoyl-L-alanyl-D-glutamate-- lysine ligase | low |
| Bbr_1241 | Cell wall biosynthesis-associated protein | low |
| Bbr_1244 | Folylpolyglutamate synthase/Dihydrofolate synthase | medium |
| Bbr_1254 | D-tyrosyl-tRNA(Tyr) deacylase | low |
| Bbr_1256 | UDP-N-acetylmuramate--alanine ligase | low |
| Bbr_1257 | UDP-N-acetylglucosamine--N-acetylmuramyl- (pentapeptide) pyrophosphoryl-undecaprenol N-acetylglucosamine transferase | low |
| Bbr_1258 | Cell division protein ftsW | low |
| Bbr_1259 | UDP-N-acetylmuramoylalanine--D-glutamate ligase | low |
| Bbr_1260 | Phospho-N-acetylmuramoyl-pentapeptide- transferase | low |
| Bbr_1261 | UDP-N-acetylmuramoyl-tripeptide--D-alanyl-D- alanine ligase | low |
| Bbr_1263 | Peptidoglycan synthetase FtsI, penicillin-binding protein | low |
| Bbr_1267 | ATP-dependent DNA helicase | low |
| Bbr_1273 | L-lactate dehydrogenase | high |
| Bbr_1278 | Glutamine synthetase | medium |
| Bbr_1296 | DNA polymerase III alpha subunit | low |
| Bbr_1299 | Lipoprotein signal peptidase | medium |
| Bbr_1300 | Conserved hypothetical protein containing a repeated sequence found in lipoprotein LPP | medium |
| Bbr_1301 | Conserved hypothetical membrane spanning protein with YGGT family | medium |
| Bbr_1302 | Conserved hypothetical protein with DUF552 domain | medium |
| Bbr_1303 | Cell division protein ftsZ | medium |
| Bbr_1305 | Glycyl-tRNA synthetase | low |
| Bbr_1327 | dTDP-rhamnosyl transferase rfbF | medium |
| Bbr_1328 | Conserved hypothetical membrane spanning protein | low |
| Bbr_1329 | Fused ATP binding protein and permease of ABC transporter | low |
| Bbr_1348 | Aspartyl-tRNA synthetase | medium |
| Bbr_1349 | Histidyl-tRNA synthetase | medium |
| Bbr_1356 | Negative regulator of genetic competence clpC/mecB | high |
| Bbr_1364 | 60 kDa chaperonin GroEL | high |
| Bbr_1377 | Adenylosuccinate lyase | no |
| Bbr_1378 | Conserved hypothetical membrane spanning protein in uncharacterized protein UPF0104 family | no |
| Bbr_1379 | DNA-binding protein HU | high |
| Bbr_1381 | Conserved hypothetical protein with DUF797 domain | no |
| Bbr_1382 | Inositol monophosphatase family protein | no |
| Bbr_1394 | Primosomal protein N' | no |
| Bbr_1396 | Methionyl-tRNA formyltransferase | no |
| Bbr_1399 | S-adenosylmethionine synthetase | medium |
| Bbr_1400 | Fic family protein | no |
| Bbr_1414 | Isoleucyl-tRNA synthetase | medium |
| Bbr_1439 | Conserved hypothetical protein | low |
| Bbr_1448 | Exodeoxyribonuclease VII large subunit | low |
| Bbr_1463 | Methionine aminopeptidase | high |
| Bbr_1467 | Prolyl-tRNA synthetase | low |
| Bbr_1471 | Oligoribonuclease | low |
| Bbr_1472 | Inosine-5'-monophosphate dehydrogenase | medium |
| Bbr_1473 | Undecaprenyl-phosphate alpha-N-acetylglucosaminephosphotransferase | low |
| Bbr_1474 | Sua5/YciO/YrdC/YwlC family protein | low |
| Bbr_1480 | Peptide release factor-glutamine N5-methyltransferase | no |
| Bbr_1481 | Bacterial Peptide Chain Release Factor 1 (RF-1) | no |
| Bbr_1482 | LSU ribosomal protein L31P | high |
| Bbr_1490 | ATP-binding protein of ABC transporter system | no |
| Bbr_1498 | tRNA(Ile)-lysidine synthetase TilS | no |
| Bbr_1502 | N-acetylglucosaminyltransferase | no |
| Bbr_1523 | MerR family regulatory protein | no |
| Bbr_1532 | DNA processing chain A | no |
| Bbr_1563 | Dihydrofolate reductase | low |
| Bbr_1564 | Thymidylate synthase | low |
| Bbr_1569 | Conserved hypothetical protein with CHAP domain | medium |
| Bbr_1573 | Phosphate transport system protein phoU-like protein | medium |
| Bbr_1574 | Phosphoglycerate mutase | medium |
| Bbr_1576 | Lysyl-tRNA synthetase | medium |
| Bbr_1591 | Seryl-tRNA synthetase | low |
| Bbr_1593 | Transcription antiterminator, BglG family | medium |
| Bbr_1594 | PTS system, fructose/glucose-specific IIABC component | medium |
| Bbr_1599 | Ribonuclease HI | no |
| Bbr_1600 | Ribose 5-phosphate isomerase | medium |
| Bbr_1604 | Riboflavin kinase/FMN adenylyltransferase | no |
| Bbr_1606 | Ribosome-binding factor A | no |
| Bbr_1607 | Bacterial Protein Translation Initiation Factor 2 (IF-2) | medium |
| Bbr_1608 | N utilization substance protein A | low |
| Bbr_1614 | 50S ribosomal protein L17 | high |
| Bbr_1615 | DNA-directed RNA polymerase alpha chain | high |
| Bbr_1616 | SSU ribosomal protein S11P | high |
| Bbr_1617 | SSU ribosomal protein S13P | high |
| Bbr_1618 | LSU ribosomal protein L36P | high |
| Bbr_1619 | Bacterial Protein Translation Initiation Factor 1 (IF-1) | high |
| Bbr_1620 | Adenylate kinase | medium |
| Bbr_1621 | Protein translocase subunit secY | medium |
| Bbr_1622 | 50S ribosomal protein L15 | high |
| Bbr_1623 | 50S ribosomal protein L30 | high |
| Bbr_1624 | 30S ribosomal protein S5 | high |
| Bbr_1625 | 50S ribosomal protein L18 | high |
| Bbr_1626 | 50S ribosomal protein L6 | high |
| Bbr_1627 | 30S ribosomal protein S8 | high |
| Bbr_1628 | 30S ribosomal protein S14-1 | high |
| Bbr_1629 | 50S ribosomal protein L5 | high |
| Bbr_1630 | 50S ribosomal protein L24 | high |
| Bbr_1631 | 50S ribosomal protein L14 | high |
| Bbr_1632 | 30S ribosomal protein S17 | high |
| Bbr_1633 | 50S ribosomal protein L29 | high |
| Bbr_1634 | 50S ribosomal protein L16 | high |
| Bbr_1635 | 30S ribosomal protein S3 | high |
| Bbr_1636 | 50S ribosomal protein L22 | high |
| Bbr_1637 | 30S ribosomal protein S19 | high |
| Bbr_1638 | 50S ribosomal protein L2 | high |
| Bbr_1639 | 50S ribosomal protein L23 | high |
| Bbr_1640 | 50S ribosomal protein L4 | high |
| Bbr_1641 | 50S ribosomal protein L3 | high |
| Bbr_1642 | 30S ribosomal protein S10 | high |
| Bbr_1648 | 30S ribosomal protein S9 | high |
| Bbr_1649 | 50S ribosomal protein L13 | high |
| Bbr_1651 | Conserved hypothetical protein with leucine rich repeat variant | no |
| Bbr_1664 | Ferredoxin | no |
| Bbr_1666 | UDP-N-acetylenolpyruvoylglucosamine reductase | no |
| Bbr_1668 | 10 kDa chaperonin GroES | high |
| Bbr_1675 | LSU ribosomal protein L12P (L7/L12) | high |
| Bbr_1676 | LSU ribosomal protein L10P | high |
| Bbr_1679 | Phosphate transport ATP-binding protein pstB | no |
| Bbr_1680 | Phosphate transport system permease protein pstA | no |
| Bbr_1681 | Phosphate transport system permease protein pstC | no |
| Bbr_1682 | Phosphate-binding protein | no |
| Bbr_1684 | Phosphate regulon sensor protein PhoR | no |
| Bbr_1686 | 5'-methylthioadenosine nucleosidase/S-adenosylhomocysteine nucleosidase | no |
| Bbr_1703 | 2-C-methyl-D-erythritol 4-phosphate cytidylyltransferase | no |
| Bbr_1706 | tRNA (Guanine-N1) -methyltransferase | no |
| Bbr_1709 | LSU ribosomal protein L28P | high |
| Bbr_1719 | Type I multifunctional fatty acid synthase | no |
| Bbr_1720 | Acetyl-/propionyl-CoA carboxylase beta chain | no |
| Bbr_1721 | Acetyl-/propionyl-CoA carboxylase alpha chain | no |
| Bbr_1723 | Conserved hypothetical protein with possible biotin-(acetyl-CoA carboxylase) ligase domain | low |
| Bbr_1725 | Transcriptional regulator | no |
| Bbr_1726 | LSU ribosomal protein L1P | high |
| Bbr_1727 | LSU ribosomal protein L11P | high |
| Bbr_1729 | Transcription antitermination protein nusG | high |
| Bbr_1730 | Protein translocase subunit secE | high |
| Bbr_1731 | Aspartate aminotransferase | low |
| Bbr_1734 | GTP-binding protein, GTP1/OBG family | no |
| Bbr_1735 | LSU ribosomal protein L27P | high |
| Bbr_1736 | LSU ribosomal protein L21P | high |
| Bbr_1737 | Ribonuclease G | no |
| Bbr_1754 | Arginyl-tRNA synthetase | low |
| Bbr_1757 | UDP-N-acetylglucosamine 1-carboxyvinyltransferase | medium |
| Bbr_1769 | Phosphoesterase | low |
| Bbr_1786 | UDP-galactopyranose mutase | no |
| Bbr_1788 | Glycosyltransferase involved in cell wall biogenesis | no |
| Bbr_1793 | ATP-binding protein ABC transporter system for polysaccharides | medium |
| Bbr_1794 | Permease protein of ABC transporter systemfor polysaccharides | medium |
| Bbr_1795 | Alpha-L-Rha alpha-1,2-L-rhamnosyltransferase/alpha-L-Rha alpha-1,3-L-rhamnosyltransferase | medium |
| Bbr_1796 | Glycosyltransferase | medium |
| Bbr_1797 | Glucose-1-phosphate thymidylyltransferase | medium |
| Bbr_1798 | dTDP-4-dehydrorhamnose 3,5-epimerase/dTDP-4-dehydrorhamnose reductase | medium |
| Bbr_1799 | dTDP-glucose 4,6-dehydratase | medium |
| Bbr_1803 | Transcriptional regulator, LytR family | low |
| Bbr_1808 | Kup system potassium uptake protein | medium |
| Bbr_1820 | Methionyl-tRNA synthetase | low |
| Bbr_1869 | Alpha-galactosidase | no |
| Bbr_1870 | tRNA-specific adenosine deaminase | no |
| Bbr_1875 | Deoxycytidine triphosphate deaminase | low |
| Bbr_1882 | SpoU rRNA methylase family protein | medium |
| Bbr_1885 | Conserved hypothetical protein | no |
| Bbr_1890 | ATP-binding protein of ABC transporter system for sugars | high |
| Bbr_1898 | Ribonucleoside-diphosphate reductase beta chain | no |
| Bbr_1899 | Ribonucleoside-diphosphate reductase alpha chain | no |
| Bbr_1900 | NrdI protein | no |
| Bbr_1901 | Glutaredoxin nrdH | no |
| Bbr_1904 | Conserved hypothetical protein with helix-turn-helix motif | no |
| Bbr_1909 | Conserved hypothetical protein | no |
| Bbr_1911 | Dimethyladenosine transferase | no |
| Bbr_1912 | 4-diphosphocytidyl-2-C-methyl-D-erythritol kinase | no |
| Bbr_1914 | tRNA nucleotidyltransferase | low |
| Bbr_1915 | Phosphohydrolase (MutT/nudix family protein) | low |
| Bbr_1916 | Conserved hypothetical secreted protein | low |
| Bbr_1917 | Conserved hypothetical membrane spanning protein with virulence factor mviN domain | low |
| Bbr_1918 | Thioredoxin reductase | low |
| Bbr_1923 | Inner membrane protein (Preprotein translocase subunit YidC) | high |
| Bbr_1925 | Ribonuclease P protein component | high |
| Bbr_1926 | LSU ribosomal protein L34P | high |
| tRNA1 | tRNA Ala | high |
| tRNA2 | tRNA Xle | high |
| tRNA3 | tRNA Leu | high |
| tRNA4 | tRNA Gly | high |
| tRNA5 | tRNA Glu | high |
| tRNA6 | tRNA Gln | high |
| tRNA7 | tRNA Ser | high |
| tRNA8 | tRNA Lys | high |
| tRNA9 | tRNA Lys | high |
| tRNA11 | tRNA Met | high |
| tRNA12 | tRNA Arg | high |
| tRNA13 | tRNA Gly | high |
| tRNA14 | tRNA Leu | high |
| tRNA15 | tRNA Thr | high |
| tRNA16 | tRNA Arg | medium |
| tRNA17 | tRNA His | high |
| tRNA19 | tRNA Leu | medium |
| tRNA20 | tRNA Leu | high |
| tRNA22 | tRNA Gln | high |
| tRNA23 | tRNA Ala | high |
| tRNA24 | tRNA Ala | high |
| tRNA25 | tRNA Arg | high |
| tRNA26 | tRNA Arg | high |
| tRNA27 | tRNA Leu | high |
| tRNA28 | tRNA Gly | high |
| tRNA29 | tRNA Cys | high |
| tRNA30 | tRNA Val | high |
| tRNA31 | tRNA Val | high |
| tRNA32 | tRNA Gly | high |
| tRNA33 | tRNA Pro | high |
| tRNA34 | tRNA Asn | high |
| tRNA35 | tRNA Asn | high |
| tRNA38 | tRNA Asp | high |
| tRNA39 | tRNA Phe | high |
| tRNA40 | tRNA Asp | high |
| tRNA41 | tRNA Glu | high |
| tRNA42 | tRNA Pro | high |
| tRNA43 | tRNA Ser | medium |
| tRNA44 | tRNA Ser | high |
| tRNA45 | tRNA Ser | high |
| tRNA47 | tRNA Thr | high |
| tRNA48 | tRNA tyr | high |
| tRNA49 | tRNA Val | high |
| tRNA50 | tRNA Val | high |
| tRNA52 | tRNA Trp | high |
| tRNA53 | tRNA Ala | high |
