# Supplementary material for: Global transcriptional landscape and promoter mapping of the gut commensal Bifidobacterium breve UCC2003
Source: BMC Genomics. 2017 Dec 28;18:991. doi: 10.1186/s12864-017-4387-x (PMC5746004; doi:10.1186/s12864-017-4387-x)
Supplement: Supplementary file 8 — B. breve regulatory RNA expression. Artemis plot showing the regulatory RNA transcription in B. breve of a) FMN, b) TPP, and c) YKOK elements. In all cases tiling array signals of forward (red) and reverse (blue) strand are indicated. (PDF 732 kb) [file 12864_2017_4387_MOESM8_ESM.pdf]

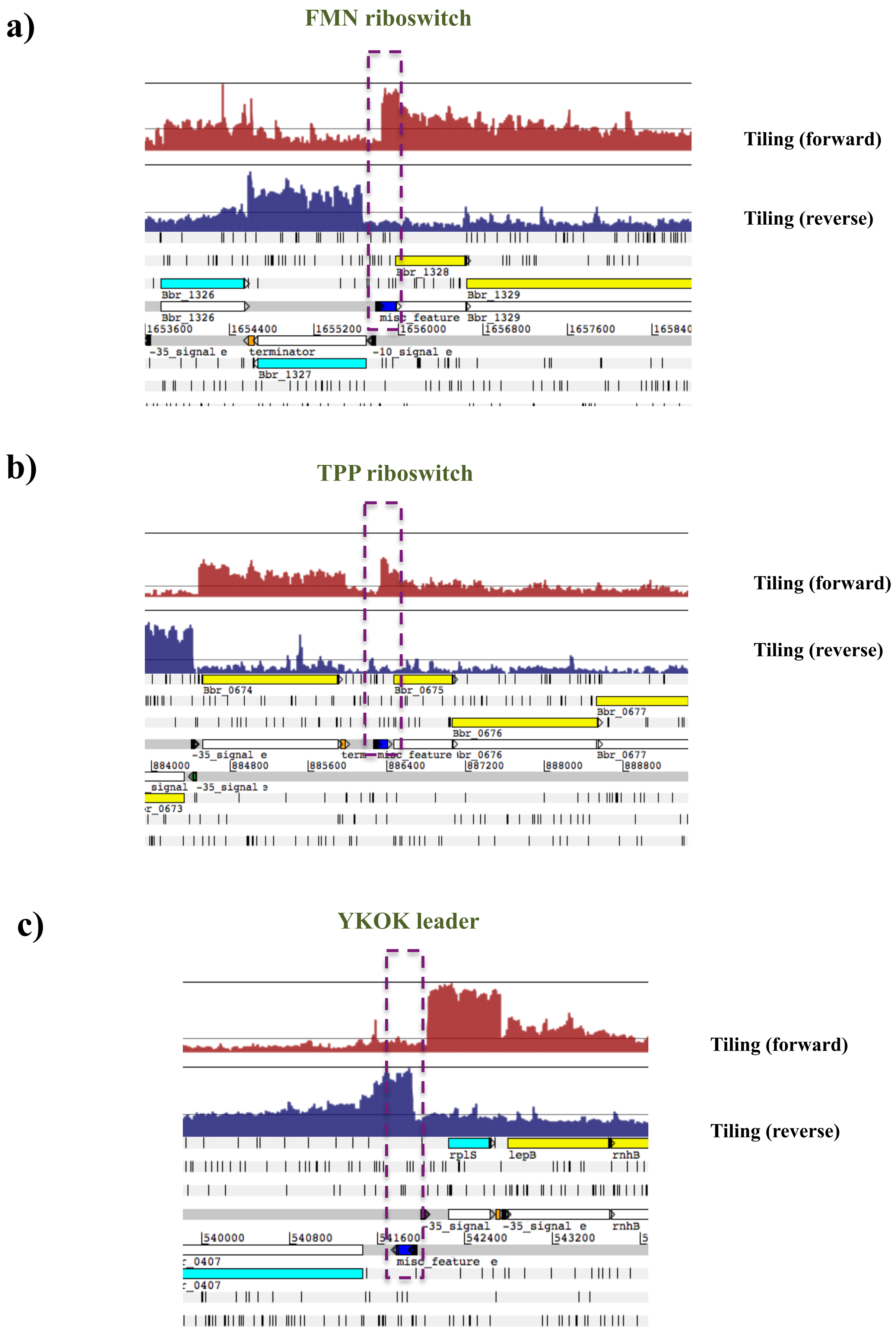
a)
FMN riboswitch
Tiling (forward)
Tiling (reverse)
Bbr_1328
Bbr_1326
Bbr_1329
misc_feature
1653600
1654400
1655200
1656000
1656800
1657600
-35_signal e
terminator
-10_signal e
Bbr_1327
b)
TPP riboswitch
Tiling (forward)
Tiling (reverse)
Bbr_0674
Bbr_0675
Bbr_0677
Bbr_0676
-35_signal e
misc_feature
884000
884800
885600
886400
887200
888000
888800
-35_signal
c)
YKOK leader
Tiling (forward)
Tiling (reverse)
rplS
lepB
rnhB
-35_signal
540000
540800
541600
542400
543200
misc_feature e
