# Supplementary material for: Global transcriptional landscape and promoter mapping of the gut commensal Bifidobacterium breve UCC2003
Source: BMC Genomics. 2017 Dec 28;18:991. doi: 10.1186/s12864-017-4387-x (PMC5746004; doi:10.1186/s12864-017-4387-x)

a)

Double-stranded rho-independent termination

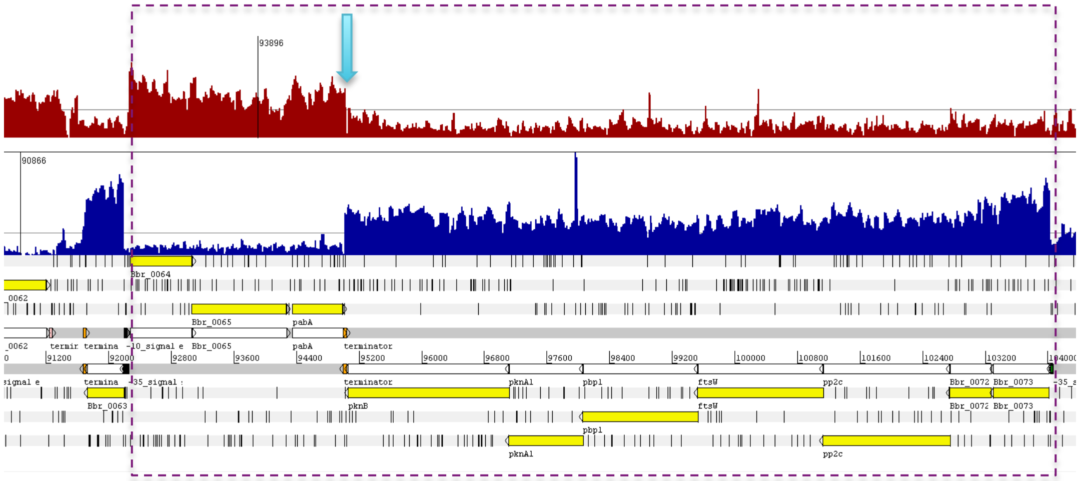

b)

Single-strand rho-independent termination

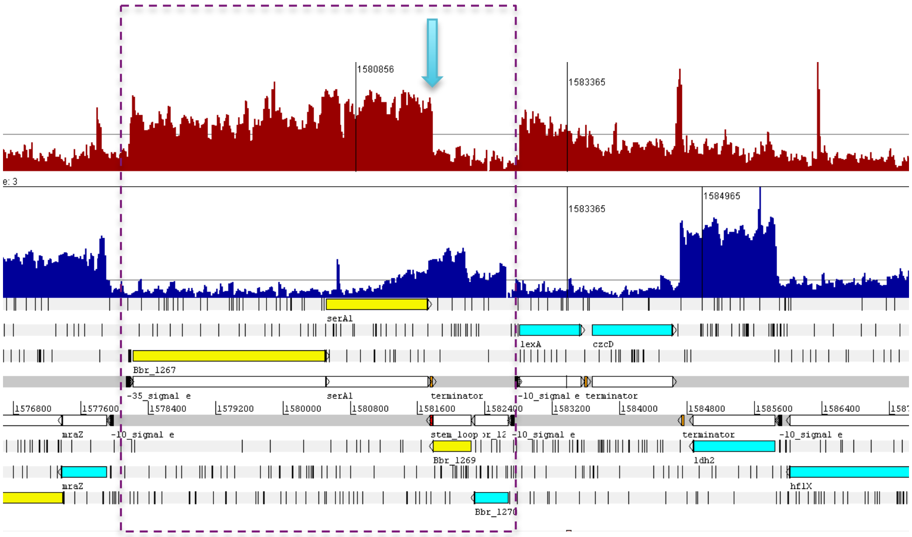

c)

Tail-to-tail convergent transcription

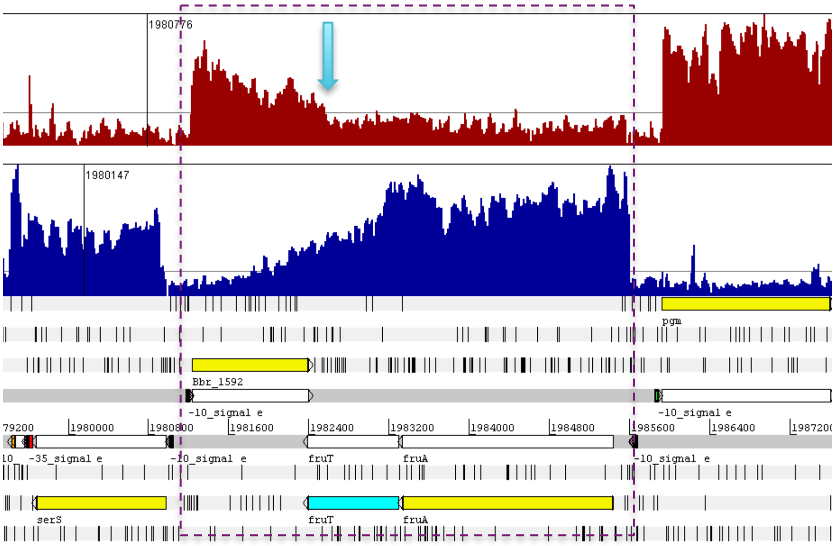

Supplement: Supplementary file 4 — B. breve termination of transcription. Artemis plot showing the different strategies of transcriptional termination of convergently expressed genes (dashed purple line) in B. breve: a) Double-stranded rho-independent termination observed between two convergently expressed genes employing a bidirectional terminator. In this case a clear-cut transcriptional termination is observed in either forward and reverse strands. b) Single stranded rho-independent termination observed between two convergently transcribed genes employing a strand specific terminator. In this case transcriptional termination is only observed in the forward strand. c) Tail-to-tail termination observed between two convergently transcribed genes without a termination signal. In this case no specific point of transcriptional termination can be observed. (PDF 474 kb) [file 12864_2017_4387_MOESM4_ESM.pdf]
